# Ligand-centred redox activation of inert organoiridium anticancer catalysts†

**DOI:** 10.1039/d0sc00897d

**Published:** 2020-05-15

**Authors:** Wen-Ying Zhang, Samya Banerjee, George M. Hughes, Hannah E. Bridgewater, Ji-Inn Song, Ben G. Breeze, Guy J. Clarkson, James P. C. Coverdale, Carlos Sanchez-Cano, Fortuna Ponte, Emilia Sicilia, Peter J. Sadler

**Affiliations:** Department of Chemistry, University of Warwick Coventry CV4 7AL UK p.j.sadler@warwick.ac.uk; Spectroscopy Research Technology Platform, University of Warwick Coventry CV4 7AL UK; Department of Chemistry and Chemical Technologies, University of Calabria via Pietro Bucci 87036 Arcavacata di Rende Cs Italy

## Abstract

Organometallic complexes with novel activation mechanisms are attractive anticancer drug candidates. Here, we show that half-sandwich iodido cyclopentadienyl iridium(iii) azopyridine complexes exhibit potent antiproliferative activity towards cancer cells, in most cases more potent than cisplatin. Despite their inertness towards aquation, these iodido complexes can undergo redox activation by attack of the abundant intracellular tripeptide glutathione (GSH) on the chelated azopyridine ligand to generate paramagnetic intermediates, and hydroxyl radicals, together with thiolate-bridged dinuclear iridium complexes, and liberate reduced hydrazopyridine ligand. DFT calculations provided insight into the mechanism of this activation. GS^−^ attack on the azo bond facilitates the substitution of iodide by GS^−^, and leads to formation of GSSG and superoxide if O_2_ is present as an electron-acceptor, in a largely exergonic pathway. Reactions of these iodido complexes with GSH generate **Ir-SG** complexes, which are catalysts for GSH oxidation. The complexes promoted elevated levels of reactive oxygen species (ROS) in human lung cancer cells. This remarkable ligand-centred activation mechanism coupled to redox reactions adds a new dimension to the design of organoiridium anticancer prodrugs.

## Abstract 2

Organometallic complexes with novel activation mechanisms are attractive anticancer drug candidates.

## Graphical abstract

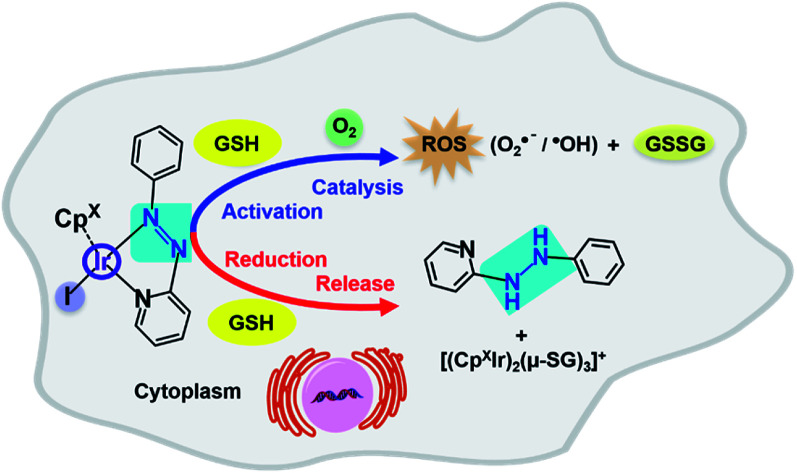

## Introduction

Three key platinum drugs, cisplatin, carboplatin, and oxaliplatin are widely administered in worldwide cancer chemotherapy.^1^ However, platinum resistance and undesirable side effects are now limiting their future use.^2^ Therefore, it is important to discover other metal complexes with different modes of action compared with platinum drugs.^3,4^ Third-row 5d^6^ iridium(iii) complexes offer potential structural diversity of octahedral coordination geometry, slow ligand exchange kinetics, and facile synthesis.^5–7^ For example, kinetically-inert octahedral iridium pyridocarbazole scaffolds can act as selective protein kinase inhibitors,^8^ and inert iridium polypyridine anticancer complexes can have targets other than DNA.^9^ Also, inert bis-cyclometalated iridium complexes are promising photosensitizers for singlet oxygen production,^10,11^ and a diselenobenzoquinone iridium complex which targets cytochrome P450 reductase, exhibits comparable potency to cisplatin.^12^

Half-sandwich organometallic iridium cyclopentadienyl complexes [(η^5^-Cp^X^)Ir(L^L′)Z]^*n*+/0^, where Cp^X^ = Cp* (pentamethyl-cyclopentadienyl), Cp^xph^ (tetramethyl(phenyl)-cyclopentadienyl), or Cp^xbiph^ (tetramethyl(biphenyl)-cyclopentadienyl), not only bind to DNA, but also target lysosomes and perturb the redox status of cells.^13–15^ The chelated ligand L^L′, the ancillary leaving group Z and the π-bound Cp^X^ ligand co-regulate the overall electronic structure and chemical reactivity of such iridium complexes. Reported half-sandwich iridium anticancer complexes with Z = Cl are labile and most likely activated in cancer cells through fast hydrolysis (minutes) of the chlorido ligand.^6^ Extension of the Cp^X^ ring from Cp* to Cp^xph^ and Cp^xbiph^ can slow down the hydrolysis rate, and increase the extent of hydrolysis.^16^ Rapid hydrolysis sometimes compromises anticancer activity due to rapid deactivation by side reactions in advance of reaching targets. For example, compared to the pyridine analogue [(η^5^-Cp^xbiph^)Ir(ppy)py]^+^ (ppy = 2-phenylpyridine), the chlorido complex [(η^5^-Cp^xbiph^)Ir(ppy)Cl] is more reactive, hydrolyzes more rapidly, and reacts readily with the abundant (*ca.* 0.5–10 mM) cellular thiol tripeptide glutathione (γ-l-Glu-l-Cys-Gly, GSH), but has only one third the potency of the pyridine analogue towards cancer cells.^13^ Hence, a major aim of this work is to optimise the potency of organo-iridium(iii) anticancer agents by rational control of their reactivity.

Here iodide is used as the monodentate ancillary leaving group, a ‘soft’ ligand expected to be strongly bound, and a poor leaving group.^17^ Iodido ligands are known to confer inertness towards hydrolysis of Ru/Os arene complexes,^18,19^ and increase potency towards cancer cell lines compared to chlorido analogues.^20^ Although many half-sandwich iodido iridium complexes with the N-heterocyclic carbene ligands have been studied,^21^ their chemical and biological mechanisms of action have been little explored.^22–24^ Also, the presence of the redox-active azopyridine ligands provides low-lying π* orbitals. Such ligands are known to participate in electron transfer processes for organometallic catalysts.^25–27^ Previously we have found that glutathione undergoes interesting reactions with phenyl-azo-pyridine ligands in arene Ru(ii) and Os(ii) complexes.^18,19,28^ The inclusion of an azopyridine ligand azpyNMe_2_ (*N*,*N*-dimethylphenyl-azopyridine) in a chlorido Cp^xph^ Ir(iii) complex produces a markedly different pattern of antiproliferative activity in over 800 cancer cell lines compared to 253 standard drugs, suggesting a novel mechanism of action.^29^

**Chart 1:**
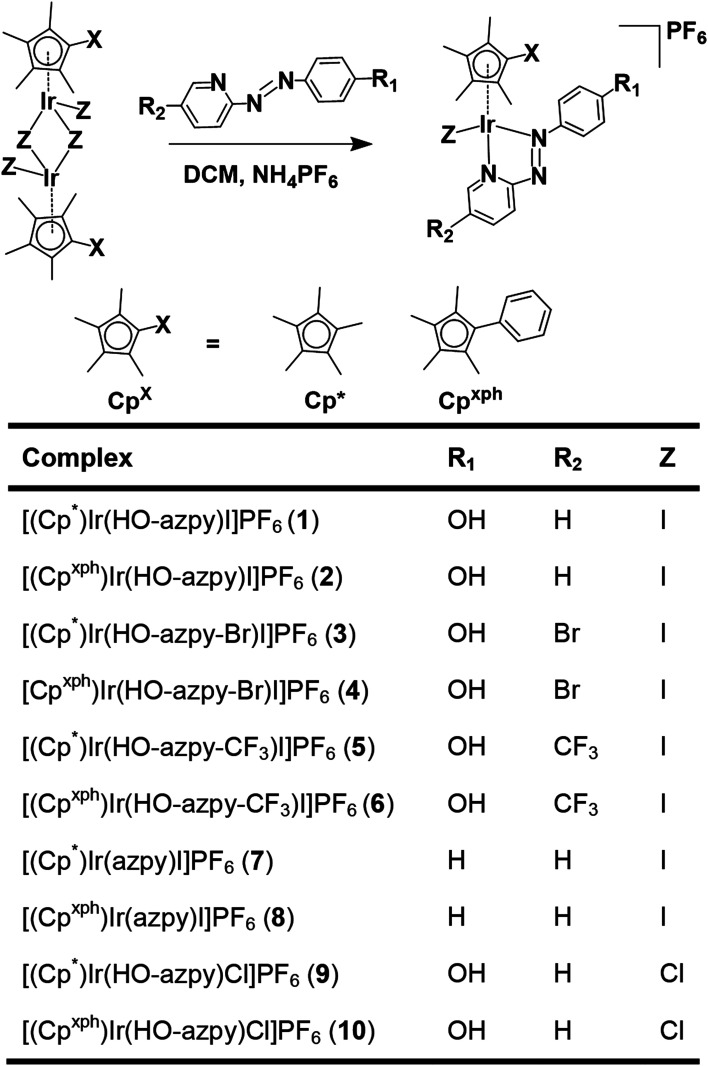
Synthesis route for the organoiridium(iii) complexes studied in this work, and formulae of the complexes (azpy = 2-phenylazopyridine). Crystals were obtained for complexes **1′**, **7′**, **8′** as iodido salts, and **9′** as a chlorido salt.

We have investigated the activation and reactivity of a small family of novel inert half-sandwich iodido organoiridium(iii) complexes bearing variously substituted bidentate phenyl-azopyridine ligands, and two chlorido analogues (Chart 1). The X-ray crystal structures of eight complexes have been determined. The chemical reactivity of these iodido complexes, as well as their antiproliferative activity against human lung cancer cells and ability to induce cellular reactive oxygen species (ROS) were also studied. The antiproliferative activity of iodido complex **1** against three other cancer cell lines and its toxicity *in vivo* towards zebrafish embryos compared to the chlorido analogue was determined.

The focus of this work is on elucidating the mechanism of activation of these inert iodido organoiridium anticancer complexes, especially their reactions with GSH, both experimentally and through DFT calculations. Intriguing is their ability to participate in both oxidative catalytic pathways and reductive azopyridine release pathways, features which have not been observed previously for other metals. This appears to be the first report of such reaction pathways for organoiridium anticancer complexes.

## Results

### Synthesis and characterization

Ten novel iridium(iii) complexes were synthesized in good yields by stirring mixtures of iridium chlorido/iodido dimer precursors with 2 mol equiv. of the appropriate chelating azopyridine ligand in dichloromethane (Chart 1). All the complexes were characterized by NMR spectroscopy (^1^H, ^1^H–^1^H COSY, ^13^C, HSQC, HMBC), elemental analysis and ESI-MS (details in S3.2 of the ESI†).

The complexes are chiral at the Ir(iii) centre. ^1^H NMR spectra of complexes **1** and **2** in d_4_-MeOD after addition of the anionic chiral shift regent Δ-trisphat^30,31^ at 298 K (Fig. S1 and S2†) show splitting of the aromatic proton signals in a *ca.* 1 : 1 ratio, indicating the presence of two enantiomers in equal abundance, similar to previously reported chiral osmium/ruthenium arene picolinamide and iminopyridine anticancer complexes.^32,33^

**Fig. 1:**
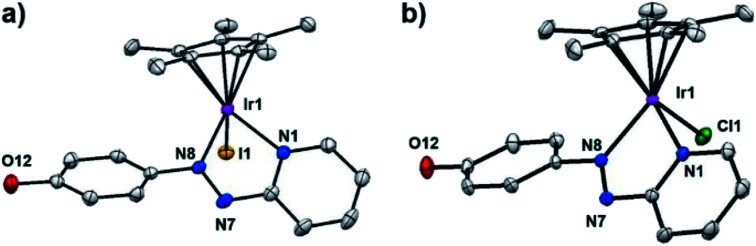
X-ray crystal structures of (a) [(η^5^-Cp*)Ir(HO-azpy)I]I·MeOH (**1′**·MeOH), (b) [(η^5^-Cp*)Ir(HO-azpy)Cl]Cl·MeOH (**9′**·MeOH), with thermal ellipsoids drawn at 50% probability. The hydrogen atoms, counter anions and solvent molecules have been omitted for clarity.

Single crystals suitable for X-ray diffraction of complexes **2**, **3**·MeOH, **5**·MeOH, **10** as PF_6_^−^ salts, **1′**·MeOH, **7′**, **8′** as iodido salts, and **9′**·MeOH as a chlorido salt, were obtained at ambient temperature by slow diffusion of Et_2_O into saturated methanol or dichloromethane (**8′**) solutions. X-ray crystal structures of complexes **1′**·MeOH and **9′**·MeOH are shown in Fig. 1, and the other six complexes in Fig. S3.† X-ray crystallographic data are listed in Tables S1 and S2,† and selected bond lengths and bond angles in Tables S3 and S4.† All the complexes adopt a typical pseudo-octahedral geometry with a “piano-stool” shape. The Ir–I bond lengths range from 2.6799(5)–2.6943(3) Å. Except for complex **5**·MeOH (1.870 Å) recorded at 296 K, the distances from the Cp^X^ ring centroid to iridium for all the other complexes recorded at 150 K are within 1.820 ± 0.009 Å, similar to the C^N chelated complex [(Cp*)Ir(ppy)Cl] (1.82 Å) (ppy = 2-phenylpyridine), but slightly longer than the N^N chelated complex [(Cp*)Ir(bpy)Cl]Cl (1.78 Å) (bpy = 2,2′-bipyridine).^16,34,35^ The N=N azo bond lengths are correspondingly lengthened to 1.27–1.29 Å from the uncoordinated mean length of 1.25 Å.^36^ In addition, Ir–N1 (pyridine nitrogen) bond is longer than that of Ir–N8 (azo nitrogen) in the iodido Cp* complexes, in contrast to chlorido Cp* complex **9′**·MeOH. However, in the iodido/chlorido Cp^xph^ complexes, Ir–N1 is of similar length to Ir–N8. The crystal structures of four complexes (**1′**·MeOH, **3**·MeOH, **5**·MeOH, and **10**) contain neutral phenol groups, while complexes **2** and **9′**·MeOH have a bridging proton shared between the phenoxide oxygens of neighboring complexes as illustrated in Fig. S4.†

Complexes **1–6** which contain a phenolic substituent on the azopridine ligand (Chart 1, R_1_ = OH) exhibit pH-dependent changes in their UV-vis absorption spectra in aqueous media (Fig. S5 and Table S5†), from which p*K*_a_ values of 3.90–6.49 were determined (Fig. S6 and S7†).

### Electrochemistry

Electrochemical reduction of iodido complexes **1**, **3** and **7** in comparison with unbound azpy (2-phenylazopyridine), HO-azpy (2-phenolazopyridine) ligands and iridium dimeric precursor [(Cp*)Ir(μ-I)I]_2_, was studied by cyclic voltammetry (CV) under N_2_. Complexes **1**, **3**, and **7** exhibited more facile sequential reduction than the corresponding free ligands or [(Cp*)Ir(μ-I)I]_2_. The first reduction step is quasi-reversible, ranging from −0.07 to −0.28 V. The second is irreversible, ranging from −0.58 to −0.79 V (Table 1, cyclic voltammograms shown in Fig. S8†).

**Table 1:** Electrochemical reduction potentials of selected ligands and complexes

Ligand/complex^a^	*E*_red_ (V)	Complex^a^	*E*_red_ (V)
Azpy	−1.07, −1.93	**1**	−0.28, −0.79
Azpy-OH	−0.94, −1.76	**3**	−0.13, −0.58
[Cp*Ir(μ-I)I]_2_	−1.21, −1.69	**7**	−0.07, −0.63

a Conditions: 1 mM free ligand or iridium complexes with 0.1 M Bu_4_NPF_6_ as supporting electrolyte in acetonitrile under N_2_ at ambient temperature, E_1/2_ (ferrocene/ferrocenium) = 0.063 V, scan rate = 0.1 V·s^−1^.

### Aqueous reactivity

The hydrolysis behavior of iodido complexes **1–8** in d_4_-MeOD/D_2_O (1/5 v/v) over 24 h was studied at 310 K by ^1^H NMR. No changes were observed in the spectra over this time. ESI-MS analysis of the NMR solutions showed only peaks assignable to cation [M − PF_6_]^+^ of the intact iodido complexes. To further verify that the ^1^H NMR spectrum contained only peaks for the intact iodido complexes, and that therefore no hydrolysis had occurred, complex [(Cp*)Ir(HO-azpy-Br)I]PF_6_ (**3**) in MeOD/D_2_O (1/1, v/v) was reacted with 1.2 mol equiv. AgNO_3_ at 310 K to remove the coordinated iodide. The resulting NMR peaks for the aqua species were assigned (Fig. S9a†) and contrasted with the hydrolysis-inert complex **3** (Fig. S9b†).

Reactions of complex [(Cp*)Ir(HO-azpy-Br)I]PF_6_ (**3**) with 3.0 mol equiv. nucleotide guanosine 5′-monophosphate (5′-GMP), nucleobase 9-ethylguanine (9-EtG), and amino acids l-histidine, *N*-acetyl-l-methionine, l-tryptophan, and l-arginine were also studied over 24 h at 310 K in 0.1 M phosphate buffer D_2_O/d_4_-MeOD (1/1, v/v, pH* 7.8) by ^1^H NMR and ESI-MS. No adduct was observed between complex **3** and 5′-GMP or 9-EtG, nor with l-Trp or l-Arg by NMR (Fig. S10†) or by ESI-MS. Only small amounts of adducts with *N*-acetyl-l-methionine (29%) or l-histidine (7%) were observed (Fig. S11†) based on ^1^H NMR peak integrals, and also evidenced by ESI-MS with assignable peaks for adducts (Table S6†).

The hydrolysis of the chlorido analogues was also investigated at 310 K for comparison. ^1^H NMR spectra of 100 μM solutions of [(Cp*)Ir(HO-azpy)Cl]PF_6_ (**9**) and [(Cp^xph^)Ir(HO-azpy)Cl]PF_6_ (**10**) in d_6_-DMSO/D_2_O (1/9, v/v) showed that these complexes hydrolyzed to the extent of 53% (Fig. S12†) and 66% (Fig. S13†), respectively, at equilibrium over 24 h. The aqua adduct of complex **9** was also detected by ESI-MS, giving a positive ion peak *m*/*z* at 543.99 corresponding to the formula [(Cp*)Ir(O-azpy)(H_2_O)]^+^ (**9**-H_2_O) (calcd 544.15). The hydrolysis of complex **9** followed pseudo first-order kinetics (Fig. S14†) with an hydrolysis rate constant of 0.00698 ± 0.00096 min^−1^ at 310 K and the half-life of 99.3 min determined by HPLC analysis. The chlorido complex **9** not only reacted with 9-EtG to the extent of 15% (Fig. S15†), but also almost completely formed adducts with *N*-acetyl-l-methionine and l-histidine (Fig. S16†) based on ^1^H NMR peak integrals. These adducts were also characterized by ESI-MS, shown in Table S6.†

Next, the stability of the iodido complex [(Cp*)Ir(HO-azpy-Br)I]PF_6_ (**3**) (1.0 mM) in MeOD/D_2_O (1/1, v/v, pH* 7.0) was studied in the presence of 4.0 mM, 23.0 mM, or 103.0 mM NaCl, physiologically relevant extracellular and intracellular concentrations, over 24 h at 310 K. Integration of the methyl ^1^H NMR peaks for Cp* at *δ* 1.73 ppm (**3**) and *δ* 1.59 ppm (**3-Cl**, the chlorido analogue of **3**), showed that the amount of **3-Cl** formed by iodide/chloride ligand exchange at these three NaCl concentrations was 2.7%, 7.8%, and 16.5%, respectively (Fig. S17†). LC-MS spectra for complex **3** at micromolar concentrations with the three different NaCl concentrations showed a positive-ion peak for the chlorido analogue **3-Cl** with a shorter retention time of *ca.* 17.6 min (*c.f. ca.* 21.0 min for **3**, Fig. S18†). However, when 100 μM **3** reacted with 103.0 mM NaCl, a higher amount of **3-Cl** (*ca.* 56%) was detected by HPLC peak integration (Fig. S18†).

In addition, ^1^H NMR spectra of the iodido and chlorido complexes in d_6_-DMSO were monitored over a time course of 19 d to investigate their stability at 298 K, since DMSO was used as a solvent in cell growth inhibition assays. However, no changes to any ^1^H NMR peaks were observed (Fig. S19 and S20†). ESI-MS data showed peaks only for the original complexes, providing evidence for the inertness of these iridium complexes towards solvolysis in DMSO.

### Reactions with *N*-acetyl-l-cysteine (NAC)

Initially reactions of complexes **1**, **3** and **7** with *N*-acetyl-l-cysteine (NAC), as an example of a biologically important thiol, were studied by ^1^H NMR spectroscopy. Time-dependent ^1^H NMR spectra of [(Cp*)Ir(HO-azpy)I]PF_6_ (**1**) (1 mM) with 3 mol equiv. NAC in d_4_-MeOD/0.1 M phosphate buffer D_2_O (3/7 v/v, pH* 7.4) at 310 K were recorded up to 24 h (Fig. S21†). In the aromatic region, a new set of ligand-based peaks appeared with the set of ligand peaks of iodido complex **1** decaying during the first hour (Fig. S21†). While in the aliphatic area, the peak for Cp* protons of complex **1** shifted completely from *δ* 1.71 to 1.59 ppm within the first hour, and two new sets of peaks in a 1 : 1 ratio for acetyl methyl protons of NAC appeared at *δ* 1.88 and 1.77 ppm (Fig. S21†).

**Fig. 2:**
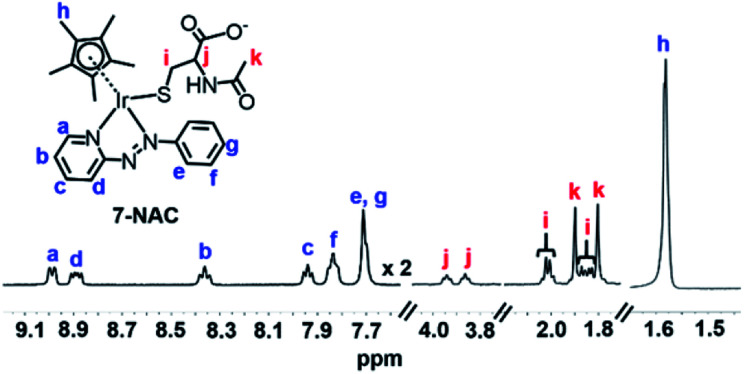
^1^H NMR spectrum (400 MHz, d_4_-MeOD/0.1 M phosphate buffer D_2_O, 3/7, v/v, pH* 7.4, 298 K) of isolated **7-NAC** adducts. The bound NAC (assigned as red i, j, and k), the Cp* ring methyl (assigned as blue h) and the protons assigned as a and d on the azo ligand show two sets of peaks due to the presence of diastereomers.

**Fig. 3:**
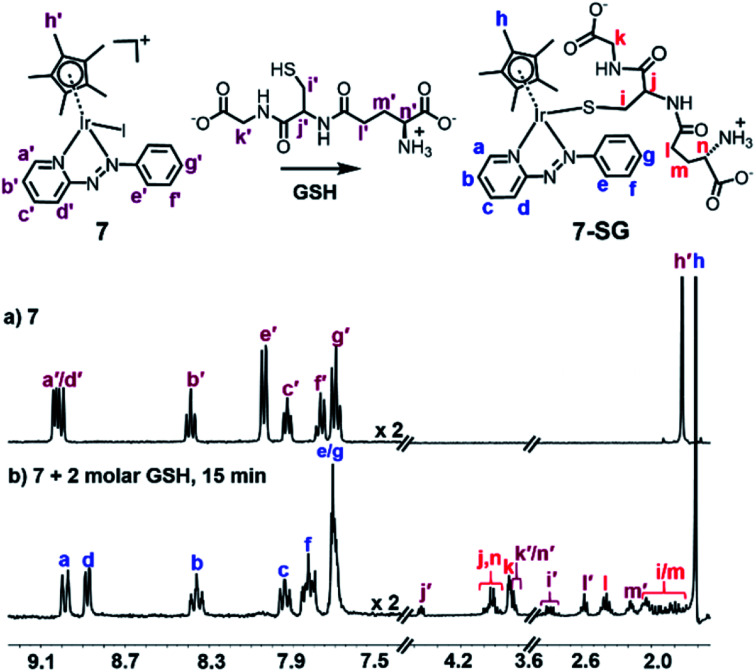
^1^H NMR spectra (400 MHz, d_4_-MeOD/0.1 M phosphate buffer D_2_O, 3/7 v/v, pH* 7.4) of (a) complex **7** (1 mM), and (b) 15 min after addition of 2 mol equiv. GSH (2 mM) at 310 K, showing complete formation of **7-SG**.

After reaction of **1** (100 μM) with 3 mol equiv. NAC for 1 h, LC-MS analysis showed a new ESI-MS peak with a shorter retention time of *ca.* 12.5 min with *m*/*z* 689.26, assignable as [(**1-NAC**)-I]^+^ with a bound deprotonated NAC thiolate (calcd 689.18, NAC is [CH_3_CONHCH(COOH)(CH_2_S)]^−^ in Fig. S22†). This confirmed that the new sets of NMR peaks for ligand, Cp* and NAC were from **1-NAC** adducts. The two sets of acetyl methyl peaks for bound NAC can be assigned to the diastereomers of **1-NAC** (Fig. S21†) due to the chirality of the iridium centre and NAC. Diastereomers were also evident in the ^1^H NMR spectra of isolated adducts of complex **7** (Fig. 2) and **3** (Fig. S23†) with NAC, as two sets of peaks. Similarly, all the ^13^C NMR signals showed equally intense pairs of peaks for **7-NAC** (details of ^13^C NMR assignments for **7-NAC** in S3.2 of the ESI†). This appears to be the first characterization of half-sandwich iridium NAC adducts.

In contrast, no adduct was observed by LC-MS upon incubation of complex **7** with 10 mol equiv. of the amino acid β-alanine under the same reaction conditions. Hence, the thiol group of NAC appears to be a crucial site for reaction with these iodido iridium complexes.

### Reactions with glutathione (GSH)

Next the reactivity of complex **7** with glutathione was investigated. In the ^1^H NMR spectrum of [(Cp*)Ir(azpy)I]PF_6_ (**7**) (1 mM) within 15 min of the addition of GSH (2 mM) in d_4_-MeOD/phosphate buffer D_2_O (0.1 M, 3/7 v/v, pH* 7.4) at 310 K, a new set of aromatic ligand peaks as well as a new set of GSH protons appeared (Fig. 3), and the Cp* methyl peak shifted from 1.70 to 1.53 ppm and split into two peaks indicative of diastereomers of [(Cp*)Ir(azpy)(SG)]^+^ (**7-SG**).

Meanwhile, HPLC separation of the NMR solution revealed a new peak with shorter retention time of *ca.* 12.0 min compared to the parent complex **7** (*ca.* 21.5 min, Fig. S24†). The new ESI-MS peak with *m*/*z* 817.4 (Fig. S25†) can be assigned as the glutathione thiolate adduct [(**7-SG**) + H]^+^ (calcd *m*/*z* 817.2).

**Fig. 4:**
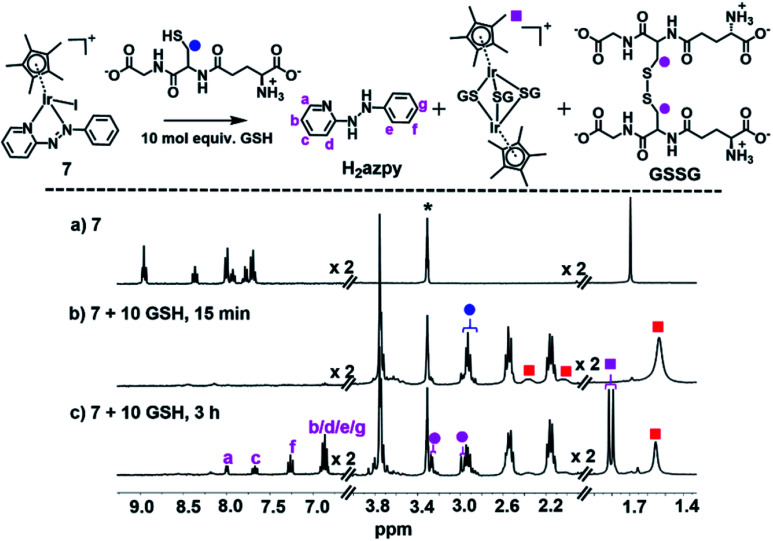
^1^H NMR spectra (400 MHz, d_4_-MeOD/0.1 M phosphate buffer D_2_O, 3/7 v/v, pH* 7.4, 298 K) of (a) complex **7** (1 mM), (b) 15 min, and (c) 3 h after reaction with 10 mol equiv. GSH at 310 K. The broadening of peaks in (b) can be ascribed to paramagnetic effects, and the new set of peaks in (c) assigned to the released phenyl-hydrazo-pyridine (H_2_azpy) and tri-SG bridged iridium dimer [(Cp*Ir)_2_(μ-SG)_3_]^+^. Red squares denote the **7-SG** adducts and * denotes residual CHD_2_OD.

When **7** was reacted with 10 mol equiv. of GSH under similar conditions, there was a dramatic loss in intensity of peaks in the aromatic region of the ^1^H NMR spectrum after 15 min, together with broadening of several peaks in the aliphatic region, shown in Fig. 4b, perhaps due to the presence of paramagnetic species. After 3 h, a new set of peaks appeared in the aromatic region combined with a 1 : 1 doublet at *δ* 1.79/1.82 ppm as well as new peaks at *δ* 3.30 ppm assignable to the β-CH_2_ of GSSG, Fig. 4c.

The main species in the NMR reaction mixture after 3 h were subsequently separated by HPLC and analyzed by positive-ion mass spectra. The MS peak at *m*/*z* 183.65 was assigned as unbound azpy ligand [(azpy) + H]^+^ (calcd 184.08), and the peak with *m*/*z* 185.64 as the two-electron-reduced product phenyl-hydrazo-pyridine [(H_2_azpy) + H]^+^ (calcd 186.10), Fig. S27.† In addition, an MS peak with *m*/*z* 786.96 was assigned as binuclear [(Cp*Ir)_2_(μ-SG)_3_ + 3H]^2+^ (calcd 787.19; Fig. S27†). These species indicated that after 3 h, the new set of ^1^H NMR aromatic peaks (Fig. 4c) was due to phenyl-hydrazo-pyridine arising from reduction and release of the azpy ligand, and the peaks at 1.79/1.82 ppm to the Cp* methyls of [(Cp*Ir)_2_(μ-SG)_3_]^+^.

### Radical trapping by EPR

**Fig. 5:**
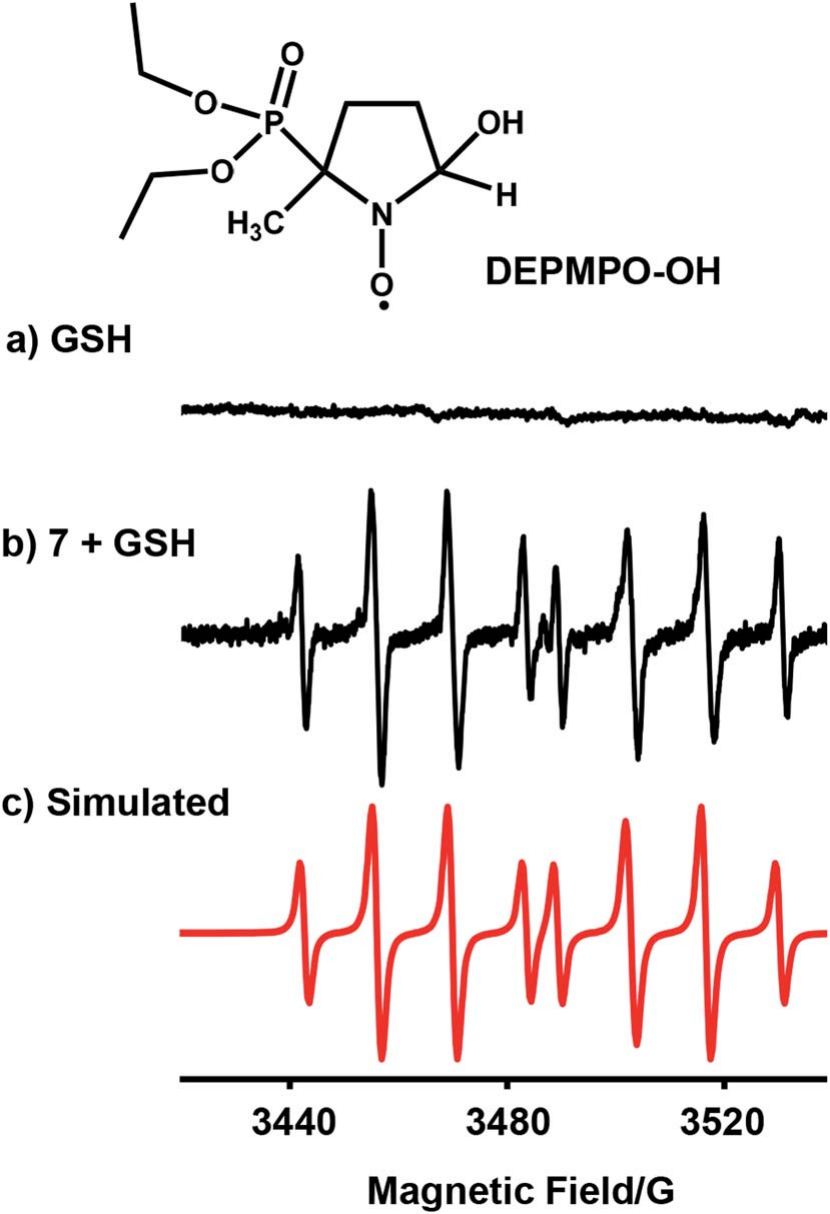
EPR spectra of (a) GSH alone; (b) radicals trapped by DEPMPO (100 mM) in the reaction mixture of complex **7** (1 mM) with GSH (20 mol equiv.) in 0.1 M phosphate buffer, pH 7.4, in the initial 87 min at 298 K; (c) simulated spectrum for trapped hydroxyl radicals DEPMPO-OH (a^N^ = 14.0 G, a^H^ = 13.2 G, and a^P^ = 47.3 G)^37^ using the EasySpin program.^38^

We investigated whether the NMR peak broadening (Fig. 4b) might be due to the presence of radicals and attempted to detect them by electron paramagnetic resonance (EPR) using a spin trap. EPR spectra of a solution containing **7** (1 mM) with GSH (20 mol equiv.) in phosphate buffer (0.1 M, pH 7.4) and the spin trap DEPMPO (Fig. 5b) or DMPO (Fig. S28†) showed a strong doublet of 1 : 2 : 2 : 1 quartets within the first 87 min, which decreased in intensity thereafter. These EPR signals are assignable by simulation to trapped hydroxyl radicals (Fig. 5c and S28†). GSH alone in the phosphate buffer (0.1 M, pH 7.4) (Fig. 5a) or complex **7** (1 mM) with GSH (20 mol equiv.) in phosphate buffer (0.1 M, pH 7.4) pre-deaerated with argon, were EPR-silent. In addition, when superoxide dismutase was added to the starting reaction mixture of complex **7** with GSH in aerated phosphate buffer, the formation of hydroxyl radicals was also not observed.

### Catalysis of GSH oxidation

**Fig. 6:**
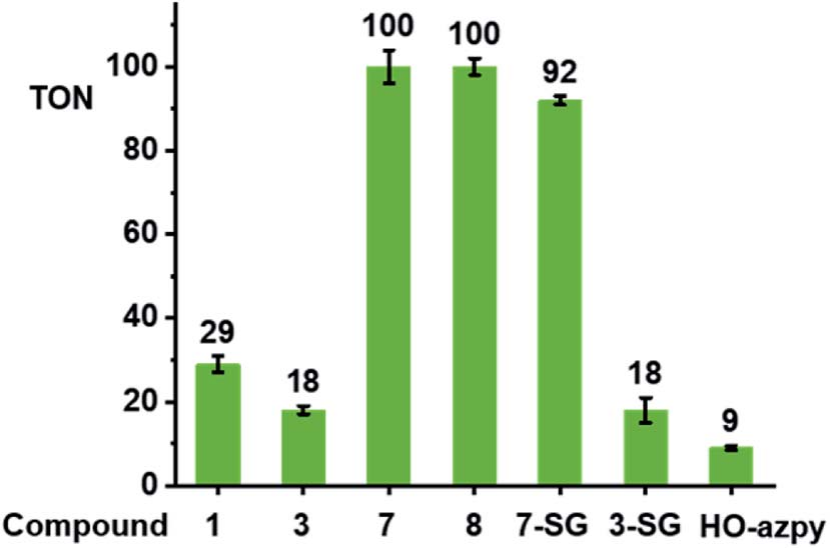
Turnover numbers for the catalytic oxidation of GSH by complexes **1**, **3**, **7**, **8**, **7-SG**, **3-SG** and free HO-azpy ligand. Reactions were carried out in d_6_-acetone/phosphate buffer D_2_O (5/95, v/v, 30 mM, pH* 7.4) at 310 K for 24 h and monitored by ^1^H NMR spectroscopy.

We investigated whether these [(Cp^X^)Ir(R_1_-azpy-R_2_)I]^+^ complexes can act as catalysts for the oxidation of GSH to GSSG. We monitored reactions with GSH (10 mM) for complexes with representative structural variability in the azopyridine ligand or the Cp^x^ ring, complexes **1** (Cp*, R_1_ = OH, R_2_ = H), **3** (Cp*, R_1_ = OH, R_2_ = Br), **7** (Cp*, R_1_ = R_2_ = H), and **8** (Cp^xph^, R_1_ = R_2_ = H) (Chart 1, 100 μM) in phosphate buffer (30 mM, pH* 7.4) at 310 K for 24 h by ^1^H NMR spectroscopy (Fig. S29†). The formation of GSSG was evident from the appearance of new peaks at *e.g. δ* 3.30 ppm corresponding to the β-CH_2_ of GSSG (Fig. S29†),^39^ and was further confirmed by the HPLC/LC-MS peak with *m*/*z* at 612.62 (calcd 613.15 for [GSSG + 3H]^+^), Fig. S30.† The turnover numbers (TONs) for complexes **7** and **8**, with unsubstituted azpy phenyl substituents, of 100 ± 4 and 100 ± 2, respectively, are much higher than those of the phenolate azpy complexes **1** and **3** (29 ± 2 and 18 ± 1, respectively, Fig. 6). By contrast, the free ligand phenol-azopyridine (HO-azpy) showed negligible catalytic activity (low TON of 9 ± 1, Fig. 6), and the GSH alone underwent negligible oxidation (Fig. S29†). No gas bubbles or pH changes were observed over 24 h incubation for the complexes, nor was H_2_O_2_ detected by peroxide test sticks (even with 1 mM catalyst).

To identify the active iridium catalyst, the reaction of complex **7** (100 μM) with GSH (10 mM) in phosphate buffer (30 mM, pH 7.4) was monitored over 24 h by HPLC/LC-MS. From the ESI-MS of the HPLC peaks, *ca.* 100% of **7** was converted to the glutathione adduct [(Cp*)Ir(azpy)(SG)]^+^ (**7-SG**) within the first 3 min after mixing, and this was the major species in the solution over 24 h (Fig. S31†). Hence the **Ir-SG** adduct appears to be the active catalyst in GSH oxidation. This was further confirmed by studying reactions of isolated complexes **7-SG** or **3-SG** (100 μM) with GSH (10 mM) under similar conditions (Fig. S32†). These reactions gave TONs of 92 ± 1 and 18 ± 3, respectively, exhibiting similar catalytic activity as the iodido complexes **7** and **3**.

Since the EPR studies indicated that oxygen is involved in the generation of hydroxyl radicals during these reactions, the reaction of complex **7** with GSH was carried out under oxygen-depleted conditions, which decreased the TON by *ca.* 83% to 17 ± 2 after 24 h at 310 K. Furthermore, the final reaction mixture analyzed by LC-MS, gave peaks assignable to the free azpy ligand, the free (reduced) H_2_azpy ligand, and the dinuclear adduct [(Cp*Ir)_2_(μ-SG)_3_]^+^ (Fig. S33†). These are different from the major product peak which was for **7-SG** (Fig. S31†) under aerated conditions.

### DFT simulation of aquation and GSH reactions

The X-ray crystal structure of the [(Cp^xph^)Ir(HO-azpy)I]PF_6_ complex (**2**) was used as a starting point for DFT calculations (details in S2.22 of the ESI†). DFT calculations suggested that all the investigated lowest energy pathways involve deprotonated GSH (GS^−^). Although the p*K*_a_ of the Cys thiol in GSH is high (*ca.* 9.4),^40^ there will be a small but significant amount (*ca.* 0.3%) of deprotonated GSH present at pH 7.^41^

**Fig. 7:**
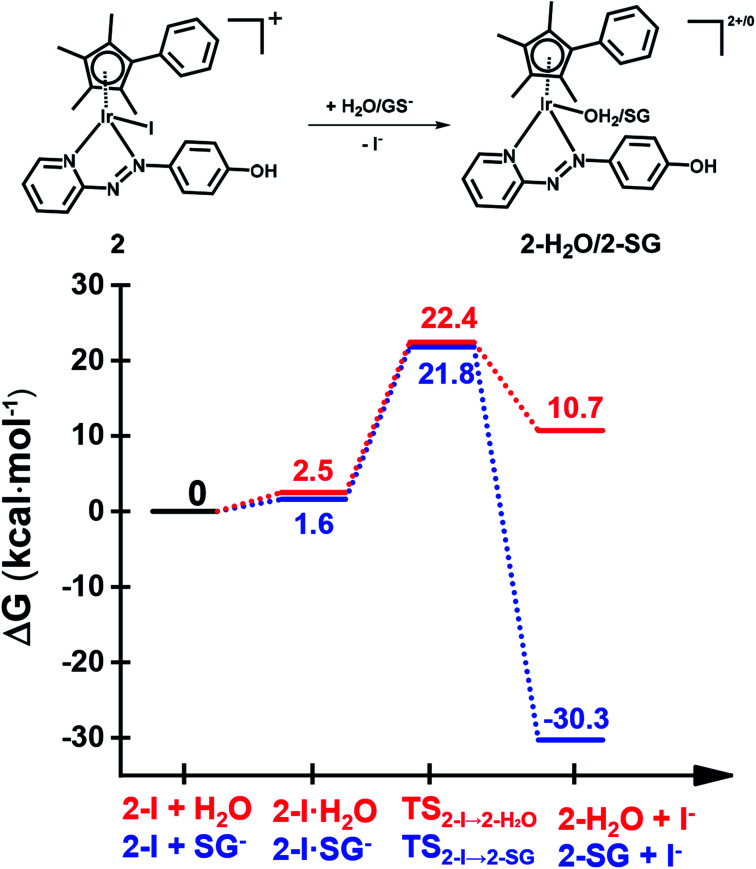
DFT calculated free energy profiles for direct substitution of iodide in complex **2** by water (aquation) or GS^−^. Relative energies are in kcal mol^−1^ and calculated with respect to separated reactants.

DFT calculations were conducted first to establish whether direct attack of H_2_O or GS^−^ on the Ir centre to displace the iodido ligand can occur. That is, after initial formation of slightly less stable adducts between **2** and H_2_O or GS^−^, the reactions proceed by second-order nucleophilic substitution (S_N_2). The activation barriers for formation of the transition states for these associative attacks are similar (19.9 *vs.* 20.2 kcal mol^−1^, Fig. 7). In contrast, the thermodynamics for displacement of the iodide by H_2_O or GS^−^ are different, being endergonic by 10.7 kcal mol^−1^ for H_2_O, and exergonic by 30.3 kcal mol^−1^ to form **Ir-SG**. Therefore, the calculations support the experimental finding that the complex is inert towards hydrolysis, and that direct GS^−^ attack is at least thermodynamically accessible.

### Attack of GSH on the azo bond

**Scheme 1:**
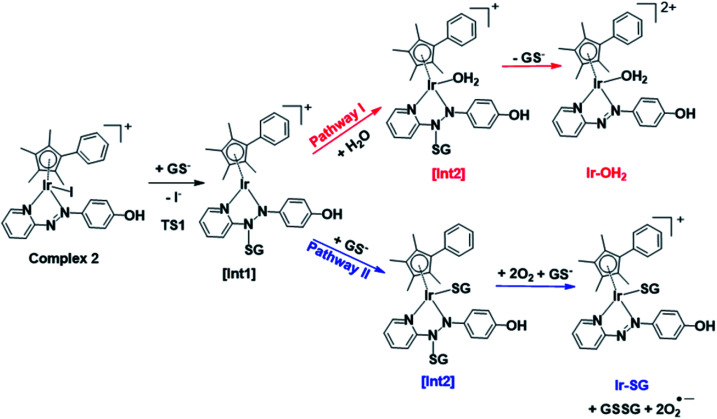
Pathways **I** and **II** for the formation of **Ir-OH2** and **Ir-SG**, as investigated by DFT calculations. Intermediates (abbreviated as [Int]) and products involve the reversible attack of GS^−^ on the azo bond.

DFT exploration of possible, less energy-demanding, alternative pathways for the substitution of iodide by GS^−^ or H_2_O showed that the reversible attack of GS^−^ on the N=N bond of the azopyridine ligand can assist in the process (Scheme 1).

**Fig. 8:**
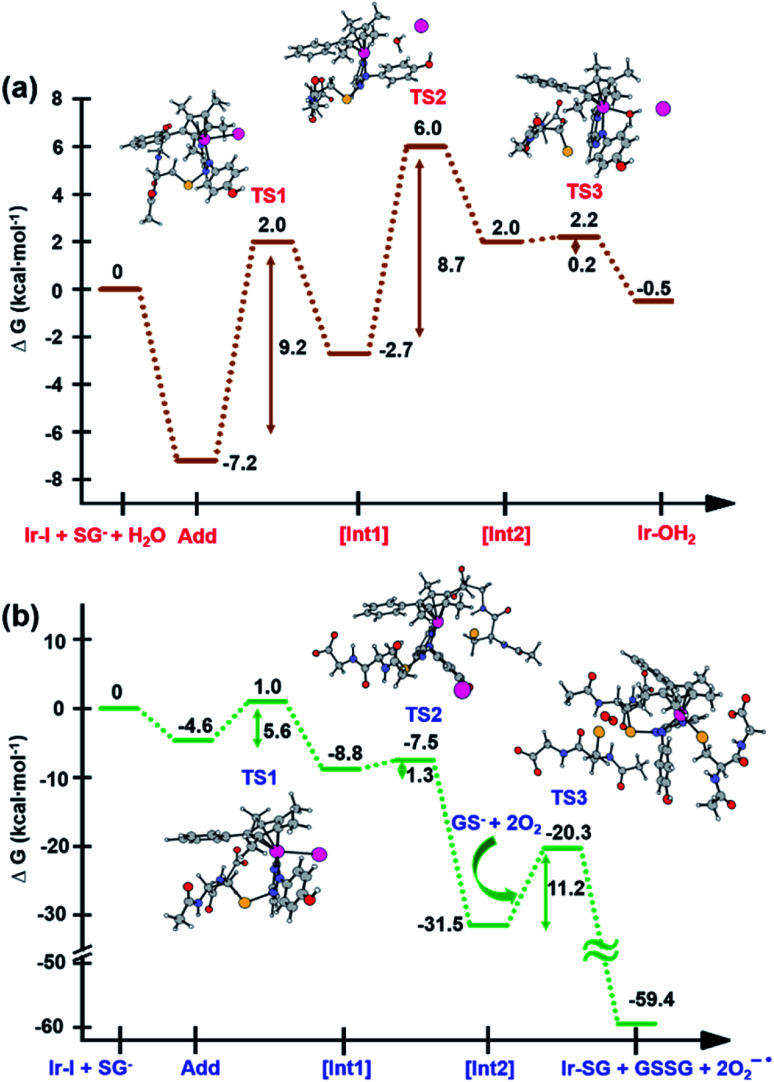
Calculated free energy profiles describing iodide substitution by (a) water in pathway **I**, and (b) GS^−^ in pathway **II**. Calculated energies are in kcal mol^−1^ with respect to reference energy of separated reactants.

Along pathway **I** for the substitution of iodide by H_2_O in Fig. 8a, the attack of GS^−^ on the non-coordinated N atom of the azo double bond, involves a barrier of only 9.2 kcal mol^−1^ to reach the transitional state TS1. Then water coordination to the vacant site on Ir (TS2 in Fig. 8a) occurs by surmounting an energy barrier of 8.7 kcal mol^−1^. To complete the substitution of I^−^ by water, detachment of GS^−^ from the N atom (the TS3 in Fig. 8a) corresponds to a very low energy barrier of only 0.2 kcal mol^−1^. The whole reaction is almost thermoneutral.

Along pathway **II** for the substitution of iodide by GS^−^ in Fig. 8b, the first step involving attack by GS^−^ on the N atom of the azo bond and the simultaneous release of the iodide ligand occur by surmounting a much low energy barrier of 5.6 kcal mol^−1^. The free energy profile for the next step (coordination of the second GS^−^ to iridium) is very flat with a barrier of only 1.3 kcal mol^−1^ to the transitional state TS2 in Fig. 8b. With respect to pathway **I**, attack of water on the iridium centre (Fig. 8a), the GS^−^ bound to the N atom of the azo group in pathway **II** is not spontaneously released. Instead, in the presence of a third GS^−^, the detachment of GS^−^ from the N atom occurs to form oxidized glutathione, GSSG, which involves the transfer of two electrons to other molecules. Computations suggest that the ground state O_2_ (^3^Σ) molecule can act as an electron acceptor for this step. Indeed, the O–O bond elongates, consistent with formation of superoxide O_2_^−^˙, whereas the second electron is localized on the N atom of N–N single bond which was previously bound to GS^−^. The whole system is in a triplet state again, and the spin is conserved. The singlet multiplicity of the complex can be recovered due to the presence of a second O_2_ molecule to form a second superoxide. In pathway **II** of Scheme 1, the final products are the singlet state **Ir-SG** complex, GSSG, and two O_2_^−^˙ radicals, to conserve the triplet multiplicity. Overall, this reaction is largely exergonic.

### Antiproliferative activity

**Fig. 9:**
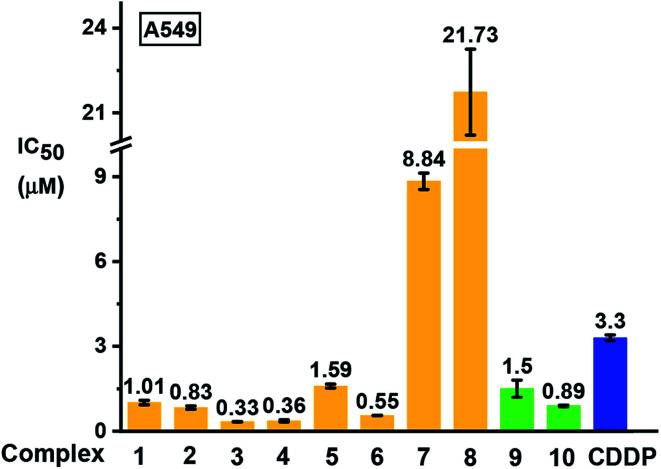
Inhibition of growth of A549 human lung cancer cells *in vitro* by complexes **1–10** in comparison with cisplatin (CDDP). The values of half maximal growth inhibitory concentration (IC_50_) are given as the mean ± standard deviations for a duplicate of triplicate experiments with cancer cells exposed to the tested complex for 24 h followed by recovery for 72 h in fresh complex-free medium. See Table S7† for full numerical data.

The antiproliferative activity of the complexes towards human lung A549 cancer cells was determined using the SRB assay^42^ and compared with cisplatin (CDDP), Fig. 9 and Table S7.† Iodido complexes **1–6** and chlorido complexes **9** and **10**, which share the common feature of a phenolic substituent on the azopyridine ligand, are highly potent with IC_50_ values (the concentration that inhibits cell growth by 50%) in the range 0.3–1.6 μM. Particularly potent is complex [(Cp*)Ir(HO-azpy-Br)I]PF_6_ (**3**) with an IC_50_ value of 0.33 μM, 10× more active than cisplatin. By contrast, complexes **7** and **8** bearing an unsubstituted azpy ligand are less active than other iodido complexes **1–6**. The substitution of one methyl group on Cp* by a phenyl substituent to give Cp^xph^ does not enhance the anticancer activity for iodido complexes, while the Cp^xph^ chlorido complex **10** is slightly more active than its Cp* analogue **9**.

Based on their promising *in vitro* activity, the potent iodido complex **1** and its chlorido analogue **9** were selected for further screening against human CNE2 (nasopharyngeal), A2780 (ovarian), and A2780cisR (ovarian) cancer cell lines to study the influence of the halido ligand on the anticancer activity. A2780cisR is cisplatin-resistant through reduced drug transport, enhanced DNA repair/tolerance and elevated GSH levels.^43^ Iodido complex **1** exhibits similar potency to chlorido analogue **9** against A549, CNE2, and A2780 cell lines, both more potent than cisplatin (see IC_50_ values in Table 2). Surprisingly, iodido complex **1** is *ca.* 2× more potent than the chlorido analogue **9** towards the cisplatin-resistant A2780cisR cell line. Both complexes are not cross-resistant with cisplatin, with resistance factors (IC_50_(A2780cisR)/IC_50_(A2780)) for **1** and **9** of 0.2 and 0.95, respectively. This suggests that these iodido complexes have a different mechanism of action compared to cisplatin.

**Table 2:** Antiproliferative activity of iodido complex **1** and chlorido analogue **9** towards human A549 lung, CNE2 nasopharyngeal, A2780 ovarian, and cisplatin-resistant A2780cisR ovarian cancer cells for 24 h exposure to the complexes and subsequent 72 h cell recovery in fresh complex-free medium

Complex	A549	CNE2	A2780	A2780cisR (RF)^a^
[(Cp*)Ir(HO-azpy)I]PF_6_ (**1**)	1.01 ± 0.08	1.26 ± 0.04	0.25 ± 0.02	0.049 ± 0.001(0.20)
[(Cp*)Ir(HO-azpy)Cl]PF_6_ (**9**)	1.5 ± 0.3	2.3 ± 0.3	0.12 ± 0.04	0.114 ± 0.003 (0.95)
Cisplatin	3.3 ± 0.1	7.7 ± 0.3	1.2 ± 0.2	11.5 ± 0.3 (6.41)

a Resistance factor RF = IC_50_(A2780cisR)/IC_50_(A2780).

### *In vivo* toxicity studies

To compare the *in vivo* toxicity of an iodido complex and its chlorido analogue, the LC_50_ lethal concentration (concentration which is lethal to half of the population) towards zebrafish (*Danio rerio*) embryos was determined for potent iodido complex **1** and its chlorido analogue **9**. This high-throughput vertebrate model is often used as a predictor for drug toxicity in humans.^44–47^ Strikingly, iodido complex **1** (LC_50_ = 0.26 ± 0.08 μM) was *ca.* 25× less toxic than its chlorido analogue **9** (LC_50_ = 0.010 ± 0.003 μM), although both are more toxic than cisplatin (LC_50_ = 0.6 ± 0.2 μM).^48^

### ROS detection

**Fig. 10:**
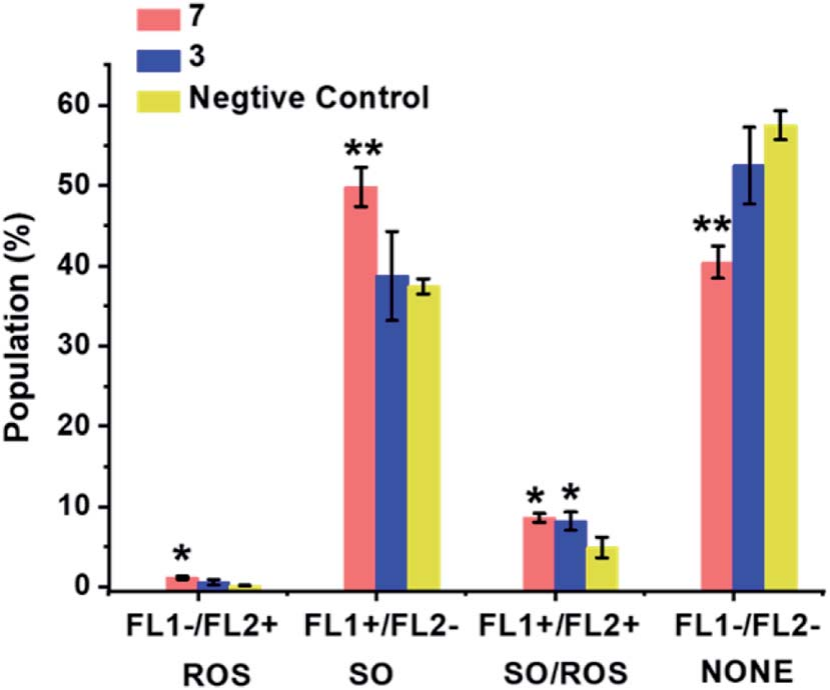
ROS induction in A549 cancer cells exposed to complex **3** or **7** at 2× IC_50_ concentrations for 24 h with untreated cells as the negative control. FL1 channel detects superoxide production, and FL2 channel detects total oxidative stress. Normalized population data are presented as the mean ± SD of triplicate samples for one experiment. *p*-Values were calculated after a *t*-test against the negative control data, *p < 0.05, **p < 0.01. See Table S8† for full numerical data.

ROS generation was compared for the most cytotoxic complex, **3**, and one of the least cytotoxic complex **7**. The aim of this study was to assess whether there is a direct correlation between *in vitro* cytotoxicity and ROS generation. The levels of reactive oxygen species (ROS) in A549 human lung cancer cells treated with complex **3** or **7** were determined at equipotent 2× IC_50_ concentrations (Fig. 10 and Table S8†) by flow cytometry fluorescence analysis using a total ROS/Superoxide (SO) Detection Kit. Superoxide production was monitored by following the orange channel FL1, and total ROS species, including H_2_O_2_, peroxy and hydroxyl radicals, peroxynitrite and NO, were monitored by the green channel FL2. After exposure to the complexes for 24 h, an increase in ROS/SO levels in cells treated with **3** or **7** was observed, when compared to untreated cells. Furthermore, a burst of superoxide production was also observed in cells treated with complex **7**.

## Discussion

Iridium(iii) is a classically-inert low-spin 5d^6^ metal ion, exemplified by the half-life for coordinated aqua ligands exchange on [Ir(H_2_O)_6_]^3+^ of >300 years.^49^ However, it was apparent during our study of [Ir(Cp*)(*N*,*N*)Cl]^+^ anticancer complexes (*N*,*N* = *e.g.* phenanthroline),^16^ and a previous report^50^ that the introduction of a Cp* ligand into the coordination sphere can increase ligand exchange rates by many orders of magnitude. Here we show that the inertness of Ir(iii) can be restored even in Cp* complexes, when the *N*,*N*-chelated ligand is a strong π-acceptor, and the monodentate ligand is a ‘soft’ iodido ligand.

Our aim was to explore pathways by which such inert Cp* complexes might become activated in cancer cells, to shed light on possible mechanisms of action. In so doing we have discovered some unusual reaction pathways for half-sandwich organoiridium complexes, involving not only attack on the azo bond of coordinated azopyridine by glutathione, but also catalysis of GSH oxidation and generation of reactive oxygen species, and reductive release of the azopyridine ligand, suggesting that these iodido iridium complexes are likely to have a unique mechanism of action.

### Inertness of iodido complexes towards aquation

Transition metal anticancer complexes containing chlorido ligands, including cisplatin and Ru(ii) arene complexes, often undergo activation by hydrolysis, giving more reactive aqua adducts.^51^ For the iodido complexes studied here, due to the softer character of iodide, the Ir–I bonds have stronger covalency, strengthened by π-acceptor chelated azopyridine ligands, and become inert towards hydrolysis. This is paralleled by the more stable iodido Ru(ii)/Os(ii) azopyridine complexes compared to their chlorido counterparts.^20^ Thus chlorido complex **9** hydrolyses much more slowly than diamine and diimine N^N chelated chlorido half-sandwich Cp* Ir(iii) anticancer complexes.^16^

The inertness of iodido iridium complexes towards hydrolysis also leads to their much lower chemical reactivity towards the nucleobase 9-EtG and various amino acids compared to the chlorido analogue (Table S6†). After 24 h, *ca.* 56% of micromolar iodido complex **3** was converted into its chlorido analogue at high (extracellular) NaCl concentration (103 mM, Fig. S18d†), and therefore much of this and other iodido complexes would be expected to persist for transport into cells, where the concentration of NaCl is much lower. Thus activation of the iodido iridium complexes is likely to involve novel pathways, distinct from chlorido complexes, and have targets other than coenzyme NADH^6^ or DNA.^52^

### Enhanced antiproliferative activity of zwitterionic complexes

**Fig. 11:**
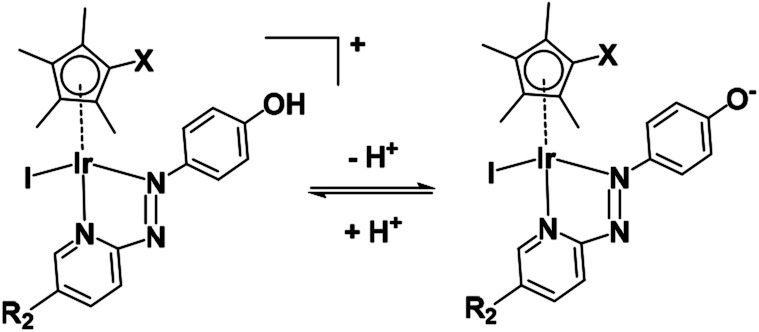
Acidity of complexes **1–6**.

On account of their low p*K*_a_ values (Fig. S7†), complexes **1–6** exist mainly in neutral zwitterionic forms (Fig. 11) at physiological pH (7.4) in cell culture media under cell-screening conditions.

These complexes all show higher anticancer potency against human A549 lung cancer cells than non-phenolic complexes **7** or **8** (Fig. 9). The anticancer activity of metal complexes often correlates with cellular uptake and lipophilicity.^53^ The accumulation of iridium in the lung cancer cells treated with the most potent complex [(Cp*)Ir(HO-azpy-Br)I]PF_6_ (**3**) was 2-fold higher than that in the cells treated with [(Cp*)Ir(azpy)I]PF_6_ (**7**) at equipotent IC_50_ concentrations (Table S9†). The *n*-octanol/water (pH 7.4) partition coefficients (log *P*_7.4_) for complexes **3** (−0.08) and **7** (−0.59), reveal that complex **3**, with p*K*_a_ of 6.31 (Fig. S7†) and being predominantly in its zwitterionic form, is more lipophilic relative to the +1-charged complex **7**. The higher lipophilicity contributes to the higher cellular iridium accumulation and anticancer activity of complex **3**.

### Antiproliferative activity and *in vivo* toxicity

The Ir(iii) analogue of the Ru(iii) clinical trial drug NAMI-A, *trans*-[IrCl_4_(DMSO)(Im)][ImH], (DMSO = dimethyl sulfoxide, Im = imidazole), exhibits low cytotoxic activity, attributable partly to its inertness towards hydrolysis.^54^ The cytotoxicity of Ru^II^ arene complexes is generally greater for complexes which hydrolyse relatively rapidly, and low for complexes which do not hydrolyse.^55^ However, for half-sandwich iridium complexes bearing C^N chelating ligands, replacement of Cl by pyridine decreases the rate of hydrolysis, but enhances the anticancer activity.^56^ In this work, replacement of Cl by I in the iridium azopyridine complexes does not result in loss of anticancer activity towards A549 lung cancer cells (complexes **1** and **9**, Chart 1 and Table 2). Remarkably, iodido complex **1** has 2-fold higher activity towards cisplatin-resistant A2780cisR human ovarian cancer cells with a much lower resistance factor than the chlorido analogue **9** (Table 2). This suggests that the potent iodido complexes have potential to overcome the cisplatin resistance.

The toxicity of iodido complex **1** towards zebrafish embryos is 25× lower than that of chlorido complex **9** which opens up a wider therapeutic window. However, this selectivity is lower than for scaffold-inert iridium pyridocarbazole complexes^57^ and Os(ii) azopyridine complexes,^48^ but there is scope in further work for improving this by changes in ligand substituents.

### GSH-mediated ligand-centred activation

These iodido iridium complexes are resistant towards hydrolysis and nucleobase binding, but exhibit more potent antiproliferative activity compared to the clinical anticancer drug cisplatin against A549 lung, CNE2 nasopharyngeal, and A2780 ovarian cancer cell lines (Fig. 9 and Table 2). Moreover, iodido complex **1** is more active than the chlorido analogue towards cisplatin-resistant A2780cisR ovarian cancer cells. The high potency of these inert iodido complexes raises intriguing questions about their activation mechanism.

GSH is an important cellular antioxidant which detoxifies various xenobiotics as well as protecting cells from toxic reactive oxygen species.^58^ Thus, reactions of metal complexes with GSH can perturb the redox state of cells. Unlike the detoxification of platinum drugs by conjugation with GSH, some chlorido Ru^II^ arene ethylenediamine anticancer complexes form Ru thiolate adducts which are not the dead-end products, but further oxidized to sulfenato and sulfinato adducts, facilitating the interaction of Ru complexes with DNA.^59^ Other Ru(ii),^60,61^ Os(ii),^18,28^ and Pt(iv)^62^ complexes have been reported to be activated by GSH. The high affinity of these iodido Ir(iii) azopyridine complexes for NAC or GSH to form Ir-thiolate adducts under physiologically relevant conditions is evident (Fig. 3 and 4).

DFT calculations show that displacement of iodide by GS^−^ to yield **Ir-SG** is thermodynamically favorable, being exergonic overall (Fig. 7). Regardless of the ratio between the iodido complex **7** and GSH, only the **Ir-SG** adduct is observed in the reaction (Fig. S22 and S24†). This is a different behavior from the iodido Os^II^ azopyridine complexes, which undergo hydrolysis to Os–OH species in the presence of equimolar GSH.^18^ In the present study, neither Ir–OH nor Ir–sulfenato species were observed.

The first-step in the electrochemical reduction of azo ligands can be assigned to the one-electron addition into the π* orbital centred on the azo group to give the azo anion radical.^63–65^ The second one-electron reduction gives rise to the dianionic species ([–N–N–]^2−^). In aqueous media, two-electron reduction is also accompanied by proton transfer to give hydrazo groups [–NH–NH–].^19^ The azo bond undergoes more facile reduction after metalation with Cp*-Ir (Table 1). The redox potential of GSH/GSSG (−240 mV at pH 7) in cells and tissues,^66,67^ is more negative than that of the azo bonds in the active iridium anticancer complexes (−130 mV and −70 mV for **3** and **7**, respectively, Table 1), suggesting that azo bonds in the ligands are likely to be reduced by GSH.

Furthermore, in the NMR study of reactions of complex **7** with 10 mol equiv. GSH under physiologically relevant conditions (Fig. 4b), there was a rapid disappearance (severe broadening) of the aromatic signals and the broadening of Cp* methyl peaks of the **7-SG** adducts after 15 min, which persisted for a few hours. This broadening may be due to ligand exchange reactions occurring at an intermediate rate on the NMR timescale, or to the formation of large, slow-tumbling polymeric species, or to paramagnetic species arising from redox reactions, such as azo ligand-centred radicals. The latter case seemed the most likely since the azo bond can take part in one- and two-electron reduction processes (Table 1).

EPR studies using spin traps for reactions carried out under similar conditions to the NMR study, confirmed the formation of hydroxyl radicals (Fig. 5). Since no radicals were detected in the presence of superoxide dismutase, or in the absence of O_2_, it is reasonable to assume that the hydroxyl radicals arise from superoxide, O_2_˙^−^, formed by the initial attack of glutathione on the N=N azo bond generating GS–N–N– species. It is known that trapped superoxide readily decomposes to hydroxyl radicals.^68^ After 3 h, the presence of 1.0 mol equiv. free phenyl-hydrazo-pyridine ligand and 0.5 mol equiv. tri-SG bridged di-iridium [(Cp*Ir)_2_(μ-SG)_3_]^+^ adduct in the solution, suggests that the azo group is the redox-active centre during the reactions of the complexes with GSH (Fig. 4c).

### Activation mechanisms by DFT calculations

DFT calculations revealed that the subsequent step after release of the iodido ligand, involves attack by a second GS^−^ on the Ir centre to form an **Ir-SG** complex requiring only 1.3 kcal mol^−1^ along pathway **II** (Scheme 1 and Fig. 8b), in contrast to the higher energy requirement of 8.7 kcal mol^−1^ for water binding to the Ir centre to give **Ir-OH2** along pathway **I** (Scheme 1 and Fig. 8a). Thus, after the attack of the first GS^−^ on the N atom of the azo bond, the energy-preferred sequence of steps is pathway **II** (Scheme 1), that is attack by a second GS^−^ on the Ir centre to form [(Cp*)(**Ir-SG**)(N-N-SG)] (the [**Int2**] of pathway **II**, Scheme 1), instead of [(Cp*)(Ir-OH_2_)(N-N-SG)]^+^ ([**Int2**] in pathway **I**, Scheme 1). Next, along pathway **II** the intermediate [(Cp*)(**Ir-SG**)(N-N-SG)] reacts with a third GS^−^ by the detachment of the GS^−^ from the nitrogen atom to form oxidized glutathione GSSG. One electron can be transferred to O_2_ as evidenced by the O–O bond elongation, and a superoxide anion is formed. Whereas, another electron is localized on the N atom previously attacked by GS^−^, which can be accepted by another oxygen molecule to form a second superoxide anion with the simultaneous regeneration of N=N bond in the **Ir-SG** adduct. The overall reaction is largely exergonic by 59.4 kcal mol^−1^. This thermodynamically favorable pathway **II** and involvement of oxygen in the activation pathway correlate well with the trapping of hydroxyl radicals by EPR as decay products of superoxide. Such a favorable attack of GSH on the azo bond is also consistent with the observation of iodide release in alkaline solution, where **Ir-SG** adducts are completely formed within minutes (Fig. S34†).

**Fig. 12:**
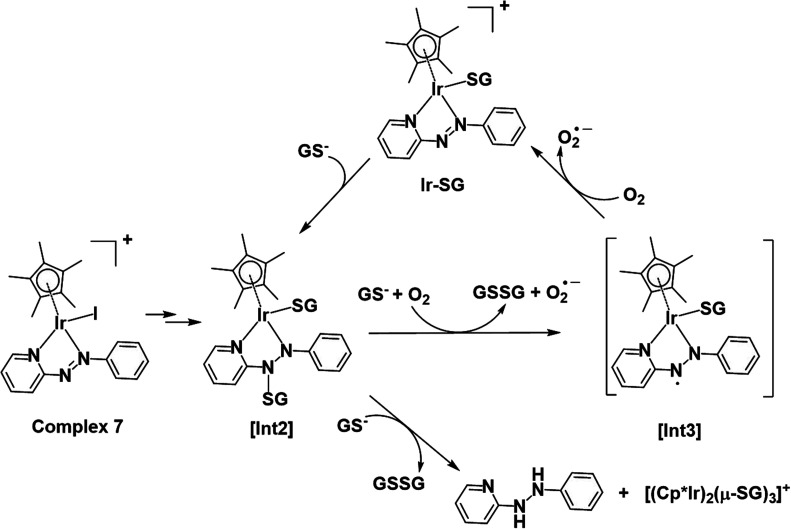
Proposed activation and catalysis pathways for the reaction of complex **7** with GSH following the DFT-calculated pathway **II** (Scheme 1). The paramagnetic species detected by NMR may be a ligand-centred radical species as indicated by [**Int3**].

Thus, the reaction pathways depicted in Fig. 12 account for the main features observed in the reaction of complex **7** with GSH. These include the presence of paramagnetic species (Fig. 4b) during the early stages (as a ligand-based radical [**Int3**]), the involvement of O_2_ as an electron acceptor in formation of **Ir-SG** adducts, the catalysis of GSH oxidation, and in the later stages, formation of reduced free hydrazopyridine together with thiolate-bridged dinuclear complexes (Fig. 4c). The formation of hydrazo products by consecutive reductions of azo bonds by thiols has been reported in the case of azo-bipyridine-bridged dinuclear ruthenium/iridium complexes,^69,70^ although it is notable that this appears not to be observed in the activation of iodido arene Ru(ii)^19^ and Os(ii)^18,28^ azopyridine complexes by GSH. Furthermore, the released phenyl-hydrazo-pyridine ligands and the dinuclear complex [(Cp*Ir)_2_(μ-SG)_3_]^+^ might not be dead-end products as they themselves may play a role in the biological activity. For example, the organic hydrazo compound procarbazine was approved as an anticancer drug in the late 1960s.^71,72^ Also Therrien *et al.* have reported that thiolate-bridged dinuclear Ir(iii) complexes exhibit high anticancer activity towards A2780 and A2780cisR ovarian cell lines.^73^

### Catalysis of GSH oxidation

Cp* iridium complexes are widely exploited as transfer hydrogenation catalysis with hydride donors such as formate or reduced nicotinamide adenine dinucleotide.^74^ Such reactions can lead to imbalance in redox homeostasis in cancer cells. However, the formation of catalytic Ir–hydride intermediates is likely to be inhibited in the presence of GSH, which is present in millimolar concentrations in cells. Catalysis of the oxidation of GSH to GSSG is another potential strategy for modulating cellular redox metabolism, although it has not been well studied. Iridium complexes of the type [(Cp^X^)Ir(N^N′)SH]^+^ are capable of oxidizing the GSH to GSSG, however, the mechanism has yet to be investigated.^75^ Thiolato-bridged iridium dimers are highly cytotoxic, but are poor catalysts.^73^ Here we have discovered that Cp* Ir(iii) azopyridine iodido complexes can catalyse oxidation of GSH to GSSG under physiologically relevant conditions (Fig. 6).

Based on the experimental data and DFT calculations, the mechanism for activation and catalysis shown in Fig. 12 for complex **7**, can be proposed. DFT calculated pathway **II** (Scheme 1) is largely exergonic, indicating that the activation of iodido complexes leads mainly to **Ir-SG** adducts. The azo bond of these **Ir-SG** complexes is attacked by excess GSH giving rise to GSSG and superoxide with oxygen as the one-electron acceptor. Molecular oxygen was shown to play a crucial role in the catalytic cycle since the TON number was lower under the oxygen-depletion condition and the generation of hydroxyl radicals detected by EPR required the presence of oxygen. Under the oxygen-depleted condition, the **Ir-SG** adducts readily decompose to give the free H_2_azpy ligand and dinuclear thiolato-bridged [(Cp*Ir)_2_(μ-SG)_3_]^+^ (Fig. 12). In the presence of O_2_ as the one-electron acceptor from the paramagnetic species [**Int3**], the **Ir-SG** adducts readily form, and participate in the catalytic cycle, thus leading to a higher catalytic efficiency (Fig. 12). ROS accumulation in A549 lung cancer cells treated with complex **7** (Fig. 10), suggests that such reactions can occur inside cells inducing the oxidative stress. Although the high catalytic activity of complexes **7** and **8** probably contributes to their high cytotoxicity, these complexes are not the most cytotoxic, suggesting that other pathways resulting in the release of reduced azopyridine ligand and dinuclear thiolato-bridged complexes may play important roles in the mechanism of anticancer activity.

## Conclusions

Iodido Ir(iii) Cp* azopyridine complexes exhibit potent cytotoxic activity towards cancer cells despite being relatively inert towards aquation or nucleobase binding. Some exhibit a 10-fold higher potency than the anticancer drug cisplatin against human lung cancer cells and are not cross-resistant. Also, complex **1** is 25-fold less toxic *in vivo* towards zebrafish embryos than its chlorido analogue.

Experiments and DFT calculations suggest that reactions with the abundant intracellular tripeptide glutathione play a major role in activation of these iodido complexes in cells and in generation of cytotoxic radicals (including superoxide), reduced azopyridine ligand and thiolato-bridged dinuclear complexes. A key feature in the activation is the attack of glutathione on the azo bond of the coordinated azopyridine. Reactions with GSH also give S-bound thiolato adducts which are catalysts for the oxidation of GSH to GSSG in oxygen-dependent mechanisms. It will be interesting in future work to investigate the dependence of cancer cell cytotoxicity on cellular oxygen concentrations because many tumours are hypoxic.

It will also be interesting to explore the distribution of the complexes and the active **Ir-SG** catalyst in cell compartments. High concentrations of glutathione are present not only in the cytoplasm, but also in mitochondria where it has crucial roles.^76^ Also the concentration of oxygen in mitochondria is expected to be high since this is the site of the electron transport chain which leads to oxidative phosphorylation. It will be interesting to investigate reactions between these complexes and proteins, which might be specific to those containing accessible free Cys residues.

This work suggests that iodido Ir(iii) Cp* azopyridine complexes have a unique multi-targeting anticancer mechanism of action, which is potentially important for combatting cisplatin resistance. The existence of both metal- and ligand-centred reactions provides wide scope for the design of novel organoiridium chemotherapeutic compounds.

## Conflicts of interest

There are no conflicts to declare.

## Supplementary Material

SC-011-D0SC00897D-s001

SC-011-D0SC00897D-s002
