# Supplementary material for: Ligand-centred redox activation of inert organoiridium anticancer catalysts
Source: Chem Sci. 2020 May 15;11(21):5466–80. doi: 10.1039/d0sc00897d (PMC8159363; doi:10.1039/d0sc00897d)
Supplement: SC-011-D0SC00897D-s001 [file SC-011-D0SC00897D-s001.pdf]

Electronic Supplementary Material (ESI) for Chemical Science.
This journal is © The Royal Society of Chemistry 2020

Electronic Supporting Information

Ligand-centred redox activation of inert organoiridium anticancer catalysts

Wen-Ying Zhang, Samya Banerjee, George M. Hughes, Hannah E. Bridgewater, Ji-Inn Song, Ben G. Breeze, Guy J. Clarkson, James P. C. Coverdale, Carlos Sanchez-Cano, Fortuna Ponte, Emilia Sicilia, and Peter J. Sadler*

Contents

S1. Materials
S2. Instruments and methods
S2.1 X-ray crystallography
S2.2 NMR spectroscopy
S2.3 Electrospray mass spectrometry
S2.4 Elemental analysis
S2.5 pH measurements
S2.6 UV-Vis spectroscopy
S2.7 Determination of pK_a
S2.8 Determination of molar extinction coefficients
S2.9 Determination of the hydrolysis kinetics for complex **9**
S2.10 Cyclic voltammetry
S2.11 High-performance Liquid Chromatography (HPLC) and Liquid Chromatography–Mass Spectrometry (LC-MS)
S2.13 Catalysis of GSH oxidation
S2.13 EPR experiments and simulation
S2.14 Partition coefficient determination
S2.15 Inductively Coupled Plasma-Optical Emission Spectroscopy (ICP-OES)
S2.16 Inductively Coupled Plasma-Mass Spectrometry (ICP-MS)
S2.17 Cell culture
S2.18 *In vitro* cell growth inhibition
S2.19 Toxicity in zebrafish
S2.20 Ir accumulation in cancer cells
S2.21 ROS determination
S2.22 Computational details
S3 Synthesis and characterization
S3.1 Synthesis of ligands and iridium precursors

S3.2 Synthesis of iridium complexes

Table S1. Crystallographic data for complexes **1'**·MeOH, **2**, **3**·MeOH, and **5**·MeOH.

Table S2. Crystallographic data for complexes **7'**, **8'**, **9'**·MeOH and **10**.

Table S3. Selected bond lengths and angles for iodido complexes **1'**·MeOH, **2**, **3**·MeOH, **5**·MeOH, **7'**, and **8'**.

Table S4. Selected bond lengths and angles for chlorido complexes **9'**·MeOH, and **10**.

Table S5. Absorption wavelengths and molar extinction coefficients for complexes **1-6** at pH 3 and 11**.**

Table S6. Reactions of complexes **3** and **9** with 9-EtG, N-acetyl-L-methionine (Ac-Met) and L-histidine (His).

Table S7. IC_{50} values for iridium complexes toward human A549 lung cancer cells in comparison with cisplatin (CDDP).

Table S8. ROS induction in A549 cancer cells treated with complexes **3** and **7**.

Table S9. Partition coefficients (log $P_{7.4}$) and the cellular iridium accumulation in A549 lung cancer cells of complexes **3** and **7**.

Figure S1. Enantiomers of complex **1** detected by ^{1}H NMR using Δ-trisphat.

Figure S2. Enantiomers of complex **2** detected by ^{1}H NMR using Δ-trisphat.

Figure S3. ORTEP views of the X-ray crystal structures for complexes **2**, **3**·MeOH, **5**·MeOH, **7'**, **8'** and **10**.

Figure S4. Tautomers in the crystal structure of **9'**·MeOH.

Figure S5. UV-vis spectra of complexes **1**, **3** and **5** at pH 3 and 11, and the pH-dependent changes of the UV-Vis spectrum for complex **1**.

Figure S6. Dependence on pH of the UV-vis absorbance at selected wavelengths of complexes **1-6** and **9**.

Figure S7. pK_a values of phenolic groups in iodido complexes **1-6** and chlorido complexes **9-10**.

Figure S8. Electrochemical cyclic voltammograms for selected ligands and complexes **1**, **3**, **7** and $[Cp^{*}Ir(\mu\text{-}I)I]_2$.

Figure S9. Inertness of complex **3** towards hydrolysis studied by ^{1}H NMR.

Figure S10. Inertness of complex **3** towards binding to nucleobases (9-EtG and 5'-GMP) studied by ^{1}H NMR.

Figure S11. Reactions of complex **3** with N-acetyl L-methionine and L-histidine studied by ^{1}H NMR.

Figure S12. Hydrolysis of complex **9** studied by ^{1}H NMR.

Figure S13. Hydrolysis of complex **10** studied by ^{1}H NMR.

Figure S14. Time-dependent HPLC study of the hydrolysis of complex **9** and the first-order kinetic fit.
Figure S15. Reaction of complex **9** with 9-EtG studied by ^{1}H NMR.
Figure S16. Reactions of complex **9** with N-acetyl L-methionine and L-histidine studied by ^{1}H NMR.
Figure S17. Reactions of iodido complex **3** under three NaCl concentrations studied by ^{1}H NMR.
Figure S18. HPLC chromatograms for reactions of complex **3** under three NaCl concentrations and MS peaks for the chlorido analogue.
Figure S19. Stability of complex **6** towards DMSO studied by ^{1}H-NMR.
Figure S20. Stability of complex **9** towards DMSO studied by ^{1}H-NMR.
Figure S21. Time-dependent ^{1}H NMR spectra for reaction of complex **1** with NAC.
Figure S22. HPLC chromatogram and MS peaks for **1-NAC**.
Figure S23. ^{1}H NMR spectrum of **3-NAC**.
Figure S24. HPLC chromatogram for **7-SG**.
Figure S25. ESI-MS peaks for **7-SG**.
Figure S26. Time-dependent HPLC chromatograms with detected ESI-MS peak for the reaction of complex **7** with 1 mol equiv. GSH.
Figure S27. LC-MS peaks for the reaction of complex **7** with 10 mol equiv GSH after 3 h (^{1}H NMR sample studied in Figure 4c).
Figure S28. EPR spectrum of hydroxyl radicals trapped and simulated as DMPO-OH from the reaction of complex **7** with GSH.
Figure S29. ^{1}H NMR spectra for the oxidation of GSH to GSSG catalyzed by complexes **1**, **3**, **7**, **8** and free phenol-azo-pyridine ligand.
Figure S30. Catalysis of GSH oxidation to GSSG by complex **7** under the aerated conditions studied by HPLC and ESI-MS.
Figure S31. Detection of **7-SG** formation by ESI-MS in time-dependent HPLC chromatograms for catalysis of GSH oxidation by complex **7**.
Figure S32. Catalysis of GSH oxidation to GSSG by **7-SG** and **3-SG** studied by ^{1}H NMR.
Figure S33. Catalysis of GSH oxidation by complex **7** under a nitrogen atmosphere studied by HPLC and ESI-MS.
Figure S34. Effect of iodide addition and pH on the formation of **7-SG** in reactions of complex **7** with GSH.

References

S1 Materials

$IrCl_3$ was purchased from Precious Metals Online. 1, 2, 3, 4, 5-Pentamethylcyclopentadiene, 2, 3, 4, 5-tetramethyl-2-cyclopentanone, butyllithium solution (1.6 M in hexanes), ammonium hexafluorophosphate, potassium iodide, sodium chloride, Chelex 100 sodium form, β-alanine, N-acetyl-L-cysteine, N-acetyl-L-methionine, L-histidine, L-tryptophan, L-arginine, 9-ethylguanine and guanosine 5'-monophosphate disodium salt hydrate, Δ-trisphat tetrabutylammonium salt, deuterium methanol and deuterium oxide, cisplatin, dimethyl sulfoxide (DMSO), and sulforhodamine B sodium salt were purchased from Sigma-Aldrich. Solvents of laboratory grade used for syntheses, HPLC grade solvents, HPLC grade trifluoroacetic acid (Htfa), and analytical grade disodium hydrogen phosphate dihydrate and disodium hydrogen phosphate dodecahydrate were obtained from Fisher Scientific. Reduced glutathione was obtained from Alfa Aesar and the iridium/platinum plasma standard (1000±10 μg/ml in 10% v/v HCl) were obtained from Inorganic Ventures. Concentrated nitric acid (72% v/v) was freshly distilled before use. ROS/superoxide detection kit was purchased from Enzo Life Sciences (UK). For ICP-MS and ICP-OES experiments, sodium chloride (TraceSELECT®) and type-1 Milli-Q water are used.

The A2780, A2780cisR (cisplatin resistant), A549 human lung, and CNE2 nasopharyngeal cell lines were purchased from the European Collection of Cell Cultures (ECACC). Roswell Park Memorial Institute (RPMI-1640) medium, fetal bovine serum, glutamine, penicillin/streptomycin mixture, trypsin, and phosphate-buffered saline (PBS) were purchased from PAA Laboratories GmbH.

S2 Instruments and Methods

S2.1 X-ray crystallography

Suitable crystals were selected and mounted on a glass fiber with Fomblin oil and placed on a Rigaku Oxford Diffraction SuperNova diffractometer with a dual source (Cu at zero) equipped with an AtlasS2 CCD area detector. The crystals were kept at 150 ± 2 K, except the crystal of complex **5**·MeOH kept at 296 ± 2 K, during data collection. Using Olex2,[1] all the structures were solved with the ShelXT[2] structure solution program using direct methods and refined with the ShelXL[3] refinement package using least squares minimization. X-ray crystallographic data for complexes **1'**·MeOH, **2**, **3**·MeOH, **5**·MeOH, **7'**, **8'**, **9'**·MeOH and **10** have been deposited in the Cambridge Crystallographic Data Centre under the accession numbers CCDC 1860992, 1860993, 1860994, 1860995, 1860996, 1860997, 1921724 and 1921725, respectively. X-ray crystallographic data in CIF format are available from the Cambridge Crystallographic Data Centre (http://www.ccdc.cam.ac.uk/).

S2.2 NMR spectroscopy

1D ^{1}H and ^{13}C NMR spectra and 2D ^{1}H-^{1}H gs (gradient selected) COSY (correlation spectroscopy), ^{1}H-^{13}C HMQC (heteronuclear multiple quantum coherence), and HMBC (heteronuclear multiple bond coherence) NMR spectra were obtained at 298 K (unless stated otherwise) on Bruker DPX 400 or AVIII 500M or AVIII 600 NMR spectrometers. ^{1}H NMR chemical shifts were internally referenced to the residual signals of the solvents. ^{13}C NMR chemical shifts were internally referenced to $CDCl_3$ (77.16 ppm) for chloroform-d_1, $(CD_3)_2SO$ (39.52 ppm) for DMSO-d_6, and CD_3CN (1.39 ppm) for acetonitrile-d_3 and CD_3OD (49.00 ppm) for methanol-d_4. The data were processed using MestReNova 5.3.1 and Topspin (version 2.1 Bruker UK Ltd.).

S2.3 Electrospray mass spectrometry

Electrospray ionization mass spectra (ESI-MS) were obtained for samples in methanol on a Bruker Esquire 2000 spectrometer. The mass spectra were recorded with a scan range of either *m/z* 50-500 or 400-1000 in positive ion mode.

S2.4 Elemental analysis

CHN elemental analyses were carried out on a CE-440 elemental analyzer by Warwick Analytical (UK) Ltd.

S2.5 pH measurements

pH or pH* (pH meter reading without correction for effect of deuterium on the sensor) values for samples in H_2O or D_2O were measured at *ca.* 298 K, using a HATCH minilab pocket pH meter with ISFET silicon chip pH sensor, calibrated with buffer solutions of pH 4, 7, and 10.

S2.6 UV-Vis Spectroscopy

A Cary 300 UV-vis recording spectrophotometer was used with 1 cm path-length quartz cuvettes (3.0 mL) and a PTP1 Peltier temperature controller. Spectra were recorded using UV Winlab software and plotted using Origin 2018. Experiments were carried out at 298 K from 800 to 200 nm with 0.5 nm intervals.

S2.7 Determination of pK_a

Changes in the UV-vis absorption spectra of 20-30 μM solutions of complexes **1-6** and **9-10** in 10% methanol/90% Milli-Q water in 1 cm path-length quartz cuvettes (3.0 mL) from pH 2-12, were monitored by UV-Vis spectroscopy, after addition of μL amounts of either dilute perchloric acid in acetic acid, or dilute KOH. Changes in absorbance maxima at different pH values were plotted and fitted to the Henderson-Hasselbalch equation to determine the pK_a.

S2.8 Determination of molar extinction coefficients

The UV-vis spectra of the iridium complexes **1-6** were recorded at 3 different concentrations from 800 nm to 200 nm, and the concentrations were subsequently determined by ICP-OES protocol. Linear plots of absorbance versus concentrations for each complex gave the molar extinction coefficient as the gradient, according to the Beer-Lambert law.

S2.9 Determination of the hydrolysis kinetics for complex 9

HPLC analysis with the detection wavelength at 254 nm relevant to 610 nm was conducted for complex **9** (50 μM) dissolved in MeOH/H_2O (5/95, v/v) and incubated at 310 K, at different time interval over 24 h. The normalized peak integral of complex **9** was calculated based on the aqua complexes. The curve of the integral versus time (min) was were plotted and fitted to the pseudo first-order kinetics to determine the hydrolysis rate and half-life time.

S2.10 Cyclic voltammetry

All the electrochemical measurements used a three-electrode configuration: the reference electrode was Ag/$AgNO_3$ (0.1 M in acetonitrile); the working electrode was a polished platinum disc and the counter electrode was a large surface area platinum wire. The electrolyte solution was 0.1 M $[Bu_4N]PF_6$ in HPLC grade acetonitrile solution. The concentration of the metal in all the samples, including the ferrocene reference, was 1 mM in 5 ml electrolyte which had been scanned as blank in advance after bubbling with N_2 at least for 15 min. The scan rate was 100 mV/s. Between each sample interval, all the electrodes were washed with acetone three times and dried under an air flow.

S2.11 High-performance Liquid Chromatography (HPLC) and Liquid Chromatography–Mass Spectrometry (LC-MS)

Reverse phase HPLC was performed on a HP 1100 Series HPLC System (Agilent) using a ZORBAX Eclipse Plus C-18 column (250 x 4.6 mm, 5 μm).

Eluents: A-water (0.1% v/v TFA), B-acetonitrile (0.1% v/v TFA). Solvent gradient method: 0-30 min, 10%-80% B; 30-40 min, 80% B; 40-41min, 80%-10% B; 40-55min, 10% B. A flow rate of 1 mL min^{-1} was used with detection wavelength 254 nm. LC-MS was carried out on a Bruker Amazon X connected online to an Agilent 1260 HPLC with detection wavelength 254 nm. The mobile phase and solvent gradient method were the same as for HPLC.

S2.12 Catalysis of GSH oxidation

The catalysis of GSH oxidation to GSSG by iodido iridium complexes **1**, **3**, **7** and **8**, free ligand phenol-azopyridine (HO-azpy), and the Ir-SG complexes **7-SG** and **3-SG** was studied by ^{1}H NMR spectroscopy. The tested compound and GSH were in 5% d_6-acetone and 95% phosphate buffer D_2O (v/v, 30 mM, pH* 7.4) in a sealed vial with a final concentration ratio of [Ir]/[GSH] = [100 μM]/[10 mM]. The dissolved oxygen (DO) concentration in the starting mixture was determined to be 4.33 ± 0.05 mg/L using a FiveGo™ F4 DO meter. Oxygen is involved in the catalytic cycle (Figure 12 in the main text). After 24 h at 310 K, the reaction solution was placed in a 5 mm NMR tube for recording the ^{1}H NMR spectrum (128 scans, 7 min). The catalytic activity of complex **7** under oxygen-depleted conditions was investigated by bubbling N_2 gas (99.998 % purity) into the reaction solution in the NMR tube through a needle vent hole for 5 min. Then the NMR tube was sealed with a cap and parafilm during the reaction.

The turnover number (TON) for the reactions was calculated as follows:

$$TON = \frac{I_{GSSG}}{I_{GSH} + I_{GSSG}} \frac{[GSH]_0}{[Ir]}$$

where I_{GSSG}/I_{GSH} are the peak integrals for oxidized/reduced glutathione at δ 3.30-3.36/2.90-3.03 ppm, respectively, and $[GSH]_0$ is the initial concentration of GSH. The TON is the average of three independent experiments with similar results. The TONs were determined to be 29 ± 2 (**1**), 18 ± 1 (**3**), 100 ± 4 (**7**), 100 ± 2 (**8**), 9 ± 1 (HO-azpy), 92 ± 1 (**7-SG**) and 18 ± 3 (**3-SG**).

S2.13 EPR experiments and simulation

All EPR experiments were performed on a Bruker EMX spectrometer operating at X-band with a cylindrical cavity (ER 4122 SHQE). Unless otherwise stated, experimental parameters were as follows: modulation amplitude 0.1 mT; conversion time and time constant 40.96 ms; number of scans 20. In spin-trapping experiments, the microwave power was 20 mW at ca. 9.8 GHz. GSH (40 mM) solution was prepared in a 0.1 M phosphate buffer at pH 7.8. Adventitious metals were removed from the phosphate buffer using Chelex 100 sodium form resin (50-100 mesh). Complex **7** was mixed with GSH stock solution to give 1 mM **7** and 20 mM GSH. Samples (ca. 100 µL) were transferred using a plastic syringe with metal needle to a standard quality quartz tube with an inner diameter of 1.0 mm and an outer diameter of 2.0 mm (Wilmad LabGlass). Data were processed by Matlab R2016b with Easyspin 5.1.12.

S2.14 Partition coefficient determination

The lipophilicity of complexes **3** and **7** is compared by determining their octanol-phosphate buffer (2 mM, pH 7.4) partition coefficients using the "shake-tube" method.[4] On account of the deprotonation of the phenolic group at physiological pH, which would affect the partition of the complex, phosphate buffer (pH 7.4) was used to mimic the physiological environment. 1-Octanol saturated 2 mM phosphate buffer and 2 mM phosphate buffer-saturated 1-octanol were prepared by stirring the mixtures of 100 mL octanol and 100 ml 2 mM phosphate buffer (pH 7.4, made with Milli-Q water) for 24 h and separated in a separating funnel after 3-4 h equilibration. 3.0-4.0 mg of iridium complexes were dissolved in 10 mL octanol-saturated 2 mM phosphate buffer by shaking the suspension solution on an IKA Vibrax VXC basic shaker for 5 h at the speed of 1000 r/min to give saturated solutions. Then the mixture was filtered using a hydrophobic filter. A 2 mL aliquot of the final filtrate was added into 2 mM phosphate buffer-saturated octanol (2 mL) in triplicate, and the two-layer solution was shaken on an IKA Vibrax VXC basic shaker for 15 h at a speed of 1000 r/min and

subsequently, equilibrated for 4.5 h. Aqueous samples before and after partitioning in octanol were diluted in the ratio of 1: 100 and 1:10 v/v, respectively, with ca. 3.6 % HNO_3 to the appropriate range for ICP-MS analysis. The iridium concentration was calibrated using aqueous standards in ca. 3.6 % HNO_3 (0-1000 ppb). These procedures were carried out at ambient temperature (ca. 298 K). Log P values for iridium complexes were calculated from log $P_{o/w}$ = log($[Ir]_{oct}/[Ir]_{aq}$).[5]

S2.15 Inductively Coupled Plasma-Optical Emission Spectroscopy (ICP-OES)

ICP-OES analyses were carried out on a PerkinElmer Optima 5300 DV series Optical Emission Spectrophotometer. The iridium plasma standard was diluted with 3.6% HNO_3 in type-1 Milli-Q water to give freshly-prepared calibrants at concentrations of 700, 600, 500, 400, 300, 200, 100, 50, and 0 ppb, which were adjusted to match the sample matrix by standard addition of sodium chloride. Total dissolved solids did not exceed 0.2% w/v. Data were acquired and processed using WinLab32 V3.4.1 for Windows.

S2.16 Inductively Coupled Plasma-Mass Spectrometry (ICP-MS)

The whole cell pellet was digested in 200 μL 72% nitric acid at 353 K overnight. Digested samples were then diluted in the ratio of 1:20 (v/v) to give a final concentration of 3.6% nitric acid in 4 mL volume and analyzed using an Agilent 7500 series or Agilent 7900 series instrument with an internal standard of ^{166}Er (50 ppb) in both no-gas and He-gas mode. Data were acquired and processed using Agilent ChemStation for Windows (7500 series ICP-MS) or Agilent Mass Hunter 4.3 for Windows (7900 series ICP-MS). Biological triplicates were produced for each sample and statistical significance was calculated (Welch's unpaired t-test).

S2.17 Cell culture

Cancer cells were grown in Roswell Park Memorial Institute medium (RPMI-1640) supplemented with 10% (v/v) of fetal calf serum, 1% (v/v) of 2 mM glutamine, and 1% (v/v) penicillin/streptomycin. Cells were grown as adherent monolayers at 310 K in a 5% CO_2 humidified atmosphere and passaged at ca. 80% confluency using trypsin-EDTA. A2780cis cisplatin-resistant cells were exposed to cisplatin for 24 h upon reaching 80-90% confluence. After this time, the cells were washed with PBS and supplied with fresh medium.

S2.18 *In vitro* cell growth inhibition

Briefly, 5000 cells in 150 μL cell culture medium were seeded per well in 96-well plates and pre-incubated at 310 K for 48 h in a 5% CO_2 humidified atmosphere before adding different concentrations of test compounds. Stock solutions of the tested compounds (ca. 0.1 mM) were prepared in DMSO (0.5%, v/v)/cell culture medium and the concentration of each compound in the stock solution was determined by ICP-OES based on metal iridium. The supernatants were removed by suction and cells were treated with six concentrations of the tested compounds using 200 μL per well for 24 h at 310 K in a 5% CO_2 humidified atmosphere. After this, supernatants were removed by suction and each well was washed with 100 μL PBS. The cells were allowed a further 72 h recovery in compound-free fresh medium at 310 K in a 5% CO_2 humidified atmosphere. The SRB assay was then used to determine cell viability. Absorbance measurements of the solubilized dye (on a BioRad iMark microplate reader using a 470 nm filter) allowed the determination of viable treated cells compared to untreated controls. IC_{50} values were determined as duplicates of triplicates in two independent sets of experiments and their standard deviations were calculated.

S2.19 Toxicity in zebrafish

Zebrafish experiments using Singapore wild-type zebrafish embryos were

carried out at School of Life Sciences (University of Warwick). Embryos were collected and transferred to petri dishes. Zebrafish embryos were harvested and seeded in 24-well plates at one embryo/well. A serial dilution of six concentrations of the compound to be tested was added to 20 of these wells with the other 4 wells as negative controls. DMSO at an identical percentage to that present in the tested compound solutions was used as the solvent control and 3,4-dichloroaniline used as the positive control. Embryos incubated at 301.5 K for 96 h, after which percentage of zebrafish survival was assessed by embryo coagulation, visible heartbeat, tail detachment and somite formation. Experiments were carried out in duplicate. Complex concentrations were corrected by ICP-OES and percentage of survival was plotted against complex concentration and fitted as Boltzmann sigmoidal curves to determine the LC_{50} values and standard deviations. Maintenance of animals was carried out in accordance with ASPA1986 and approved by the University Ethical Review Committee. Zebrafish were housed in 3.5 L tanks and fed 2 – 4 times a day. Fish were mated once a week starting at two pairs per 1 L breeding tank.

S2.20 Ir accumulation in cancer cells

The accumulation of iridium in A549 human lung carcinoma cells was determined by ICP-MS. $1x10^6$ cells were seeded onto a 100 mm Petri dish and incubated in compound-free media for 24 h at 310 K in a 5% CO_2 humidified atmosphere. For a further 24 h the cells were incubated in media containing a range (0.25×, 0.5×, 1× and 2× IC_{50}) of compound concentrations (determined by ICP-OES). Following this, cells were counted and cell pellets were collected and digested as ICP-MS protocol.

S2.21 ROS determination

Flow cytometry analysis of ROS/superoxide generation in A549 cells caused by exposure to complex **3** and **7** was carried out using the Total ROS/Superoxide detection kit (Enzo-Life Sciences) according to the

instructions. A total of 5 × 10^5 A549 cells per well were seeded in a six-well plate. Cells were preincubated in compound-free media at 310 K for 24 h in a 5% CO_2 humidified atmosphere, and then compounds were added to triplicates at 2x IC_{50} concentration. After 24 h of compound exposure, supernatants were removed by aspiration and cells were washed with PBS and harvested. Staining was achieved by resuspending the cell pellets in buffer containing the orange/green fluorescent reagents. Cells were analyzed in a Becton Dickinson FACScan flow cytometer using FL1 channel Ex/Em 490/525 nm for the oxidative stress and FL2 channel Ex/Em 550/620 nm for superoxide detection. Positive controls were obtained by exposure of cells to pyocyanin for 30 min. Data were processed using Flowjo software. At all times, samples were kept under dark conditions to avoid light-induced ROS production. Welch's t-tests were carried out to establish statistical significance of the variations.

S2.22 Computational details

All calculations were performed in the framework of Density Functional Theory employing the hybrid Becke three-parameter exchange functional[6] and the Lee–Yang–Parr correlation functional, B3LYP.[7] To carry out such calculations the Gaussian 09 suite of program[8] was used. Grimme approach was adopted to include dispersion corrections for nonbonding interaction using atom pair-wise additive schemes,[9] denoted as DFT-D3 method.

The LANL2DZ effective core potential was used for the Ir atom,[10] along with the split valence basis set. The standard triple-ζ quality 6-311+G** basis sets of Pople and coworkers were used for the atoms directly participating in the process, whereas in order to reduce the computational effort, the 6-31G* basis sets were employed for peripheral atoms.

In order to establish both the nature of intercepted stationary points as minima and transition states and calculate zero-point energy (ZPE) and Gibbs free energy corrections, vibrational frequencies at the same level of theory were calculated. The intercepted transition states are first order saddle points on a

potential energy surface (PES) and their vibrational spectrum is characterized by one imaginary frequency, corresponding to a negative force constant, which means that in one direction, in the nuclear configuration space, the energy has a maximum, while in all the other directions the energy has a minimum. Furthermore, IRC (intrinsic reaction coordinate) analysis was employed to carefully check transition state structures to be properly connected to the correct minima.[11,12]

The effects due to the presence of the solvent were included using the Tomasi's implicit Polarizable Continuum Model (PCM)[13-15] as implemented in Gaussian09 and the UFF set of radii was adopted to build-up the cavity in which the solute molecules are accommodated. Solvation Gibbs free energies were calculated performing single-point calculations for all *in vacuum* stationary points structures in implicit water (ε=78.4) at the same level of theory. Enthalpies and Gibbs free energies were obtained using standard statistical procedures[16] at 298 K and 1 atm from total energies, including zero-point, thermal and solvent corrections. As the free energy corrections in the Gaussian's default standard state corresponds to an ideal gas at a standard pressure of 1 atm, the computed free energies were converted to yield Gibbs energies with a solution phase standard state of 1 mol L^{-1} for all the species except water solvent.[17] For water molecules a standard state of 55.5 M has been used. That is, to the free energy of each species, as computed in Gaussian, a free energy correction term equal to RT ln($V_{molar\ gas}/V_{molar\ solution}$), (R = gas constant, T = absolute temperature) was added, where $V_{molar\ gas}$ is the volume occupied by one mole of ideal gas at the considered temperature, and $V_{molar\ solution}$ is the volume occupied by one mole of species in a standard solution of concentration 1 mol L^{-1} .

S3 Synthesis and characterization

S3.1 Synthesis of ligands and iridium precursor

Phenyl-azopyridine ligands with different substituents were synthesized and characterized according to a reported procedure.[18]

The $[(\eta^5\text{-Cp}^*)\text{Ir}(\mu\text{-Cl})\text{Cl}]_2$ and $[(\eta^5\text{-Cp}^{xph})\text{Ir}(\mu\text{-Cl})\text{Cl}]_2$ precursors were also obtained by reported methods.[19]

The iridium(III) dimers $[(\eta^5\text{-Cp}^X)\text{Ir}(\mu\text{-I})\text{I}]_2$ (Cp^X = Cp^* or Cp^{xph}) were obtained following a general procedure: the $[(\eta^5\text{-Cp}^X)\text{Ir}(\mu\text{-Cl})\text{Cl}]_2$ (Cp^X = Cp^* or Cp^{xph}) dimer was dissolved in 20 mL MeOH and 40 mL H_2O under reflux at 358 K for 1 h. KI solid (100 mol equiv.) was added to the solution and stirred on an ice bath for 3 h. The reaction mixture was stored at 280 K overnight. The final precipitate was filtered on a Buchner funnel, then dissolved in DCM, and dried with anhydrous $MgSO_4$. The red solid product was collected by removing the DCM on a rotary evaporator.

$[(\eta^5\text{-Cp}^*)\text{Ir}(\mu\text{-I})\text{I}]_2$: yield 91.8%, ^{1}H NMR (400 MHz, $CDCl_3$): δ 1.83 (15H, s).

$[(\eta^5\text{-Cp}^{xph})\text{Ir}(\mu\text{-I})\text{I}]_2$: yield 72%, ^{1}H NMR (400 MHz, $CDCl_3$): δ 7.45-7.43 (2H, m), 7.34-7.32 (3H, m), 1.97 (6H, s), 1.94 (6H, s).

S3.2 Synthesis of iridium complexes

All the iridium iodido complexes were synthesized following a general procedure: the ligand (0.10 mmol, 2.0 mol equiv.) was added to 10 mL dichloromethane solution of $[(\eta^5\text{-Cp}^X)\text{Ir}(\mu\text{-I})\text{I}]_2$ (Cp^X = Cp^* or Cp^{xph}) (0.05 mmol, 1.0 mol equiv.). The mixture immediately turned to dark red and was stirred at ambient temperature for 24 h. The solvent was removed on a rotary evaporator, then a minimum volume of methanol was added to dissolve the crude product, and excess NH_4PF_6 (0.50 mmol, 10.0 mol equiv.) solid was added. Precipitation occurred after storage at ca. 253 K for 2-3 days. The final solid product was collected by filtration, washed by diethyl ether, and dried under vacuum.

Note: the carbon atoms are labelled in the structures below.

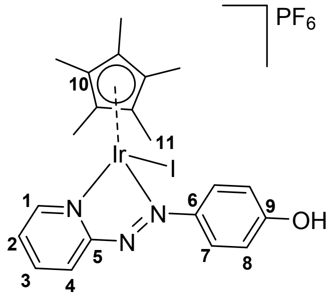

[(η^5-Cp*)Ir(HO-azpy)I]PF$_6$ (**1**): black solid, yield 68%. ^{1}H NMR (500 MHz, CD_3OD): δ 8.94 (1H, d, J = 5.6 Hz, H̲C1), 8.81 (1H, d, J = 8.0 Hz, H̲C4), 8.31 (1H, t, J = 7.8 Hz, H̲C3), 8.03 (2H, d, J = 8.9 Hz, H̲C7), 7.82 (1H, t, J_1 = 6.9 Hz, J_2 = 6.4 Hz, H̲C2), 6.90 (2H, d, J = 8.9 Hz, H̲C8), 1.74 (15H, s, H̲C11). ^{13}C NMR (125 MHz, CD_3OD): δ 167.47 (C5), 166.65 (C6), 152.80 (C1), 148.98 (C9), 143.40 (C3), 130.07 (C7), 129.17 (C2), 127.94 (C4), 117.12 (C8), 96.26 (C10), 9.56 (C11). ESI-MS: calcd for [(η^5-Cp*)Ir(HO-azpy)I]$^+$ *m/z* 654.0, found 654.1; elemental analysis calcd for $C_{21}H_{24}F_6IIrN_3OP$: C, 31.59; H, 3.03; N, 5.26; found: C, 30.98; H, 2.86; N, 5.26.

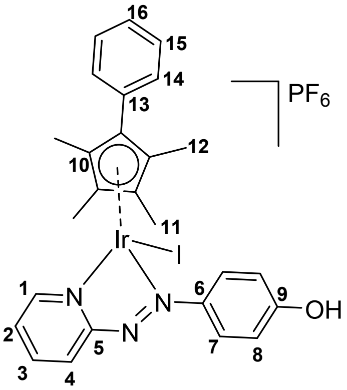

[(η^5-Cp^{xph})Ir(HO-azpy)I]PF$_6$ (**2**)**:** dark blue solid, yield 85%. ^{1}H NMR (500 MHz, CD_3OD): δ 8.83 (1H, d, J = 8.0 Hz, H̲C4), 8.72 (1H, d, J = 5.6 Hz, H̲C1), 8.29 (1H, t, J = 7.8 Hz, H̲C3), 8.04 (2H, d, J = 9.0 Hz, H̲C7), 7.71 (1H, t, J = 7.0 Hz, H̲C2), 7.46 (1H, t, J = 7.4 Hz, H̲C16), 7.40 (2H, t, J_1 = 7.7 Hz, J_2 = 7.2 Hz, H̲C15), 7.20 (2H, d, J = 7.2 Hz, H̲C14), 6.86 (2H, d, J = 9.0 Hz, H̲C8), 2.04 (3H, s, H̲C11), 1.96 (3H, s, H̲C11), 1.89 (3H, s, H̲C12), 1.46 (3H, s, H̲C12). ^{13}C NMR

(125 MHz, CD_3OD): δ 167.65 (C5), 167.61 (C6), 152.32 (C1), 148.81 (C9), 143.63 (C3), 130.96 (C14), 130.64 (C16), 130.43 (C15), 130.43 (C7), 129.34 (C13), 128.97 (C2), 128.07 (C4), 117.37 (C8), 103.65 (C10), 102.53 (C10), 94.34 (C10), 93.26 (C10), 92.64 (C10), 10.80 (C11), 10.70 (C11), 10.09 (C12), 9.78 (C12). ESI-MS: calcd for $[(\eta^5\text{-Cp}^{xph})\text{Ir(HO-azpy)I}]^+$ *m/z* 716.0, found 716.0; elemental analysis calcd for $C_{26}H_{26}F_6IIrN_3OP\cdot H_2O$: C, 35.54; H, 3.21; N, 4.78; found: C, 35.70; H, 3.17; N, 4.63.

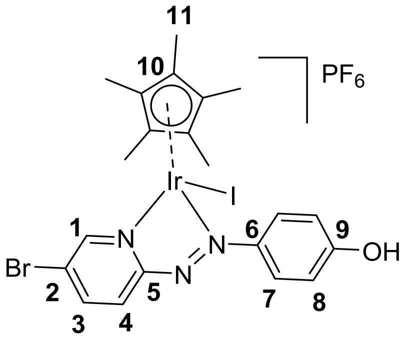

$[(\eta^5\text{-Cp}^*)\text{Ir(HO-azpy-Br)I}]PF_6$ (3): dark blue solid, yield 85%. ^1H NMR (500 MHz, CD_3OD): δ 8.90 (1H, d, *J* = 1.8 Hz, H̲C1), 8.59 (1H, d, *J* = 8.6 Hz, H̲C3), 8.45 (1H, d, *J* = 8.6 Hz, H̲C4), 8.06 (2H, d, *J* = 9.0 Hz, H̲C7), 6.90 (2H, d, *J* = 9.0 Hz, H̲C8), 1.76 (15H, s, H̲C11). ^{13}C NMR (125 MHz, CD_3OD): δ 166.48 (C5), 152.53 (C1), 148.63 (C9), 146.03 (C4), 130.78 (C7), 127.07 (C3), 123.36 (C6), 121.41 (C2), 118.14 (C8), 96.23 (C10), 9.54 (C11). ESI-MS: calcd for $[(\eta^5\text{-Cp}^*)\text{Ir(HO-azpy-Br)I}]^+$ *m/z* 731.9, found 732.0; elemental analysis calcd for $C_{21}H_{23}BrF_6IIrN_3OP\cdot 2H_2O$: C, 27.61; H, 2.98; N, 4.60; found: C, 27.48; H, 2.79; N, 4.67.

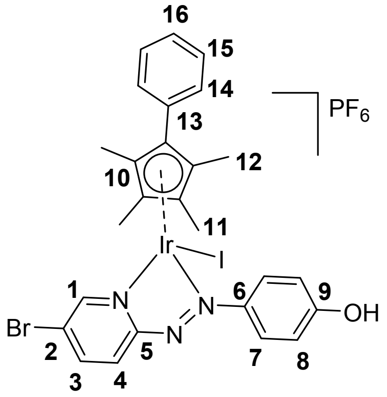

[(η^5-Cp^{xph})Ir(HO-azpy-Br)I]PF$_6$ (**4**): dark blue solid, yield 60%. ^{1}H NMR (500 MHz, CD_3OD): δ 8.72 (1H, d, *J* = 8.6 Hz, H̲C4), 8.61 (1H, d, *J* = 1.8 Hz, H̲C1), 8.48 (1H, d, *J* = 8.6 Hz, H̲C3), 8.05 (2H, d, *J* = 9.1 Hz, H̲C7), 7.54-7.47 (3H, m, H̲C16, H̲C15), 7.33 (2H, d, *J* = 7.1 Hz, H̲C14), 6.89 (2H, d, *J* = 9.0 Hz, H̲C8), 2.03 (3H, s, H̲C11), 2.02 (3H, s, H̲C11), 1.83 (3H, s, H̲C12), 1.49 (3H, s, H̲C12). ^{13}C NMR (125 MHz, CD_3OD): δ 166.47 (C5), 151.92 (C1), 148.93 (C9), 146.38 (C3), 131.10 (C7), 130.91 (C15), 130.64 (C16), 130.59 (C14), 128.88 (C13), 128.21 (C4), 124.24 (C6), 121.91 (C2), 117.51 (C8), 105.27 (C10), 101.53 (C10), 96.46 (C10), 92.35 (C10), 92.15 (C10), 11.35 (C11), 10.56 (C11), 10.06 (C12), 9.42 (C12). ESI-MS: calcd for [(η^5-Cp^{xph})Ir(HO-azpy-Br)I]$^+$ *m/z* 793.9, found 794.0; elemental analysis calcd for $C_{26}H_{25}BrF_6IIrN_3OP\cdot H_2O$: C, 32.61; H, 2.84; N, 4.39; found: C, 32.26; H, 2.80; N, 4.40.

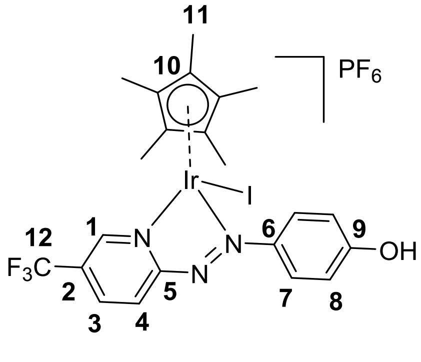

[(η^5-Cp*)Ir(HO-azpy-CF$_3$)I]PF$_6$ (**5**): black solid, yield 58%. ^{1}H NMR (500 MHz, CD_3OD): δ 9.03 (1H, brs, H̲C1), 8.90 (1H, d, *J* = 8.5 Hz, H̲C4), 8.58 (1H, d, *J* = 8.5 Hz, H̲C3), 8.11 (2H, d, *J* = 9.0 Hz, H̲C7), 6.97 (2H, d, *J* = 9.0 Hz, H̲C8), 1.77 (15H, s, H̲C11). ^{19}F NMR (376 MHz, CD_3OD): δ 63.53 (F̲$_3$C12), 73.95 (F̲$_6$P),

75.83 ($\underline{F}_6$P). ^{13}C NMR (125 MHz, CD_3OD): δ 169.37 (C5), 149.45 (C9), 149.30 (C1), 140.65 (C3), 131.14 (C7), 129.35 (C12), 129.50 (C12), 129.23 (C12), 128.95 (C12), 126.86 (C4), 124.70 (C6), 122.55 (C2), 117.96 (C8), 96.96 (C10), 9.58 (C11). ESI-MS: calcd for [(η^5-Cp*)Ir(HO-azpy-CF_3)I]$^+$ *m/z* 722.1, found 722.0; elemental analysis calcd for $C_{22}H_{23}F_9IIrN_3OP{\cdot}H_2O$: C, 29.87; H, 2.85; N, 4.75; found: C, 30.00; H, 2.80; N, 4.69.

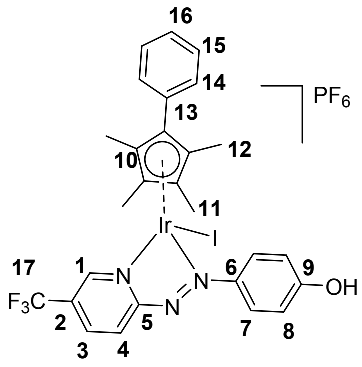

[(η^5-Cp^{xph})Ir(HO-azpy-CF_3)I]PF_6 (6): dark green solid, yield 65%. ^{1}H NMR (500 MHz, CD_3OD): δ 8.48 (1H, s, $\underline{H}$C1), 8.40 (1H, d, *J* = 8.9 Hz, $\underline{H}$C4), 8.26 (1H, d, *J* = 8.5 Hz, $\underline{H}$C3), 8.16 (2H, d, *J* = 9.3 Hz, $\underline{H}$C7), 7.52 (1H, t, *J* = 7.2 Hz, $\underline{H}$C16), 7.47 (2H, t, J_1 = 7.6 Hz, J_2 = 7.1 Hz, $\underline{H}$C15), 7.41 (2H, d, *J* = 7.2 Hz, $\underline{H}$C14), 6.55 (2H, d, *J* = 9.1 Hz, $\underline{H}$C8), 2.01 (3H, s, $\underline{H}$C11), 1.99 (3H, s, $\underline{H}$C11), 1.81 (3H, s, $\underline{H}$C12), 1.55 (3H, s, $\underline{H}$C12). ^{13}C NMR (125 MHz, CD_3OD): δ 170.09 (C5), 149.19 (C9), 147.95 (C1), 139.19 (C3), 133.19 (C7), 131.18 (C14), 130.69 (C15), 130.56 (C16), 129.99 (C17),129.58 (C17), 129.27 (C17), 128.85 (C17), 129.30 (C13), 124.92 (C6), 122.71 (C8), 121.99 (C4), 121.41 (C2), 104.13 (C10), 100.14 (C10), 95.33 (C10), 91.18 (C10), 90.36 (C10), 11.44 (C11), 10.59 (C11), 10.22 (C12), 9.29 (C12). ESI-MS: calcd for [(η^5-Cp^{xph})Ir(HO-azpy-CF_3)I]$^+$ *m/z* 784.0, found 784.0; elemental analysis calcd for $C_{27}H_{25}F_9IIrN_3OP{\cdot}0.1H_2O$: C, 34.86; H, 2.73; N, 4.52; found: C, 35.19; H, 2.78; N, 4.57.

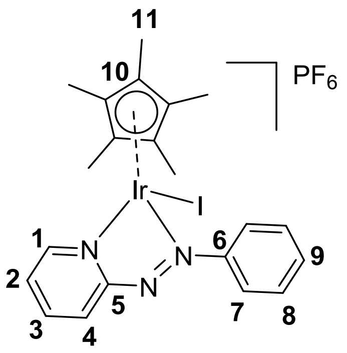

[(η^5-Cp*)Ir(azpy)I]PF$_6$ (7): black solid, yield 95%. ^{1}H NMR (CD$_3$OD, 500 MHz): δ 9.02 (1H, d, J = 6.6 Hz, H̲C1), 9.00 (1H, d, J = 7.8 Hz, H̲C4), 8.39 (1H, t, J = 7.7 Hz, H̲C3), 8.04 (2H, d, J = 7.6 Hz, H̲C7), 7.91 (1H, t, J = 7.7 Hz, H̲C2), 7.77 (1H, t, J = 7.3 Hz, H̲C9), 7.70 (2H, t, J = 7.7 Hz, H̲C8), 1.74 (15H, s, H̲C11). ^{13}C NMR (125 MHz, CD$_3$OD): δ 166.30 (C5), 155.32 (C6), 153.04 (C1), 143.60 (C3), 135.30 (C9), 130.64 (C2), 130.64 (C8), 129.69 (C4), 126.68 (C7), 97.07 (C10), 9.61 (C11). ESI-MS: calcd for [(η^5-Cp*)Ir(azpy)I]$^+$ m/z 638.0, found 638.1; elemental analysis calcd for $C_{21}H_{24}F_6IIrN_3P\cdot H_2O$: C, 31.51; H, 3.27; N, 5.25; found: C, 31.36; H, 3.01; N, 5.41.

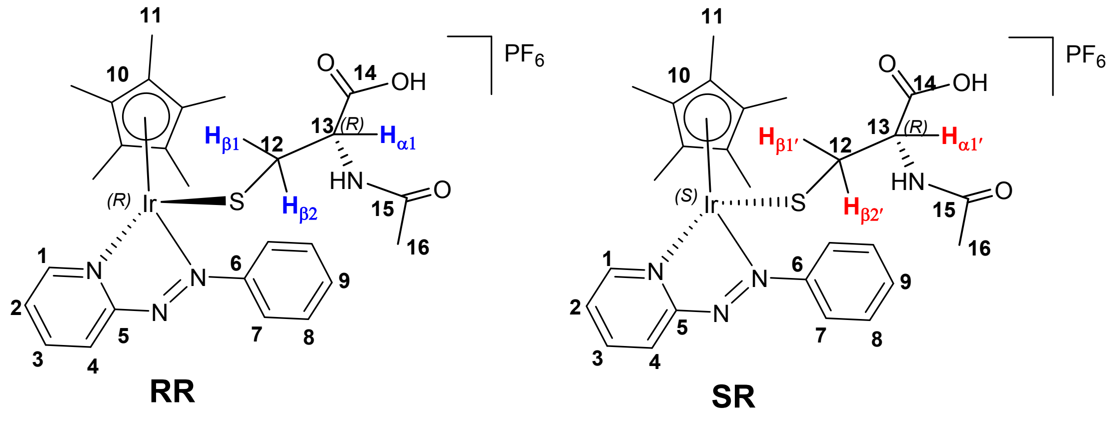

[(η^5-Cp*)Ir(azpy)(NAC)]PF$_6$ (7-NAC): dark brown solid, yield 97%. ^{1}H NMR (500 MHz, 70% D$_2$O-30% CD$_3$OD set δ (CD$_3$OD) = 3.26 ppm): δ 8.89 (1H, d, J = 8.2 Hz, H̲C4,), 8.78 (1H, d, J = 5.7 Hz, H̲C1), 8.31 (1H, t, J = 8.0 Hz, H̲C3), 7.97 (1H, m, H̲C2), 7.73 (2H, t, J = 7.7 Hz, H̲C7), 7.67-7.69 (3H, m, H̲C8, H̲C9), 3.86-3.84 (0.5H, q, J ($H_{\alpha1}$-$H_{\beta1}$) = 7.3 Hz, J ($H_{\alpha1}$-$H_{\beta2}$) = 4.3 Hz, $\underline{H_{\alpha1}}$C13), 1.80-1.84 (0.5 H, q, J ($H_{\beta1}$-$H_{\alpha1}$) = 7.3 Hz, J ($H_{\beta1}$-$H_{\beta2}$) = 12.0 Hz, $\underline{H_{\beta1}}$C12), 1.77-1.75

(0.5H, q, J ($H_{\beta 2}$-$H_{\alpha 1}$) = 4.3, J ($H_{\beta 2}$-$H_{\beta 1}$) = 12.0 Hz, $H_{\beta 2}$C12), 3.77-3.75 (0.5H, q, J ($H_{\alpha 1'}$-$H_{\beta 1'}$) = 6.5 Hz, J ($H_{\alpha 1'}$-$H_{\beta 2'}$) = 4.3 Hz, $H_{\alpha 1'}$C13), 2.08-2.04 (0.5H, q, J ($H_{\beta 1'}$-$H_{\alpha 1'}$) = 6.5 Hz, J ($H_{\beta 1'}$-$H_{\beta 2'}$) = 12.0 Hz, $H_{\beta 1'}$C12), 1.74-1.72 (0.5H, q, J ($H_{\beta 2'}$- $H_{\alpha 1'}$) = 4.3 Hz, J ($H_{\beta 2'}$-$H_{\beta 1'}$) = 12.0 Hz, $H_{\beta 2'}$C12),1.86 (1.5H, s, HC16), 1.73(1.5H, s, HC16), 1.54 (15H, s, HC11). ^{13}C NMR (125 MHz, 70% D_2O-30% CD_3OD set δ (CD_3OD) = 49.00 ppm): δ 176.93 (C14), 176.81 (C14), 173.55 (C15), 173.27 (C15), 165.4 (C5), 166.96 (C5), 155.42 (C6), 150.75 (C1), 150.70 (C1), 142.98 (C3), 142.88 (C3), 134.65 (C9), 134.72 (C9), 130.61 (C8),130.55 (C8), 129.51 (C2), 129.47 (C2), 128.90 (C4), 128.85 (C4), 125.32 (C7),125.28 (C7), 98.07 (C10), 97.95 (C10), 56.83 (C13), 57.26 (C13), 29.23 (C12), 29.15 (C12), 22.93 (C16), 22.83 (C16), 8.37 (C11), 8.33 (C11). ESI-MS: calcd for [(η^5-Cp*)(Ir-NAC)(azpy)]$^+$ *m/z* 673.2, found 673.3; calcd for [(η^5-Cp*)Ir(azpy)NAC]-H+Na]$^+$ *m/z* 695.2, found 695.3 elemental analysis calcd for $C_{26}H_{32}F_6IrN_4O_3PS$: C, 38.19; H, 3.94; N, 6.85; found: C, 38.25; H, 3.98; N, 6.54.

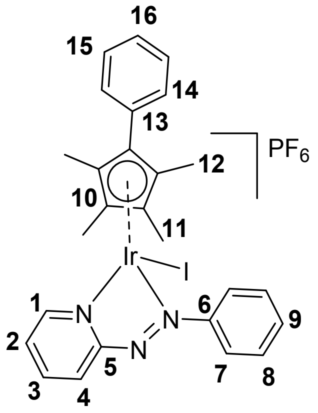

[(η^5-Cp^{xph})Ir(azpy)I]PF_6 (8): black solid, yield 83%. ^{1}H NMR (500 MHz, CD_3OD): δ 9.07 (1H, d, J = 7.9 Hz, HC4), 8.80 (1H, d, J = 5.5 Hz, HC1), 8.39 (1H, t, J = 7.7 Hz, HC3), 8.03 (2H, d, J = 7.7 Hz, HC7), 7.84 (1H, t, J = 6.1 Hz, HC2), 7.72 (1H, t, J = 7.5 Hz, HC9), 7.61 (2H, t, J = 8.0 Hz, HC8), 7.47 (1H, t, J = 7.4 Hz, HC16), 7.42 (2H, t, J = 7.5 Hz, HC15), 7.20 (2H, d, J = 7.2 Hz, HC14), 2.01 (3H, s, HC11), 1.96 (3H, s, HC11), 1.91 (3H, s, HC12), 1.38 (3H, s, HC12). ^{13}C NMR (125 MHz, CD_3OD): δ 167.37 (C5), 156.15 (C6), 152.58

(C1), 143.93 (C3), 135.47 (C9), 131.04 (C14), 130.81 (C15), 130.60 (C8), 130.56 (C16), 130.49 (C2), 130.22 (C4), 128.93 (C13), 126.75 (C7), 104.65 (C10), 102.47 (C10), 96.26 (C10), 94.12 (C10), 93.38 (C10), 10.96 (C11), 10.57 (C11), 9.84 (C12), 9.75 (C12). ESI-MS: calcd for $[(\eta^5\text{-Cp}^{xph})\text{Ir(azpy)I}]^+$ *m/z* 700.0, found 700.1; elemental analysis calcd for $C_{26}H_{25}F_6IIrN_3P{\cdot}H_2O$: C, 36.24; H, 3.16; N, 4.88; found: C, 37.02; H, 2.99; N, 4.90.

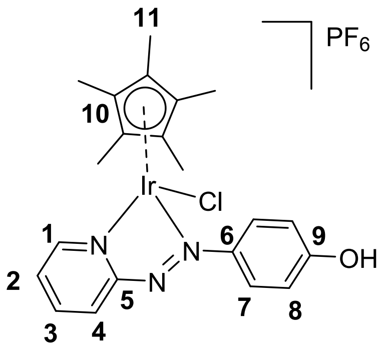

$[(\eta^5\text{-Cp}^*)\text{Ir(HO-azpy)Cl}]\text{PF}_6$ (9): black solid, yield 73%. ^{1}H NMR (400 MHz, CD_3CN): δ 8.77 (1H, d, *J* = 5.5 Hz, HC1), 8.66 (1H, d, *J* = 7.7 Hz, HC4), 8.31 (1H, t, *J*= 7.8 Hz, HC3), 7.95 (2H, d, *J* = 8.8 Hz, HC7), 7.87 (1H, t, *J* = 6.7 Hz, HC2), 7.28 (2H, d, *J* = 8.9 Hz, HC8), 1.57 (15H, s, HC11). ^{13}C NMR (125 MHz, CD_3CN): δ 166.27 (C5), 164.25 (C6), 150.67 (C1), 147.63 (C9), 143.35 (C3), 129.98 (C7), 128.17 (C2), 127.68 (C4), 116.80 (C8), 94.63 (C10), 8.54 (C11). ESI-MS: calcd for $[(\eta^5\text{-Cp}^*)\text{Ir(HO-azpy)Cl}]^+$ *m/z* 562.1, found 562.1; elemental analysis calcd for $C_{21}H_{24}F_6ClIrN_3OP$: C, 35.67; H, 3.42; N, 5.94; found: C, 35.67; H, 3.34; N, 5.90.

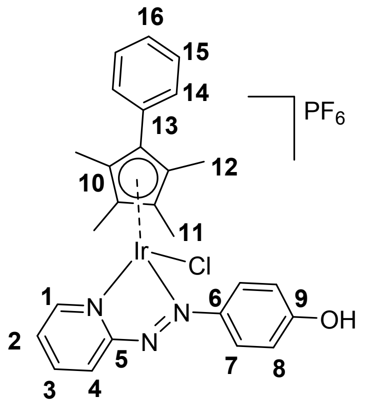

[(η^5-Cp^{xph})Ir(HO-azpy)Cl]PF$_6$ (10): black solid, yield 63%. ^{1}H NMR (400 MHz, CD_3CN): δ 8.50 (1H, d, *J* = 7.9 Hz, HC4), 8.26 (1H, d, *J* = 5.5 Hz, HC1), 8.15 (1H, t, *J* = 7.8 Hz, HC3), 7.92 (2H, d, *J* = 8.6 Hz, HC7), 7.56-7.46 (3H, m, HC2, HC16, HC15), 7.32 (2H, d, *J* = 7.3 Hz, HC14), 7.22 (2H, d, *J* = 8.4 Hz, HC8), 1.79 (3H, s, HC11), 1.67 (3H, s, HC11), 1.61 (3H, s, HC12), 1.43 (3H, s, HC12). ^{13}C NMR (125 MHz, CD_3CN): δ 166.36 (C5), 164.60 (C6), 150.47 (C1), 147.43 (C9), 143.50 (C3), 130.45 (C14), 130.08 (C16), 129.88 (C15), 128.83 (C13), 128.35 (C7), 127.99 (C2/C4),116.86 (C8), 104.79 (C10), 100.74 (C10), 94.24 (C10), 91.46 (10), 87.35 (C10), 9.74 (C11), 9.43 (C11), 9.00 (C12), 8.57 (C12). ESI-MS: calcd for [(η^5-Cp^{xph})Ir(HO-azpy)Cl]$^+$ *m/z* 624.1, found 624.0; elemental analysis calcd for $C_{26}H_{26}F_6ClIrN_3OP$: C, 40.60; H, 3.41; N, 5.46; found: C, 40.89; H, 3.31; N, 5.50.

Table S1. Crystallographic data for complexes **1′**·MeOH, **2**, **3**·MeOH, and **5**·MeOH.

**Parameters**	**1′**·MeOH	**[2-H$_{0.5}$]·[PF$_6$]$_{0.5}$**	**3**·MeOH	**5**·MeOH
Empirical formula	$C_{22}H_{28}I_2IrN_3O_2$	$C_{26}H_{25.5}F_3IIrN_3OP_{0.5}$	$C_{22}H_{27}BrF_6IIrN_3O_2P$	$C_{23}H_{27}F_9IIrN_3O_2P$
MW	812.47	787.58	909.44	898.54
T[K]	150(2)	150(2)	150(2)	296(2)
Crystal size [mm^3]	0.16 × 0.08 × 0.08	0.18 × 0.14 × 0.12	0.16 × 0.1 × 0.06	0.24 × 0.2 × 0.08
Crystal System	monoclinic	monoclinic	monoclinic	monoclinic
Space group	P2$_1$/n	P2/c	P2$_1$/n	P2$_1$/n
a [Å]	9.29339(12)	23.1650(4)	9.61097(8)	9.85870(10)
b [Å]	13.27724(15)	7.44609(9)	20.10144(16)	20.2807(2)
c [Å]	20.1881(3)	15.7060(2)	14.45419(15)	15.0835(2)
α [°]	90	90	90	90
β [°]	91.3644(12)	107.2675(16)	103.4265(10)	105.2450(10)
γ [°]	90	90	90	90
V [Å^3]	2490.31(6)	2587.00(7)	2716.14(4)	2490.31(6)
Z	4	4	4	4
ρ_{calcd} [g cm^{-3}]	2.167	2.022	2.224	2.051
μ [mm^{-1}]	7.863	6.431	21.338	18.514
F(000)	1520.0	1500.0	1720.0	1712.0
Radiation	MoKα (λ = 0.71073)	MoKα (λ = 0.71073)	CuKα (λ = 1.54184)	CuKα (λ = 1.54184)
θ range [°]	4.87 - 62.262	5.192- 66.2	7.674-146.66	7.476-155.982
Limiting indices	-13 ≤ h ≤ 13<br>-18 ≤ k ≤ 18<br>-28 ≤ l ≤ 28	-34 ≤ h ≤ 35<br>-11 ≤ k ≤ 11<br>-24 ≤ l ≤ 23	-11 ≤ h ≤ 11<br>-24 ≤ k ≤ 24<br>-17 ≤ l ≤ 15	-10 ≤ h ≤ 12<br>-24 ≤ k ≤ 25<br>-18 ≤ l ≤ 19
Collected reflns	64660	97034	29026	16591
Unique reflns	7479 [R_{int} = 0.0416]	9366 [R_{int} = 0.0514]	5384 [R_{int} = 0.0555]	6029 [R_{int} = 0.0469]
Data/restraints/parameters	7479/0/279	9366/0/334	5384/0/379	6029/112/390
Goodness of fit	1.093	1.050	1.121	1.052
R_1/wR_2[I>2σ(I)]	0.0208/ 0.0381	0.0231/0.0416	0.0293/ 0.0770	0.0507/0.1375
R_1/wR_2(all data)	0.0273/ 0.0398	0.0314/0.0439	0.0295/ 0.0772	0.0526/0.1404
Largest diff. peak/hole/e Å^{-3}	0.57/-0.90	0.81/-0.96	2.00/-1.81	1.92/-1.71

Table S2. Crystallographic data for complexes **7′**, **8′**, **9′**·MeOH and **10**.

**Parameters**	**7′**	**8′**	**[9′-H$_{0.5}$]·[Cl]$_{0.5}$**·MeOH	**10**
Empirical formula	$C_{21}H_{24}I_2IrN_3$	$C_{26}H_{26}I_2IrN_3$	$C_{22}H_{27.5}Cl_{1.5}IrN_3O_2$	$C_{26}H_{26}ClF_6IrN_3OP$
MW	764.43	826.50	611.34	769.12
T[K]	150(2)	150(2)	150(2)	150(2)
crystal size [mm^3]	0.18 × 0.1 × 0.02	0.2 × 0.08 × 0.02	0.5 × 0.35 × 0.28	0.35 × 0.16 × 0.06
Crystal System	monoclinic	monoclinic	monoclinic	triclinic
Space group	C2/c	P2$_1$/c	P2$_1$/c	P-1
a [Å]	31.7681(4)	9.19420(10)	8.93939(15)	8.71914(18)
b [Å]	8.70230(10)	8.21580(10)	18.6768(3)	9.9018(2)
c [Å]	16.6407(2)	33.7933(3)	13.4520(2)	15.7288(3)
α [°]	90	90	90	89.3248(16)
β [°]	94.9350(10)	95.7830(10)	94.0660(14)	78.0376(16)
γ [°]	90	90	90	88.7062(17)
V [Å^3]	4583.36(10)	2539.68(5)	2240.28(6)	1328.08(5)
Z	8	4	4	2
ρ_{calcd} [g cm^{-3}]	2.216	2.162	1.813	1.923
μ [mm^{-1}]	8.531	29.401	6.162	5.256
F(000)	2832.0	1544.0	1196.0	748.0
Radiation	MoKα (λ = 0.71073)	CuKα (λ = 1.54184)	MoKα (λ = 0.71073)	MoKα (λ = 0.71073)
θ range [°]	5.192-66.2	9.668-147.108	5.062 to 65.382	5.294 to 75.372
	-34 ≤ h ≤ 35	-10 ≤ h ≤ 11	-12 ≤ h ≤ 13	-14 ≤ h ≤ 14
Limiting indices	-11 ≤ k ≤ 11	-10 ≤ k ≤ 10	-28 ≤ k ≤ 28	-16 ≤ k ≤ 16
	-24 ≤ l ≤ 23	-41 ≤ l ≤ 41	-19 ≤ l ≤ 19	-26 ≤ l ≤ 26
Collected reflns	37770	43995	36123	66113
Unique reflns	6241 [R_{int} = 0.0274]	5081 [R_{int} = 0.0657]	7716 [R_{int} = 0.0360]	13636 [R_{int} = 0.0501]
Data/restraints/parameters	6241/0/249	5081/0/293	7716/0/276	13636/0/357
Goodness of fit	1.057	1.149	1.297	1.059
R_1/wR_2[I>2σ(I)]	0.0199/0.0401	0.0255/0.0610	0.0245/0.0684	0.0277/ 0.0595
R_1/wR_2(all data)	0.0257/0.0420	0.0262/0.0613	0.0350/0.0991	0.0340/ 0.0618
Largest diff. peak/hole/e Å^{-3}	0.81/-0.96	1.26/-1.29	1.58/-2.96	2.63/-1.15

Table S3. Selected bond lengths [Å] and angles [°] for iodido complexes **1′**·MeOH, **2**, **3**·MeOH, **5**·MeOH, **7′**, and **8′**.

	**1′**·MeOH	**2**	**3**·MeOH	**5**·MeOH	**7′**	**8′**
Ir-C(centroid)	1.823	1.819	1.828	1.870	1.817	1.829
Ir-I	2.68371(16)	2.69042(16)	2.6817(3)	2.6799(5)	2.6851(2)	2.6943(3)
Ir-N1	2.0799(19)	2.0624(17)	2.082(3)	2.082(4)	2.065(2)	2.068(3)
Ir-N8	2.0479(18)	2.0664(17)	2.042(3)	2.032(4)	2.049(2)	2.065(3)
N7-N8	1.281(3)	1.297(2)	1.277(5)	1.282(6)	1.270(3)	1.278(5)
N1-Ir-N8	74.72(7)	75.11(7)	74.77(12)	74.68(18)	74.66(9)	74.69(13)
N1-Ir-I	85.23(5)	85.80(5)	86.35(8)	86.84(11)	85.16(6)	86.81(9)
N8-Ir-I	89.01(5)	90.32(5)	88.07(8)	89.26(12)	90.65(6)	88.28(9)

Table S4. Selected bond lengths [Å] and angles [°] for chlorido complexes **9′**·MeOH, and **10**.

	**9′**·MeOH	**10**
Ir-C(centroid)	1.811	1.811
Ir-Cl	2.3888(8)	2.3719(5)
Ir-N1	2.065(3)	2.0655(17)
Ir-N8	2.079(3)	2.0603(17)
N7-N8	1.283(3)	1.283(3)
N1-Ir-N8	74.77(12)	74.69(7)
N1-Ir-Cl	87.54(8)	85.45(5)
N8-Ir-Cl	91.50(9)	89.06(5)

Table S5. Absorption wavelengths and molar extinction coefficients for complexes **1-6** containing HO-azpy-R_2 (pH = 3.0 ± 0.1) and O^--azpy-R_2 (pH = 11.0 ± 0.3) in water/acetone (5/95, v/v). [sh]: shoulder.

Complex at pH 3.0	λ_{max}/nm	ε/ $M^{-1}\cdot cm^{-1}$	Complex at pH 11.0	λ_{max}/nm	ε/ $M^{-1}\cdot cm^{-1}$
**1**(OH)	307	8500	**1**(O^-)	310	9700
	439	19300		558(sh)	35500
**2**(OH)	308	14900	**2**(O^-)	310	13200
	443	25800		563(sh)	43900
**3**(OH)	310	10000	**3**(O^-)	319	9200
	450	20700		573(sh)	35800
**4**(OH)	307	23600	**4**(O^-)	309	28600
	455	40500		586(sh)	56300
**5**(OH)	298	12600	**5**(O^-)	299	8100
	459	14400		579	26900
**6**(OH)	306	23600	**6**(O^-)	307	36000
	464	40000		571	57200

Table S6. The extent of adduct formation and the detected ESI-MS peaks for reactions of iodido complex **3** and chlorido complex **9** with 9-EtG, N-acetyl-L-methionine (Ac-Met) and L-histidine (His).

**Adducts**	**Extent[a] (%)**	**Detected[b] m/z**	**Calcd m/z**
[Cp*(Ir-His)(O⁻-azpy-Br)]⁺(**3**_His)	7	758.92⁺	759.12⁺
[Cp*(Ir-Ac_Met)(O⁻-azpy-Br)]⁺(**3**_Ac-Met)	29	795.11⁺	795.11⁺
[Cp*(Ir-9-EtG)(O⁻-azpy)]⁺ (**9**_9-EtG)	15	705.10	705.22⁺
[Cp*(Ir-His)(O⁻-azpy)]⁺ (**9**_His)	100	681.11⁺	681.21⁺
[Cp*(Ir-Ac_Met)(O⁻-azpy)]⁺ (**9**_Ac-Met)	100	717.06⁺	717.20⁺

[a] Determined by integration of ^{1}H NMR peaks

[b] The MS peak of the adduct was detected by LC-MS

Table S7. Values of IC_{50} ± mean SD for ten iridium complexes toward human A549 lung cancer cells in comparison with cisplatin (CDDP). IC_{50}: Half maximal inhibitory concentrations, determined from duplicates of triplicate experiments.

Complex	IC_{50} (μM)	Complex	IC_{50} (μM)
**1**	1.01 ± 0.08	**6**	0.55 ± 0.01
**2**	0.83 ± 0.06	**7**	8.84 ± 0.29
**3**	0.33 ± 0.01	**8**	21.73 ± 1.52
**4**	0.36 ± 0.04	**9**	1.5 ± 0.3
**5**	1.59 ± 0.07	**10**	0.89 ± 0.03
**CDDP**	3.30 ± 0.10		

Table S8. ROS induction in A549 cancer cells treated with complexes **3** and **7** at 2x IC_{50} concentrations. FL1 channel detects superoxide production and FL2 channel detects total oxidative stress. Cell populations are in %. *p*-Values were calculated after a t-test against the negative control data, *p< 0.05, **p<0.01.

Cell Population	Cell treatment (dose)		
(%)	Untreated	3 (2x IC_{50})	7 (2x IC_{50})
**FL1-/FL2+**	0.17 ± 0.027	0.58 ± 0.34	1.14 ± 0.19*
**FL1+/FL2+**	4.91 ± 1.29	8.21 ± 1.14*	8.61 ± 0.55*
**FL1+/FL2-**	37.48 ± 0.95	38.74 ± 5.50	49.81 ± 2.44**
**FL1-/FL2-**	57.51 ± 1.80	52.48 ± 4.80	40.44 ± 1.99**

Table S9. The partition coefficients (log $P_{7.4}$) between octanol and phosphate buffer water (2 mM, pH 7.4) at 298 K for complexes **3** and **7**, and the cellular iridium accumulation in A549 lung cancer cells treated with complex **3** or **7** at equipotent IC_{50} concentrations for 24 h.

**Complex**	**log $P_{7.4}$**	**IC_{50} (μM)**	**Ir Accumulation ($ng/10^6$ cells)**
$[(Cp^*)Ir(O^-\text{-azpy-Br})I]PF_6$ (**3**)	-0.08	0.33 ± 0.01	101 ± 1.3
$[(Cp^*)Ir(azpy)I]PF_6$ (**7**)	-0.59	8.84 ± 0.29	51.3 ± 10.1

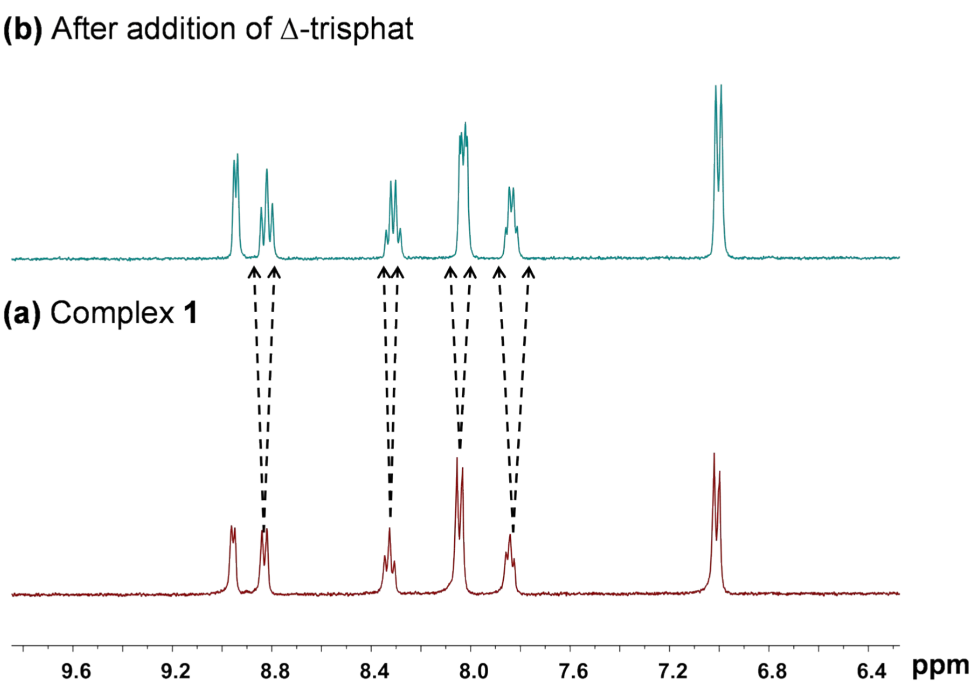

Figure S1 ^{1}H NMR (400 MHz, d_4-MeOD) of (a) complex **1** (ca. 1 mM); (b) complex **1** with 3 mol equiv. Δ-trisphat tetrabutylammonium salt at 298 K, showing the splitting of peaks due to the presence of the two enantiomers of **1**.

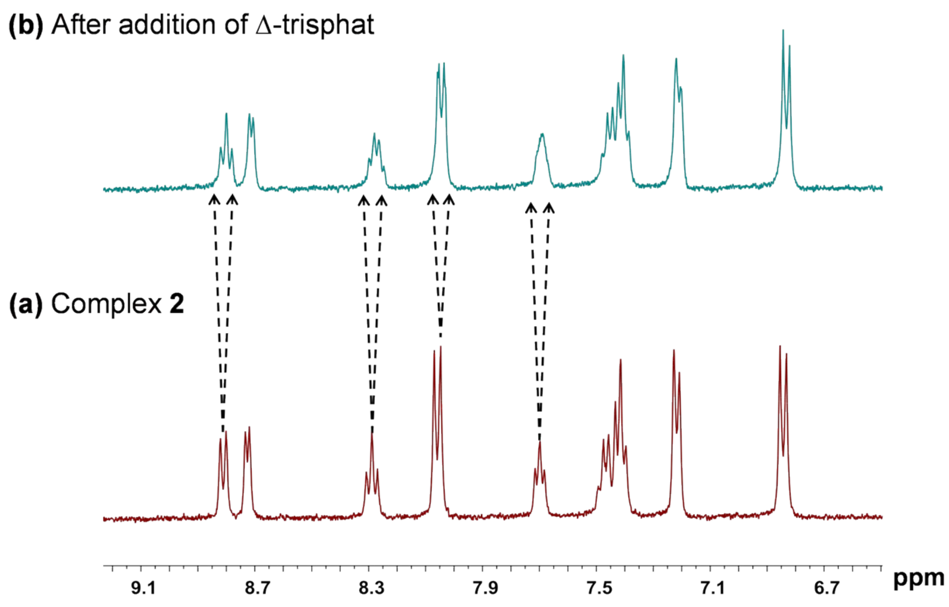

Figure S2. ^{1}H NMR (400 MHz, d_4-MeOD) of (a) complex **2** (ca. 1 mM); (b) complex **2** with 5 mol equiv. Δ-trisphat tetrabutylammonium salt at 298 K, showing the splitting of peaks due to the presence of the two enantiomers of **2**.

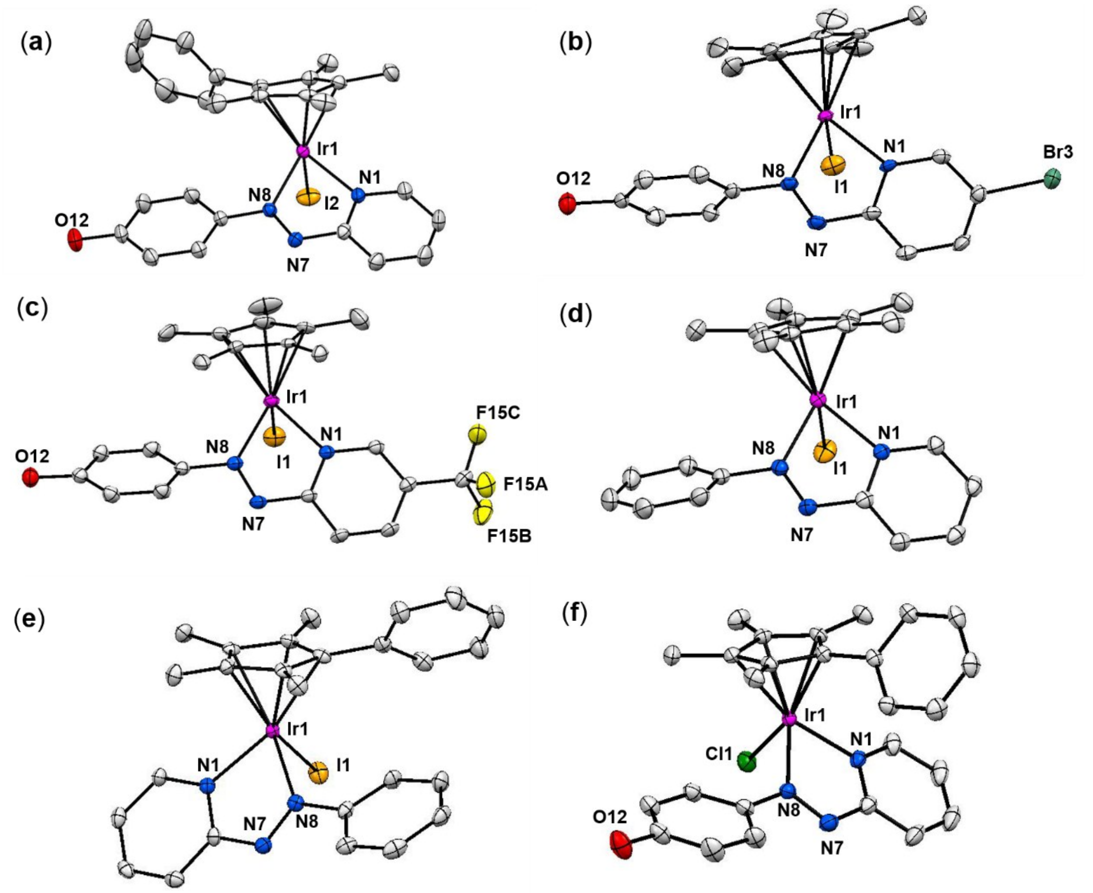

Figure S3. ORTEP views of the X-ray crystal structures with thermal ellipsoids drawn at 50% probability for complexes (a) [(η^5-Cp^{xph})Ir(HO-azpy)I]PF_6 (**2**), (b) [(η^5-Cp*)Ir(HO-azpy-Br)I]PF_6·MeOH (**3**·MeOH), (c) [(η^5-Cp*)Ir(HO-azpy-CF_3)I]PF_6·MeOH (**5**·MeOH), (d) [(η^5-Cp*)Ir(azpy)I]I (**7′**), (e) [(η^5-Cp^{xph})Ir(azpy)I]I (**8′**) and (f) [(η^5-Cp^{xph})Ir(azpy)Cl]PF_6 (**10**). The hydrogen atoms, counter anion and solvent molecules have been omitted for clarity.

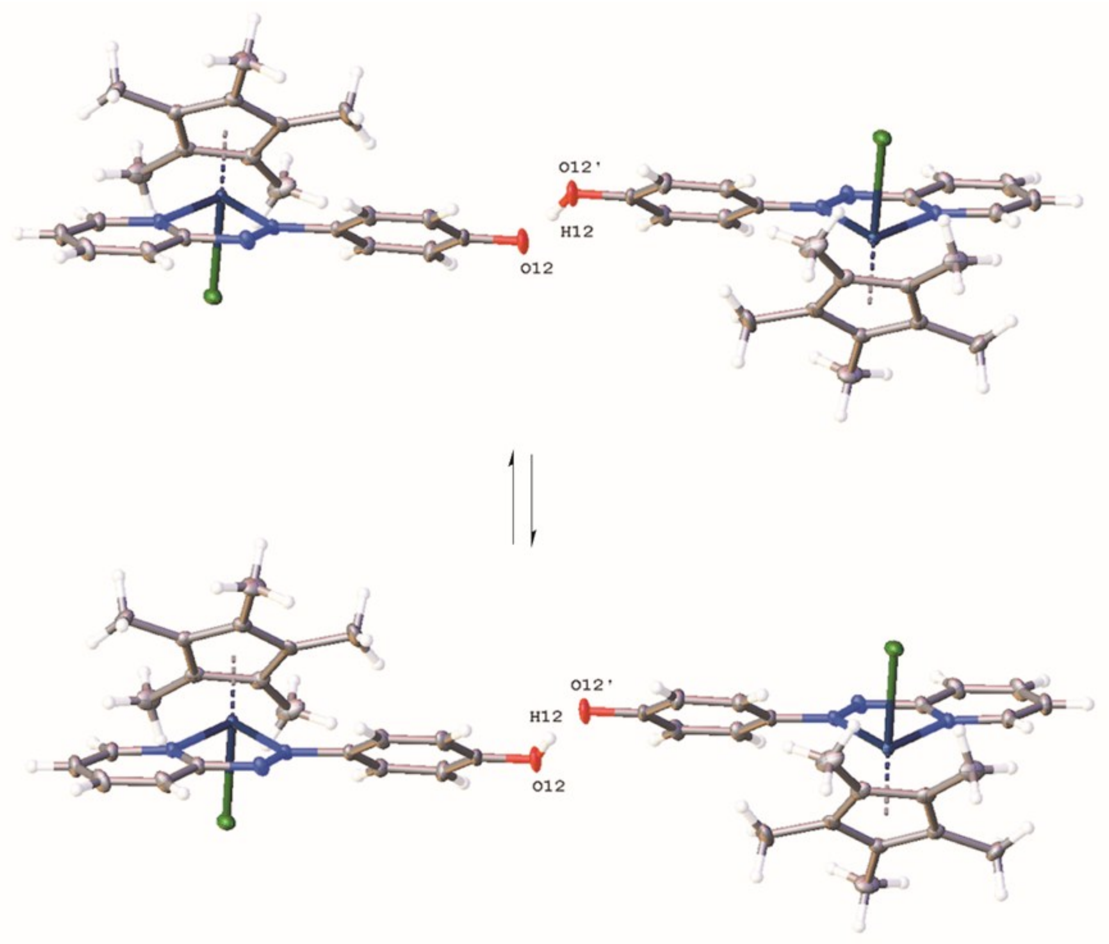

Figure S4. The two tautomer positions of the proton H12 in the crystal structure of [(η^5-Cp*)Ir(HO-azpy)Cl]Cl·MeOH (**9′**·MeOH) shared between phenolic oxygen O12 and a symmetry related phenolic oxygen (O12’). H12 was refined at 50% occupancy to model the two possible tautomeric positions of the hydrogen as the oxygens are related by an inversion centre.

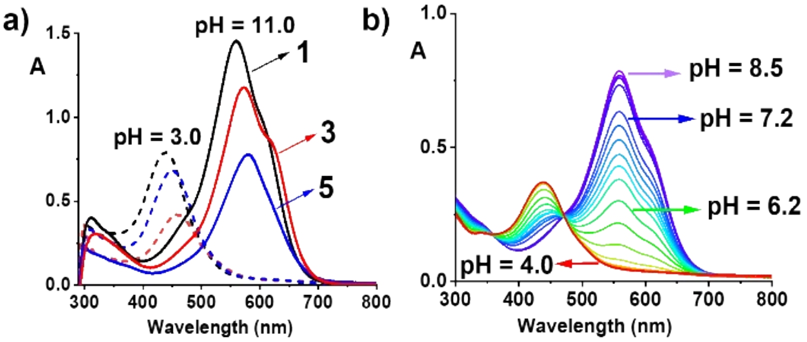

Figure S5. (a) UV-vis spectra of complexes [(η^5-Cp*)Ir(HO-azpy)I]PF_6 (**1**) (41 μM, black), [(η^5-Cp*)Ir(HO-azpy-Br)I]PF_6 (**3**) (33 μM, red) and [(η^5-Cp*)Ir(HO-azpy-CF_3)I]PF_6 (**5**) (29 μM, blue) at pH = 3.0 ± 0.3 (dash lines) and pH = 11.0 ± 0.3 (solid lines) in water/acetone (95/5 v/v); (b) UV-vis changes for complex **1** from pH 4 to 8.5. At pH = 3.0, the spectra of **1**, **3**, and **5**, display a MLCT (metal-to-ligand charge-transfer) absorption band at 439-464 nm (ε= 8000-20700 $M^{-1}\cdot cm^{-1}$, Table S5). Raising the pH to 11.0 led to a dramatic red-shift of this MLCT band to 558-586 nm with a much higher extinction coefficient (26900-35800 $M^{-1}\cdot cm^{-1}$, Table S5).

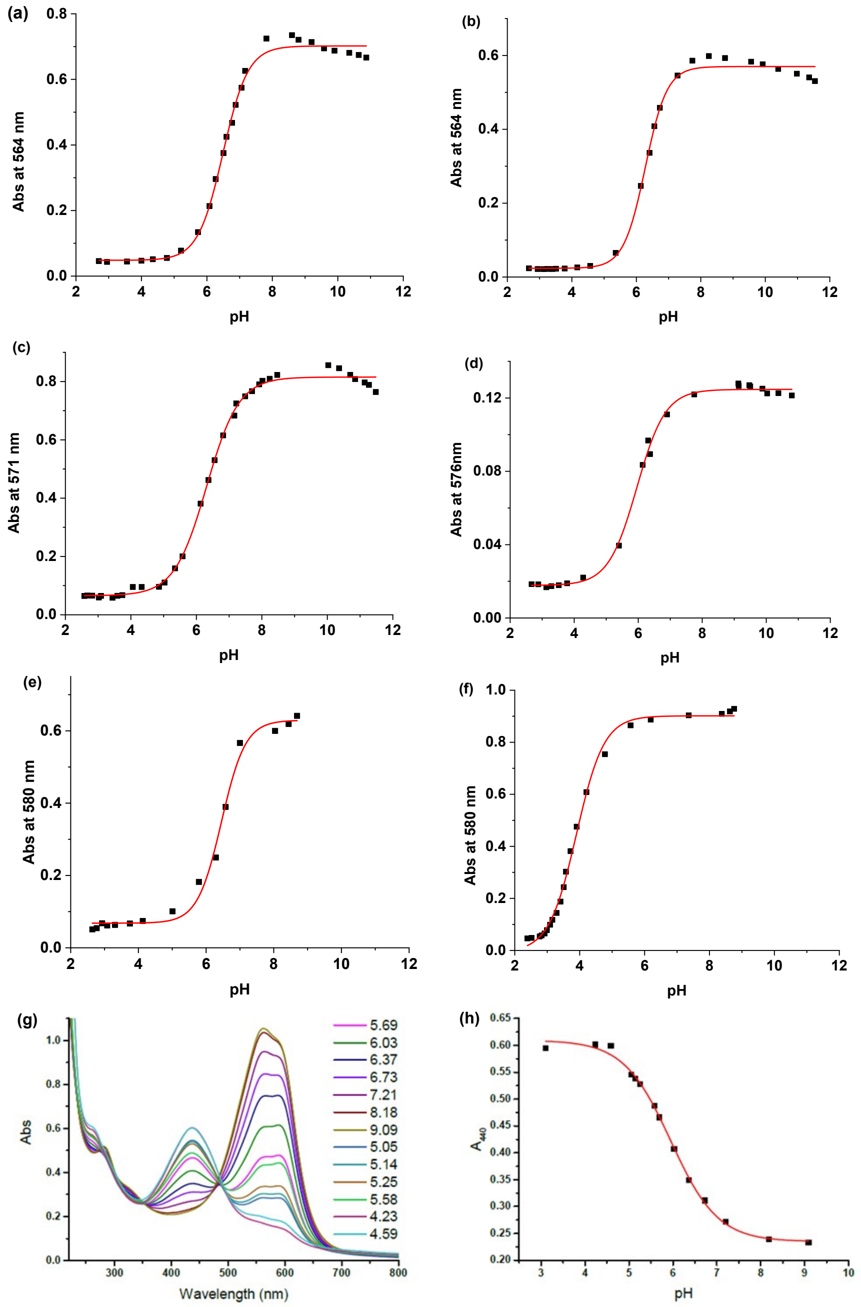

Figure S6. Dependence on pH of the UV-vis absorbance of complexes in MeOH/H_2O (1/99, v/v) at selected wavelengths. (a) [(η^5-Cp*)Ir(HO-azpy)I]PF_6 (**1**); (b) [(η^5-Cp^{xph})Ir(HO-azpy)I]PF_6 (**2**); (c) [(η^5-Cp*)Ir(HO-azpy-Br)I]PF_6 (**3**); (d) [(η^5-Cp^{xph})Ir(HO-azpy-Br)I]PF_6 (**4**); (e) [(η^5-Cp*)Ir(HO-azpy-CF_3)I]PF_6 (**5**); (f) [(η^5-Cp^{xph})Ir(HO-azpy-CF_3)I]PF_6 (**6**); (g) dependence of the UV-vis spectrum on pH for complex [(η^5-Cp*)Ir(azpy-OH)Cl]PF_6 (**9**); (h) curve fit for change in absorbance at 440 nm with pH for **9**, corresponding to a pK_a of 5.88 ± 0.02.

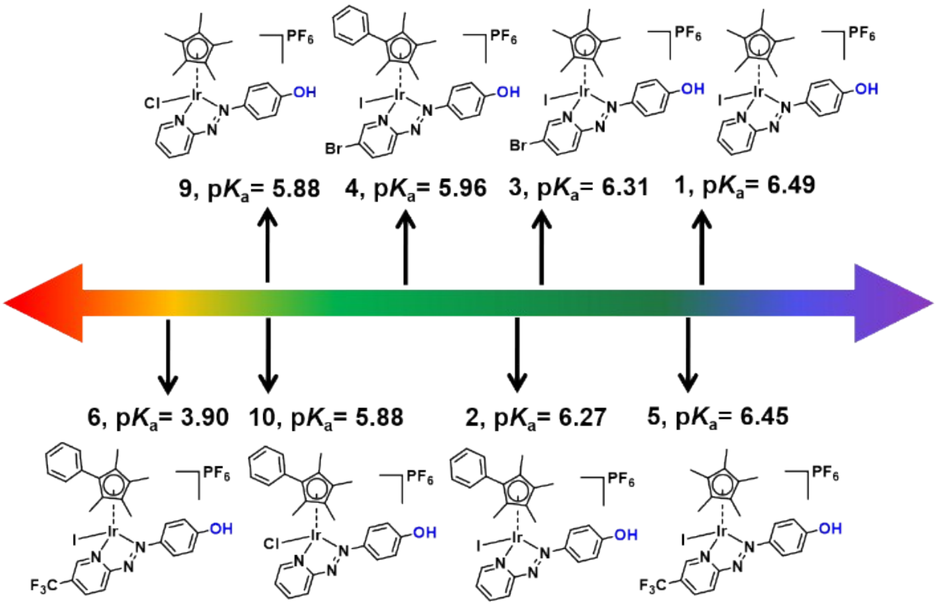

Figure S7. pK_a values of phenolic groups in iodido complexes **1**-**6** and chlorido complexes **9**-**10**. The changes of UV-vis absorbance with pH (Figs S5 and S6) allowed determination of the pK_a values as 5.88-6.49, with the exception of the more acidic [(Cp^{xph})Ir(CF$_3$-azpy-OH)I]PF$_6$ (**6**), with a pK_a 3.90. Compared to the pK_a values of iodido Cp* analogues, all those for the iodido Cp^{xph} complexes are lower.

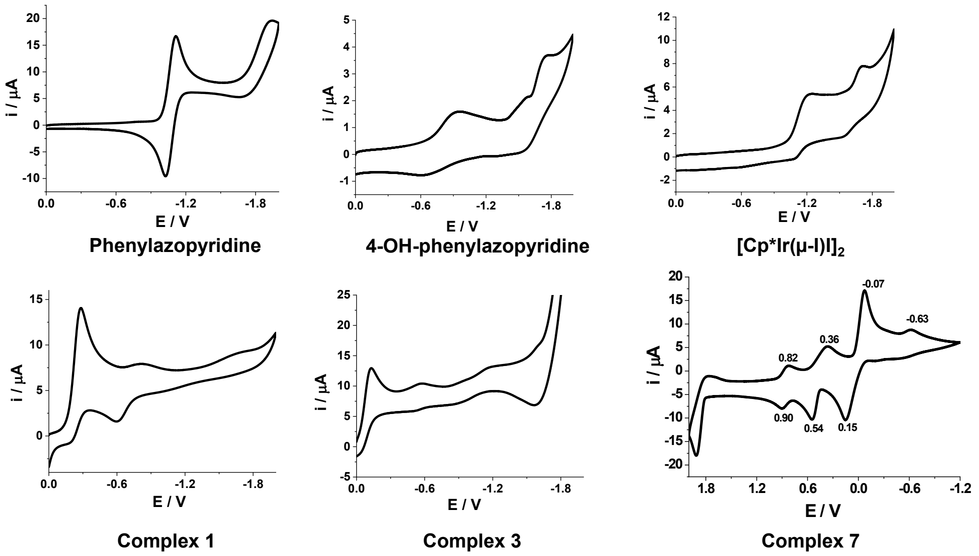

Figure S8. Electrochemical cyclic voltammograms for selected ligands and complexes, showing complexes **1**, **3**, and **7** undergo more facile sequential reduction than the free phenylazopyridine ligand as well as the iridium precursor $[Cp^{*}Ir(\mu\text{-}I)I]_2$. The second step of reduction for complex **7** is irreversible due to dissociation of the two-electron reduction product phenylhydrazopyridine from the metal.

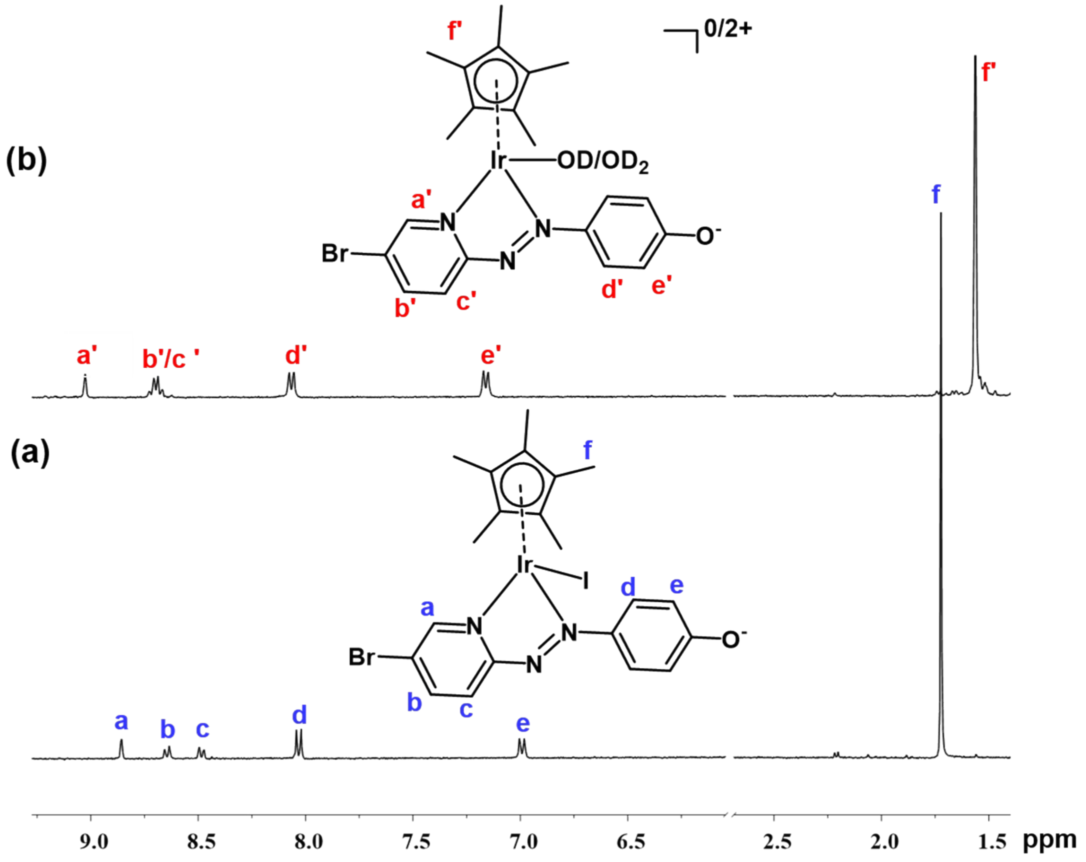

Figure S9. ^{1}H NMR (400 MHz, 298 K) of (a) **3** alone at 310 K after 24 h; (b) **3** with 1.2 equiv. $AgNO_3$ in d$_4$-MeOD/D_2O (1/1, v/v) at 310 K after 24 h, showing complex **3** is inert towards hydrolysis. At neutral pH, *ca.* 83% of the phenol groups (pK_a = 6.31) exist in a deprotonated form.

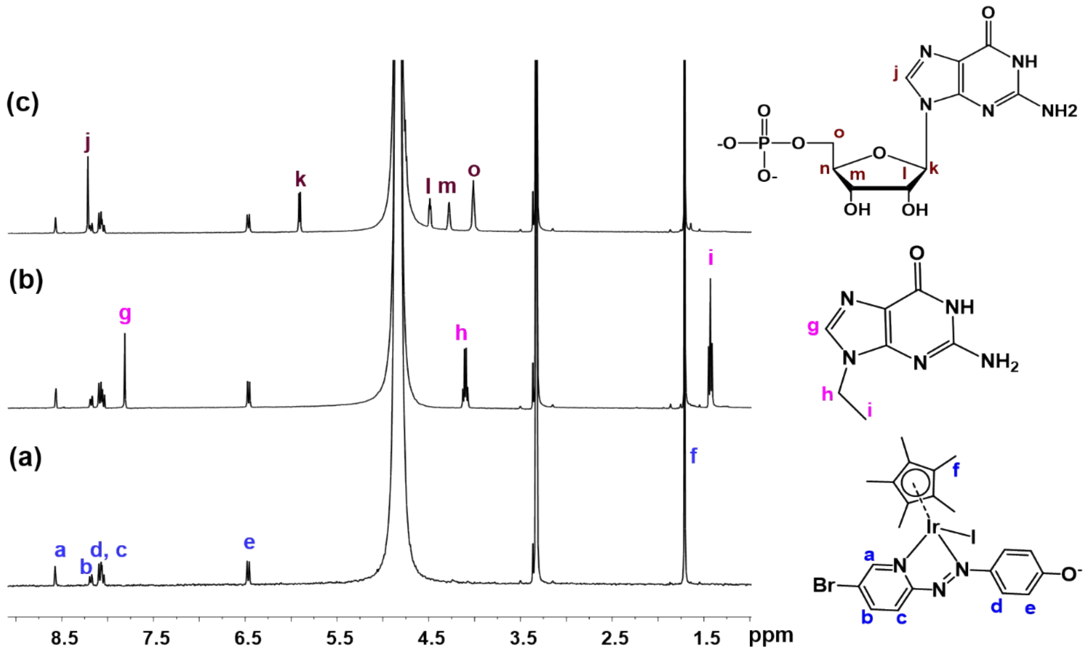

Figure S10. ^{1}H NMR (400 MHz, d_4-MeOD/0.1 M phosphate-buffered D_2O, 1/1, v/v, pH* 7.8) of (a) **3** at 310 K after 24 h; (b) complex **3** with 3.0 mol equiv. 9-EtG at 310 K after 24 h; (c) **3** with 3.0 mol equiv. 5'-GMP at 310 K after 24 h, showing complex **3** is inert to hydrolysis or binding to nucleobases.

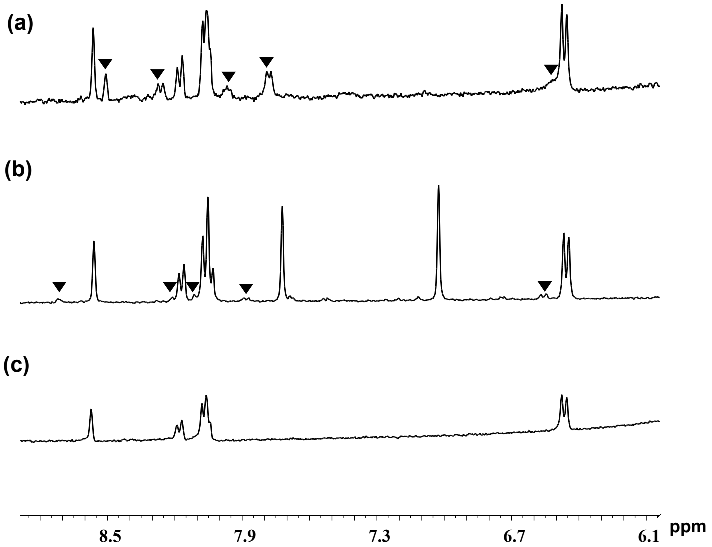

Figure S11. ^{1}H NMR spectra (400 MHz, d_4-MeOD/0.1 M phosphate-buffered D_2O, 1/1, v/v, pH* 7.8) showing the aromatic region of (a) complex **3** with 3.0 mol equiv. N-acetyl L-methionine at 310 K after 24 h; (b) **3** with 3.0 mol equiv. L-histidine at 310 K after 24 h; (c) **3** at 310 K after 24 h. Arrows indicate new peaks for minor adducts of **3** with the amino acid confirmed by ESI-MS (Table S6).

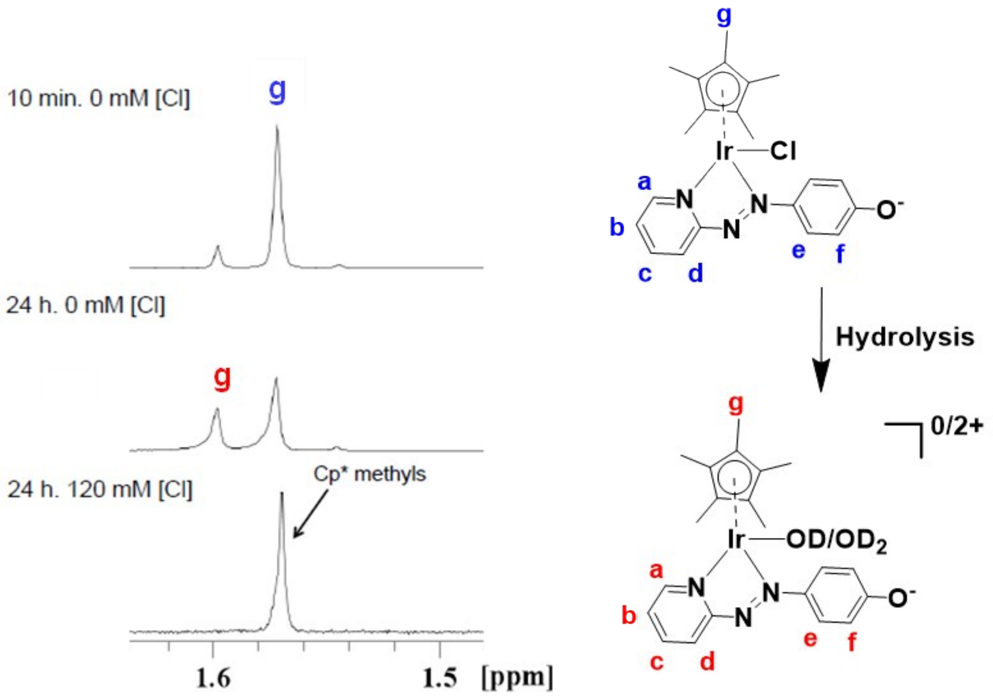

Figure S12. Hydrolysis of complex **9** (100 µM) studied by 600 MHz ^{1}H NMR spectra in d_6-DMSO/D_2O (1/9, v/v) with 0.1% 1,4-dioxane (v/v) at 310 K. Spectra shown 10 min and 24 h after sample preparation, and 24 h after sample preparation in 120 mM NaCl solution. Peaks assigned with red letters correspond to the hydrolyzed form and with blue letters to the original complex **9**. At neutral pH, *ca.* 93% of the phenol groups (pK_a = 5.88) exist in a deprotonated form.

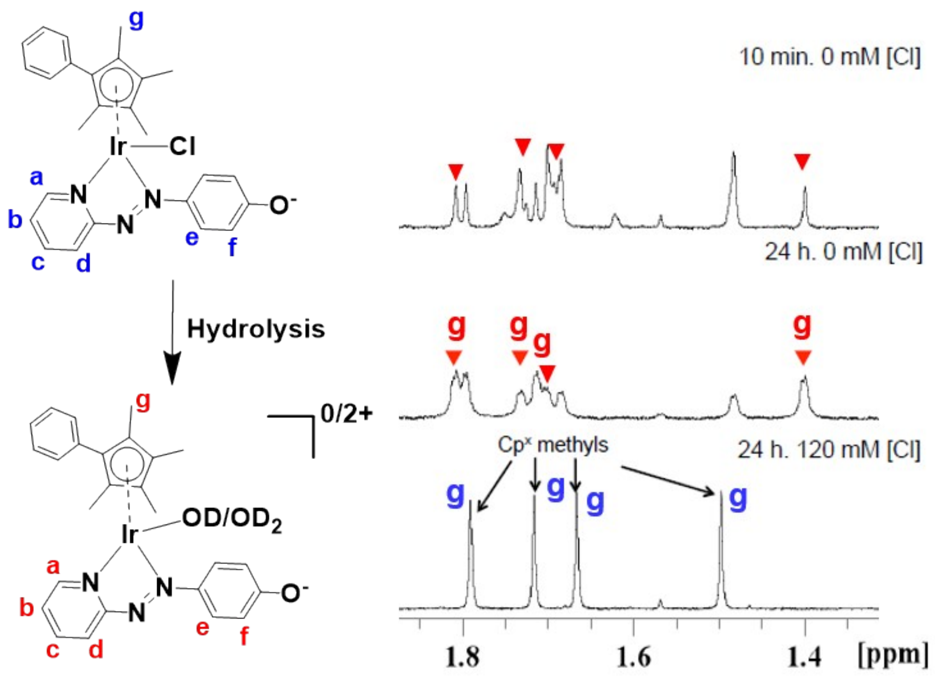

Figure S13. Hydrolysis of complex **10** (100 μM) studied by 600 MHz ^{1}H-NMR spectra in d_6-DMSO/D_2O (1/9, v/v) with 0.1% 1,4-dioxane (v/v) at 310 K. Spectra were recorded 10 min and 24 h after sample preparation, and 24 h after sample preparation in 120 mM NaCl solution. Peaks assigned with red letters correspond to the hydrolyzed form and with blue letters to the original complex **10**. At neutral pH, *ca.* 93% of the phenol groups (pKa = 5.88) exist in a deprotonated form.

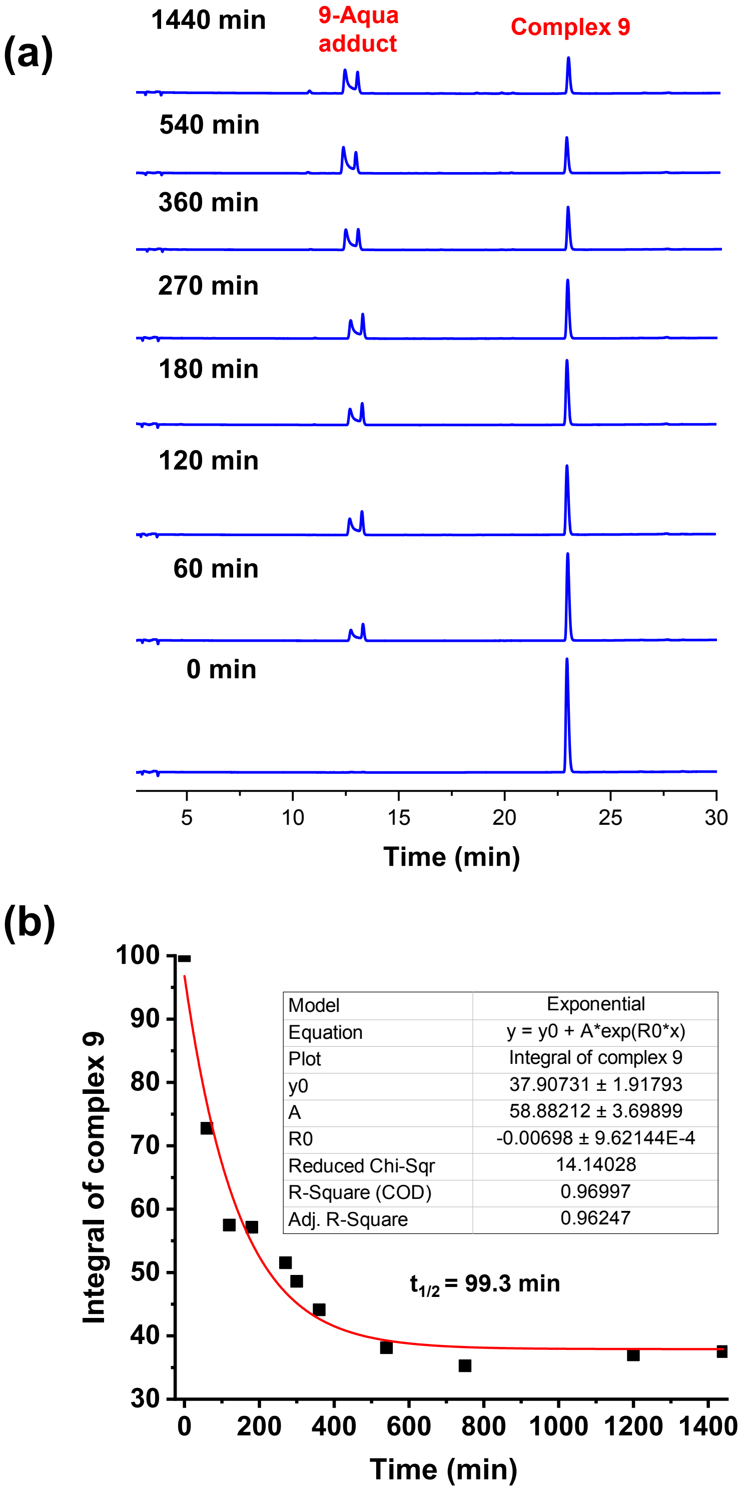

Figure S14. (a) Time-dependent HPLC chromatograms (detection wavelength 254 nm) of complex **9** (50 μM) in MeOH/H_2O (5/95, v/v) at 310 K. (b) First-order kinetic fitting of the time versus the corresponding HPLC peak integral for complex **9** (confirmed by ESI-MS).

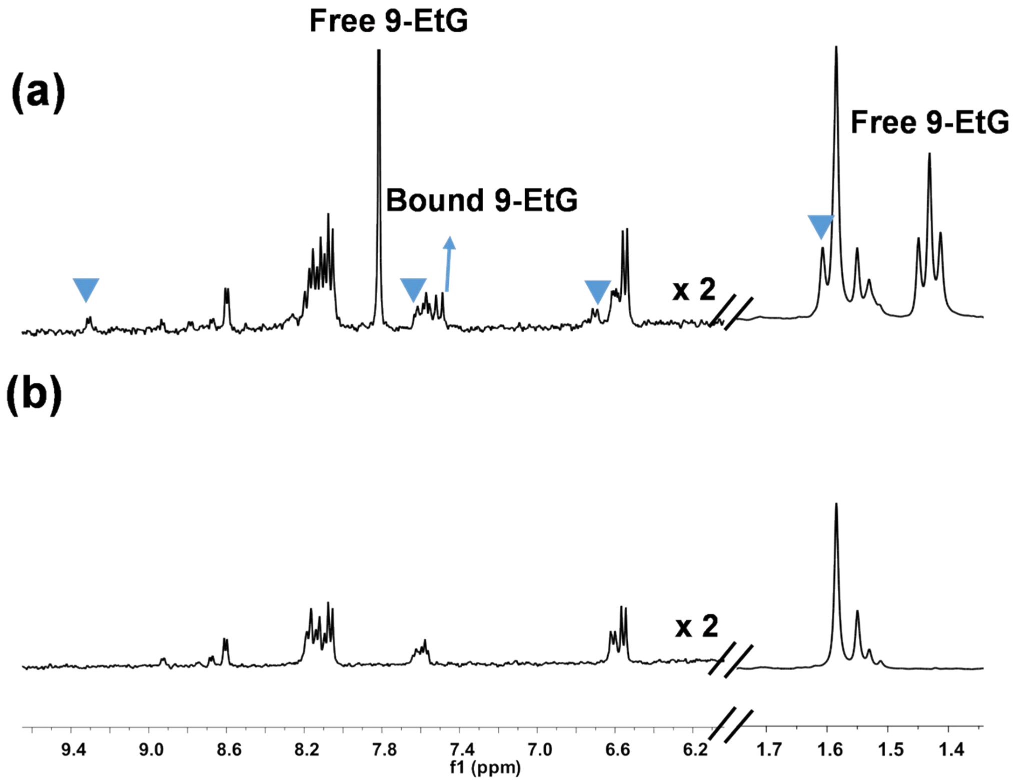

Figure S15. ^{1}H NMR (400 MHz, d_4-MeOD/0.1 M phosphate-buffered D_2O, 1/1, v/v, pH* 7.8) of (a) complex **9** with 3.0 mol equiv. 9-EtG at 310 K after 24 h; (b) complex **9** at 310 K after 24 h. The blue triangles indicate the adduct **9**_9-EtG.

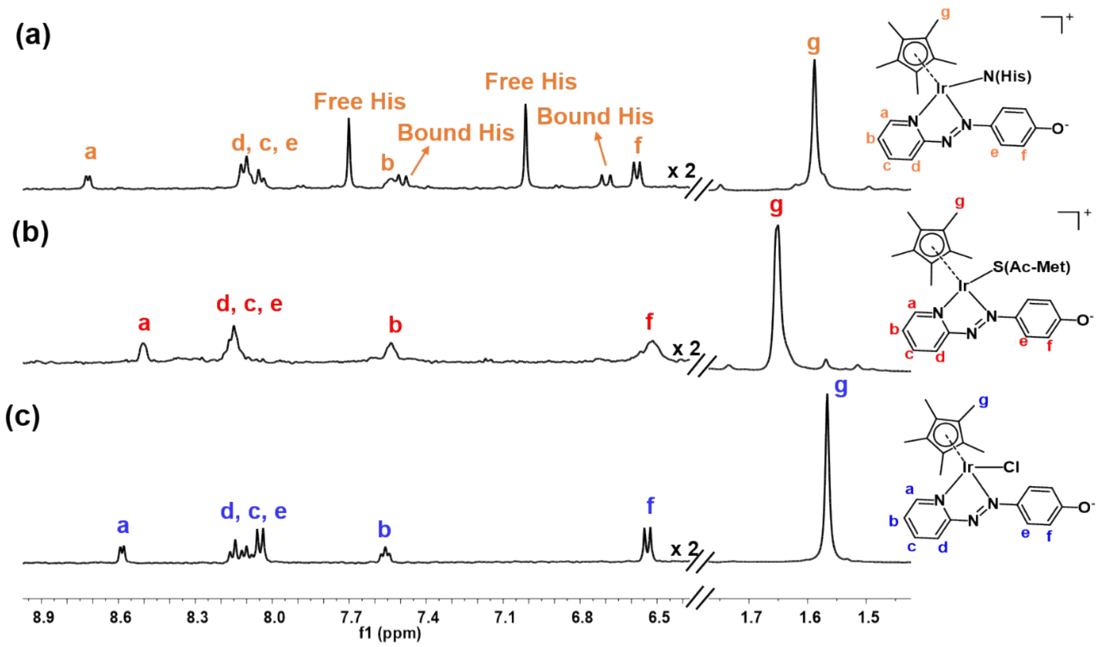

Figure S16. ^{1}H NMR spectra (400 MHz, d_4-MeOD/0.1 M phosphate-buffered D_2O, 1/1, v/v, pH* 7.8) showing the aromatic region for (a) complex **9** with 3.0 mol equiv. L-histidine (His) at 310 K after 24 h; (b) **9** with 3.0 mol equiv. N-acetyl-L-methionine (Ac-Met) at 310 K after 24 h; (c) **9** alone after 5 mins.

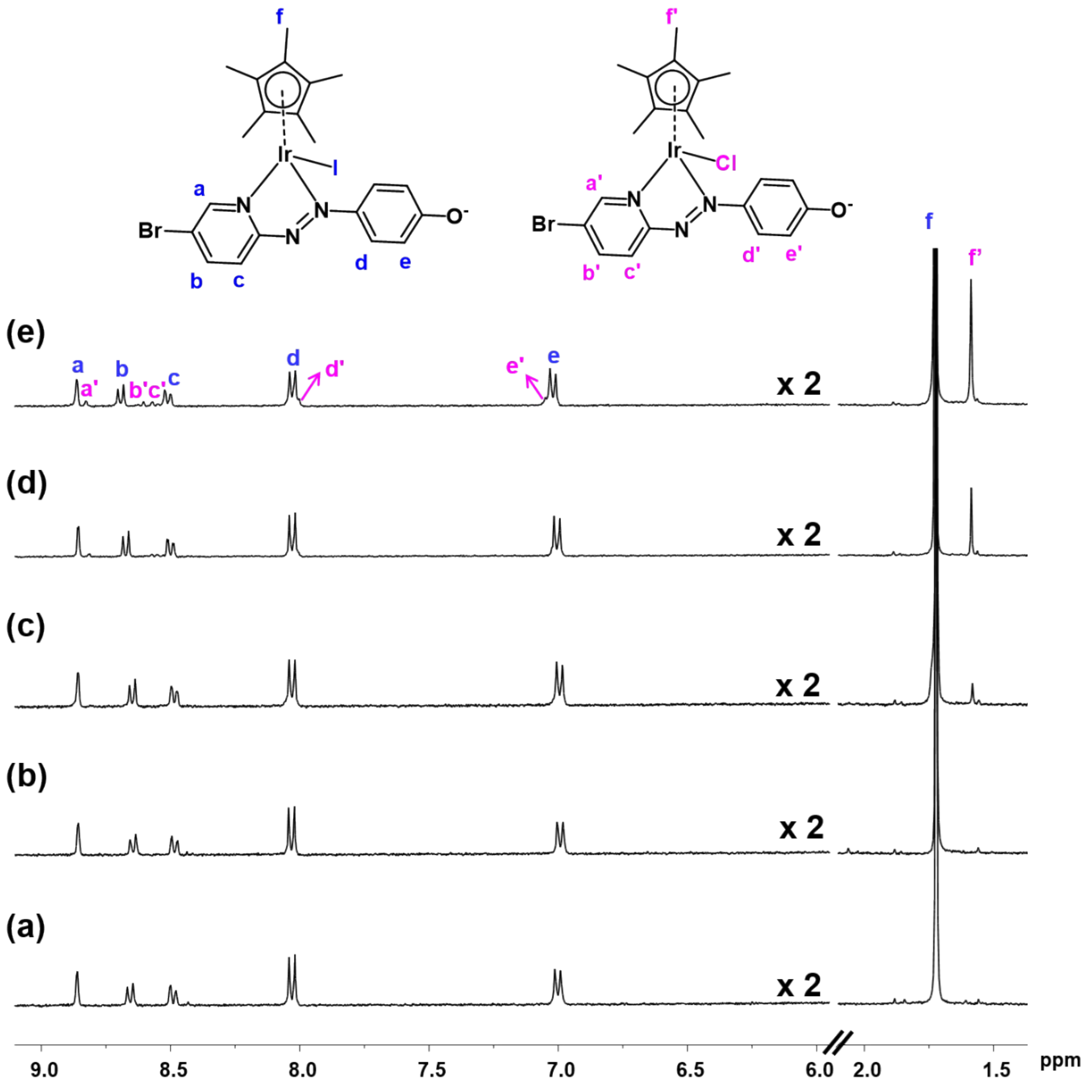

Figure S17. ^{1}H NMR (400 MHz) of iodido complex **3** (ca. 1 mM) in 50% d_4-CD_3OD/5 mM phosphate-buffered D_2O, 1/1, v/v, pH* 7.0. (a) alone after 5 min; (b) alone after 24 h; (c) with 4.0 mM NaCl; (d) with 23.0 mM NaCl; (e) with 103.0 mM NaCl at 310 K after 24 h, showing that complex **3** not only resists hydrolysis (compare (a) and (b)), but also undergoes only minor (< 16.5 % of **3** based on ^{1}H NMR peak integrals) ligand exchange under physiologically-relevant concentrations of chloride.

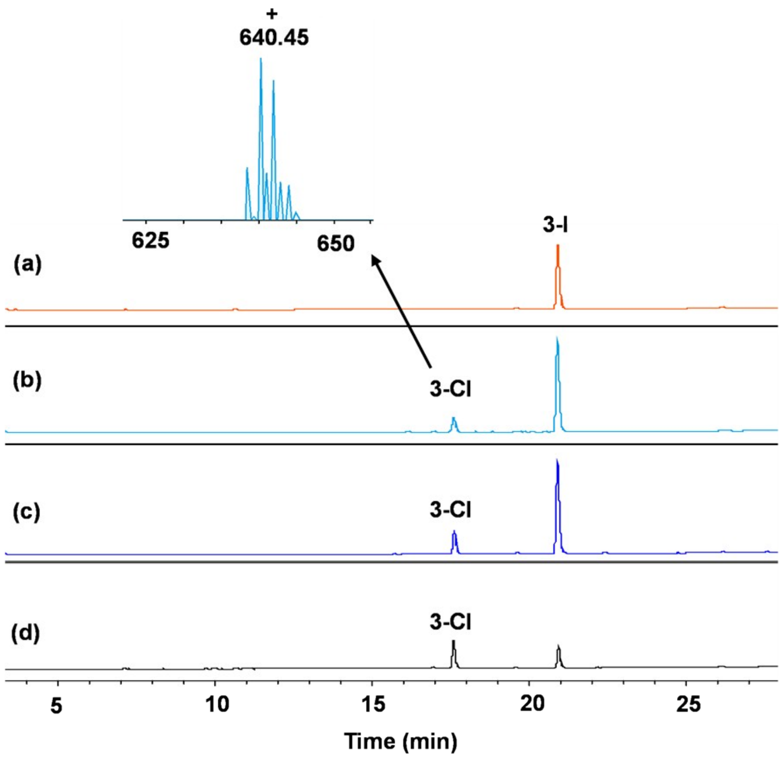

Figure S18. HPLC chromatograms (detection wavelength 254 nm) of iodido complex **3** (ca. 0.1 mM) (a) before, and (b) after incubation with 4.0 mM NaCl, (c) 23 mM NaCl, and (d) 103 mM NaCl, at 310 K for 24 h. The inset shows the detected LC-MS MS peak for the chlorido analogue of complex **3** in 103 mM NaCl solution at 310 K after 24 h.

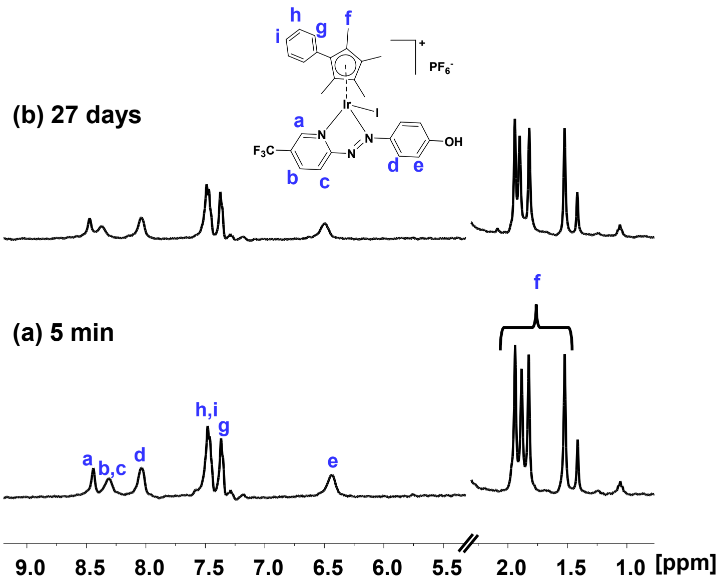

Figure S19. ^{1}H-NMR spectra (400 MHz, d_6-DMSO) of complex **6** after (a) 5 min and (b) 27 days at 298 K. No additional peaks are observed, indicating the stability of complex **6** towards DMSO.

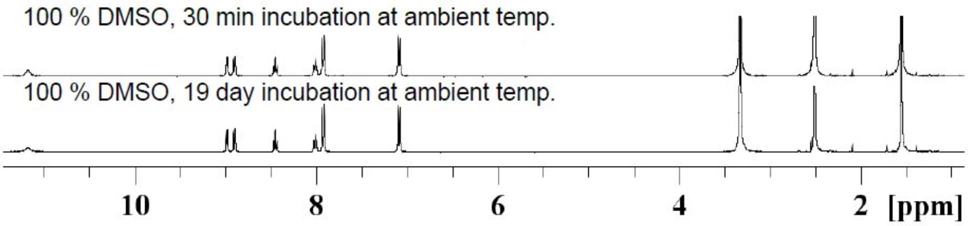

Figure S20. ^{1}H-NMR spectra (400 MHz, d_6-DMSO) of complex **9** after 30 min and 19 days at 298 K. No additional peaks are observed indicating the stability of the complex **9** towards DMSO.

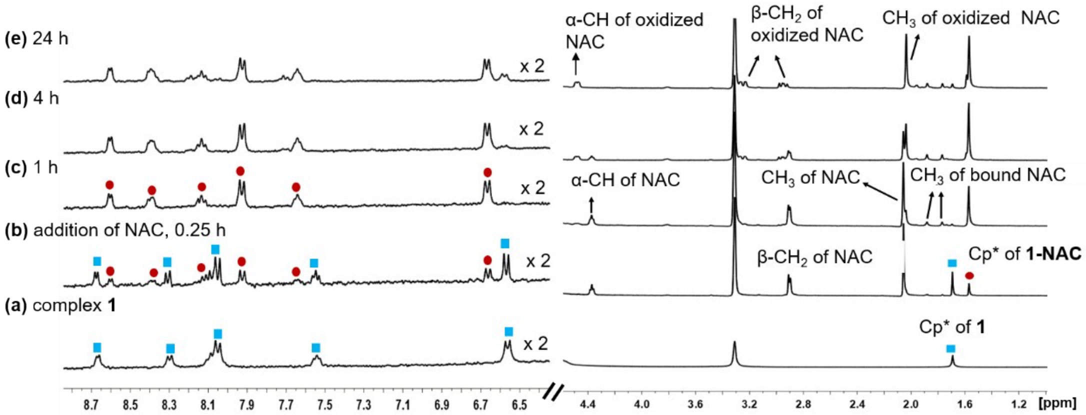

Figure S21. Time-dependent ^{1}H NMR spectra (400 MHz) of (a) complex **1** alone; and complex **1** with 3 mol equiv. NAC at (b) 0.25 h, (c) 1.0 h, (d) 4 h, (e) 24 h in 0.1 M phosphate-buffered D_2O/d_4-MeOD (7/3, v/v, pH* 7.4). Blue squares indicate **1**, and red circles indicate **1-NAC**. The presence of diastereomers of **1-NAC** is indicated by the two sets of peaks in a 1:1 ratio for the acetyl methyl protons of bound NAC.

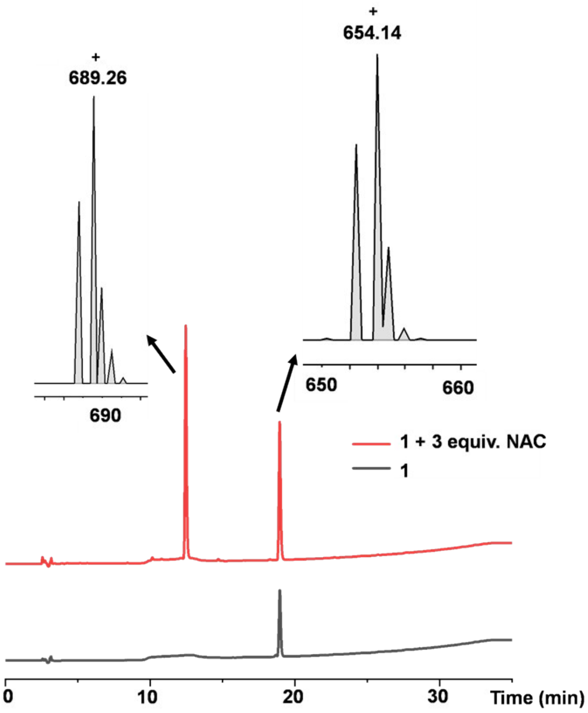

Figure S22. HPLC chromatograms (detection wavelength 254 nm) for complex **1** (black), and complex **1** with 3 mol equiv. NAC (red) after 1 h incubation in CH_3CN/H_2O (1/9, v/v) at 310 K. MS peaks with *m/z* $[654.14]^+$ assignable as $[\mathbf{1}]^+$ and *m/z* $[689.26]^+$ assignable as $[(\mathbf{1}\text{-NAC})\text{-I}]^+$ which indicates the formation of **1-NAC** adducts.

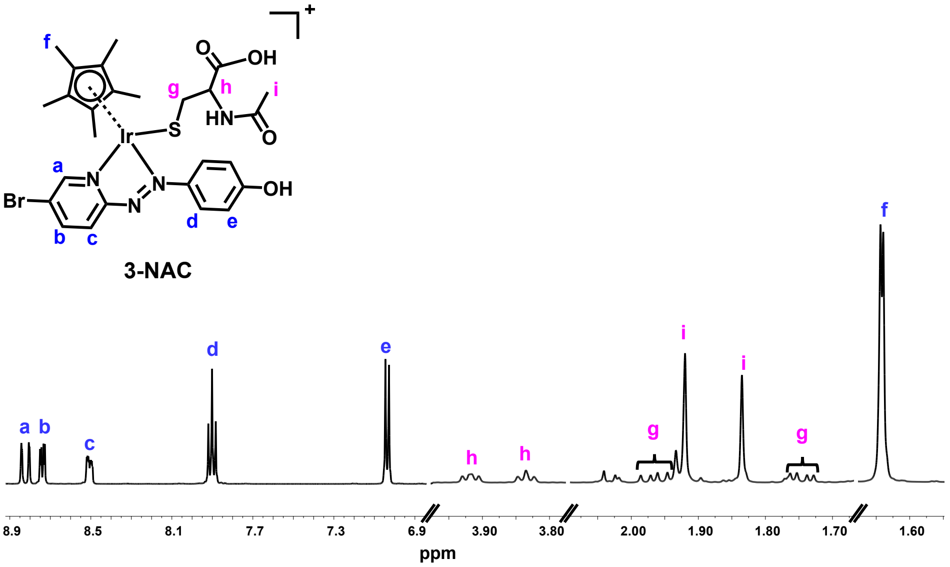

Figure S23. ^{1}H NMR (400 MHz, d_4-MeOD) spectrum of **3-NAC**. The presence of diastereomers is evident from the double sets of peaks for all protons.

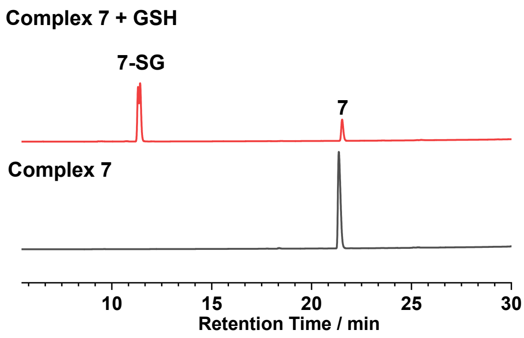

Figure S24. HPLC chromatograms (detection wavelength 254 nm) for complex **7** alone (100 μM) and 15 min after reaction of **7** (1 mM) with 2 mol equiv. GSH in 0.1 M phosphate buffer D_2O/d_4-MeOD (7/3, v/v, pH* 7.4) at 310 K (HPLC sample prepared by 1:10 v/v dilution with MeOH/H_2O (1/9, v/v)).

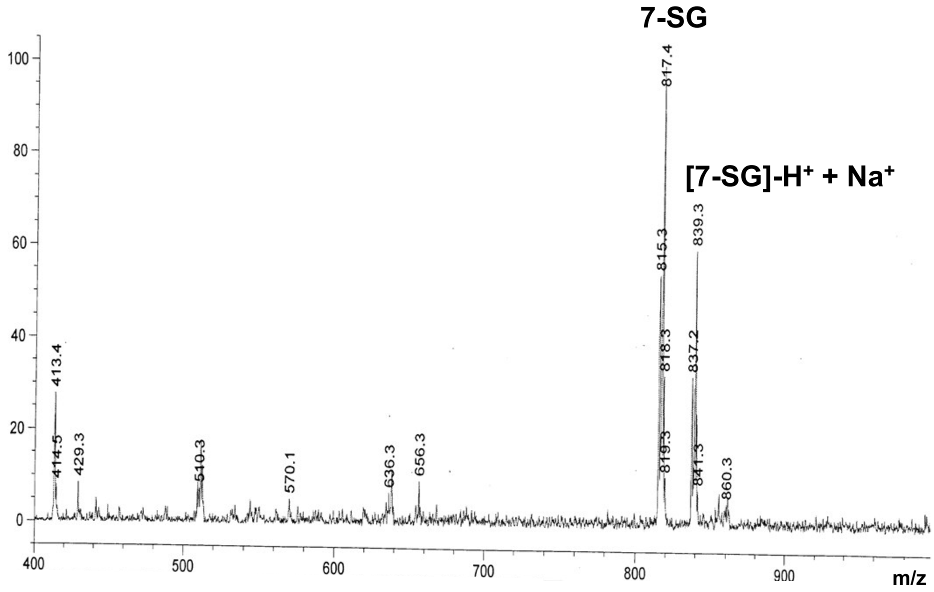

Figure S25. ESI-MS peaks assigned to **7-SG**.

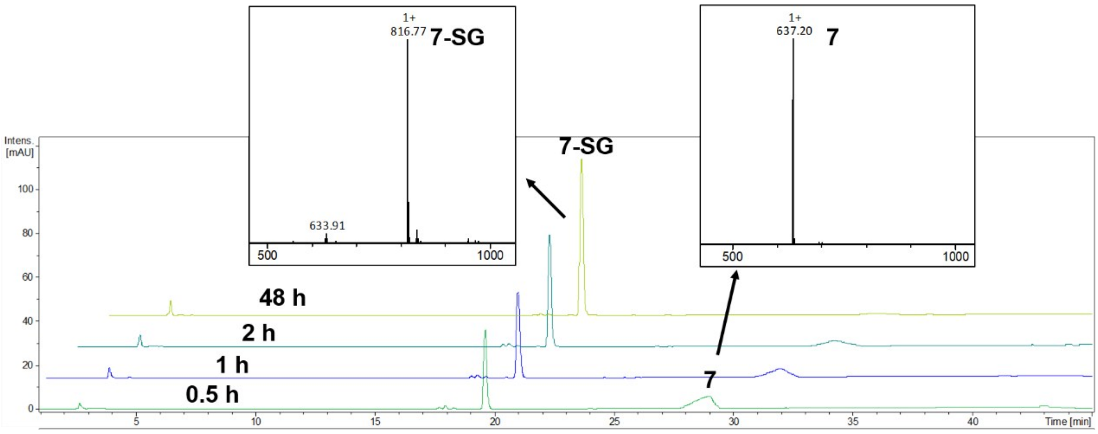

Figure S26. Time-dependent HPLC chromatograms (45° stacked) with ESI-MS peak insets for the reaction of complex **7** (100 μM) with 1 mol equiv. GSH in water at 310 K. Mobile phases acetonitrile/H_2O (without TFA additive), with detection wavelength 254 nm.

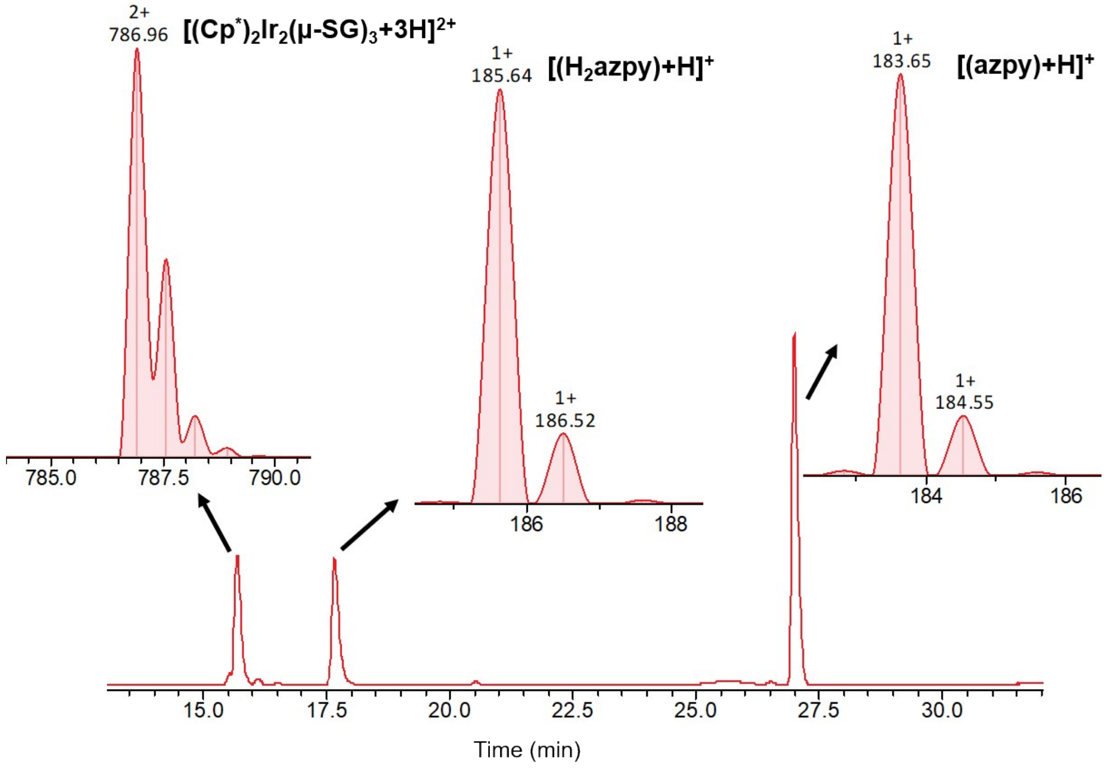

Figure S27. LC-MS study of a ^{1}H NMR sample 3 h after reaction of complex **7** (1 mM) with GSH (10 mM) (see Fig. 4c in main text) indicating the formation of phenyl-hydrazo-pyridine (H_2azpy) and SG-bridged iridium adduct $[(Cp^*)_2Ir_2(\mu\text{-}SG)_3]^+$. Eluents: A-water (0.1% v/v TFA), B-acetonitrile (0.1% TFA). 0-10 min, 2%-15% B; 10-40 min, 15%-80% B; 40-45 min, 80%-2% B; 40-55 min, 2% B.

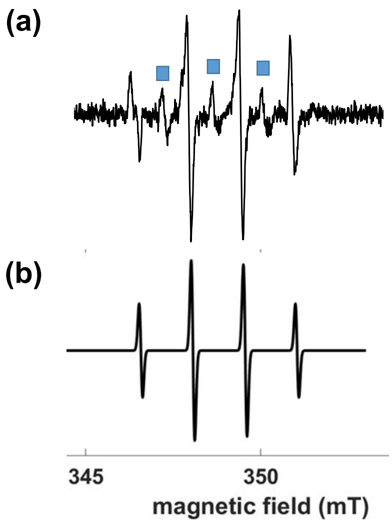

Figure S28. (a) EPR spectrum of hydroxyl radicals trapped as DMPO-OH over the first 1.45 h from reaction of complex **7** (1 mM) with GSH (20 mM) in 0.1 M phosphate buffer (pH 7.4) at ambient temperature in the presence of DMPO (100 mM). (b) Simulated spectrum of DMPO-OH using Easyspin ($a^N = a^{\beta H}$ = 1.50 mT). The blue squares indicate oxidized intermediates[20] arising from DMPO-OH (a^N = 1.51 mT).

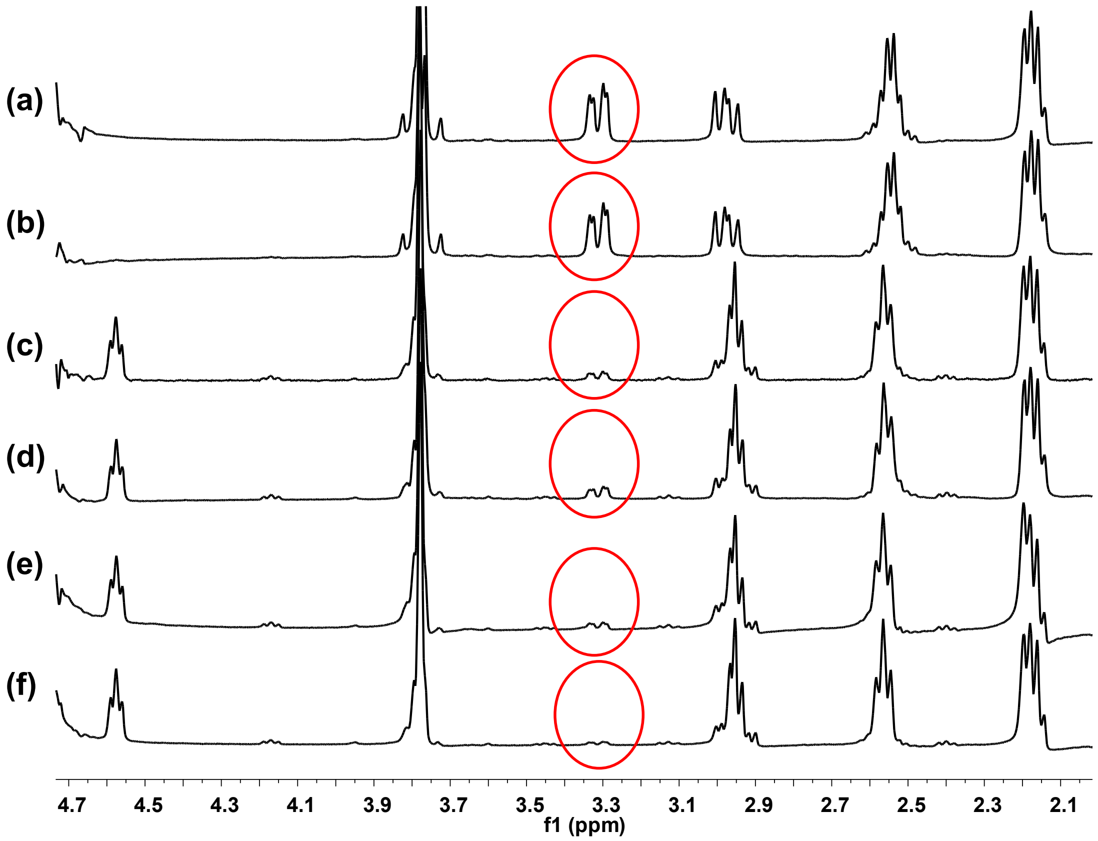

Figure S29. ^{1}H NMR spectra (400 MHz, 5% d_6-acetone/95% phosphate buffer D_2O, 30 mM, v/v, pH* 7.4) for catalysis of GSH oxidation by (a) 100 μM complex **8**; (b) 100 μM complex **7**; (c) 100 μM complex **3**; (d) 100 μM complex **1**; (e) 100 μM phenol-azopyridine (HO-azpy) ligand, for 10 mM GSH at 310 K for 24 h; (f) 10 mM GSH control without addition of the catalyst at 310 K for 24 h. Peaks at δ 3.30 ppm in the red circles are assigned to a β-CH_2 proton of GSSG. TONs were based on the integrals of GSSG peaks at δ 3.33-3.36 ppm and GSH peaks at δ 2.90-3.03 ppm (details in section **S2.12**). These experiments were repeated three times independently with similar results.

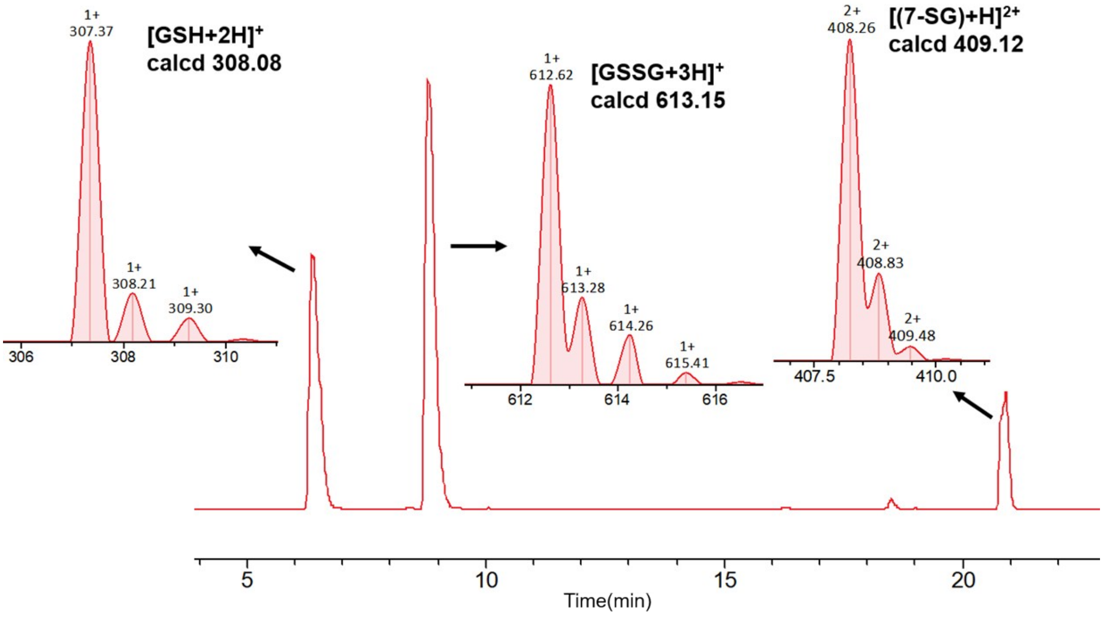

Figure S30. HPLC-ESI-MS analysis of reactions of **7** (100 μM) with GSH (10 mM) in aerated water after 24 h at 310 K. Eluents: A-water (without TFA), B-acetonitrile (without TFA). 0-10 min, 2%-15% B; 10-40 min, 15%-80% B; 40-45 min, 80%-2% B; 40-55 min, 2% B.

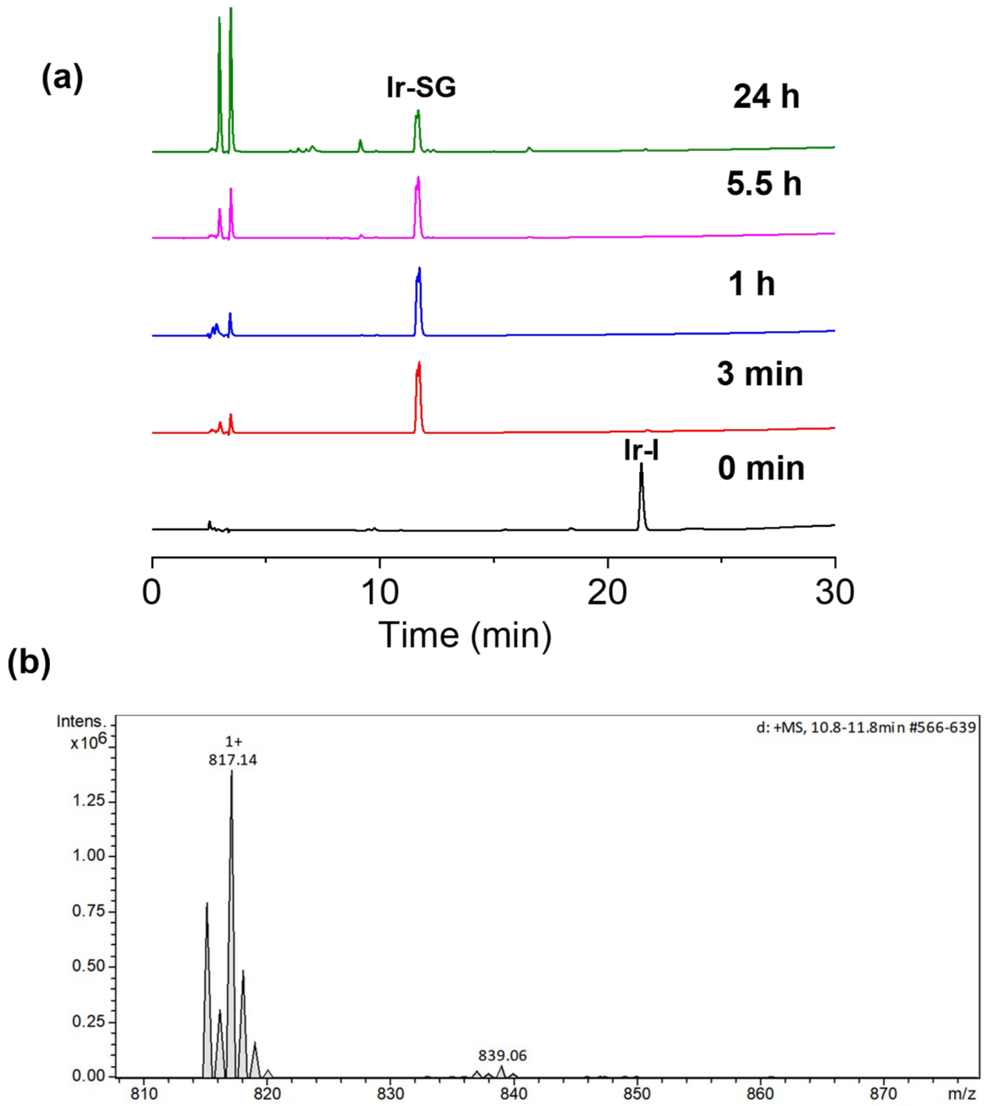

Figure S31. (a) Time-dependent HPLC chromatograms for reaction of complex **7** (100 μM) with GSH (10 mM) in 5% methanol-95% H_2O (30 mM phosphate buffer, v/v, pH 7.4) at 310 K. Eluents: A-water (0.1% v/v TFA), B-acetonitrile (0.1% v/v TFA). 0-30 min, 10%-80% B; 30-40 min, 80% B; 40-41 min, 80%-10% B; 40-55 min, 10% B. (b) The ESI-MS of the HPLC peak with *m/z* at 817.14 (calcd 817.23 for [(**7-SG**)+H]$^+$ with a retention time of *ca.*12 min is assignable to **7-SG**.

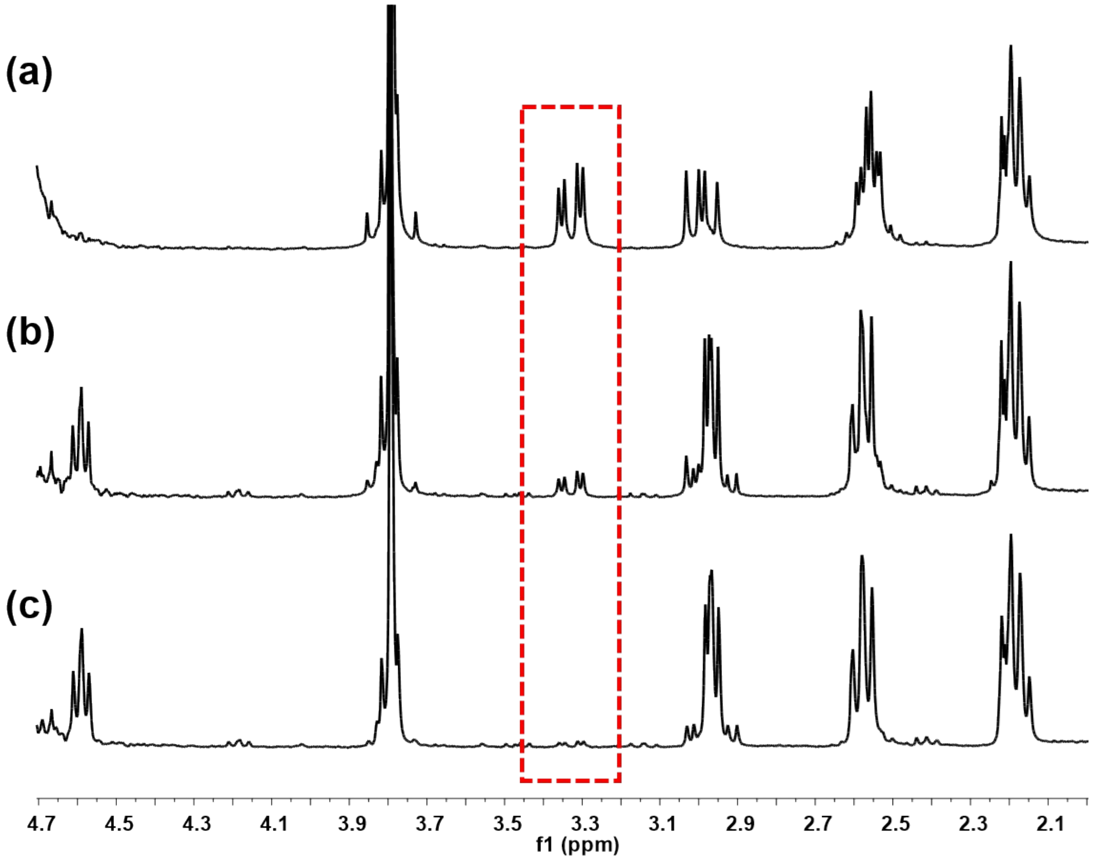

Figure S32. ^{1}H NMR spectra (400 MHz, 5% d_6-acetone/95% phosphate buffer D_2O, 30 mM, v/v, pH* 7.4) for catalysis of GSH oxidation by (a) 100 μM complex **7-SG**; (b) 100 μM complex **3-SG**, for 10 mM GSH at 310 K for 24 h; (c) 10 mM GSH alone at 310 K for 24 h. Peaks at δ 3.30 ppm in the red rectangle are assigned to a β-CH_2 proton of GSSG. TONs were based on the integrals of GSSG peaks at δ 3.30-3.36 ppm and GSH peaks at δ 2.90-3.03 ppm (details in section **S2.12**). The **7-SG** and **3-SG** gave TONs of 92 ± 1 and 18 ± 3, respectively, similar with the TONs of their corresponding parent iodido complexes **7** and **3** (Figure S29). These experiments were repeated independently three times with similar results.

(a)

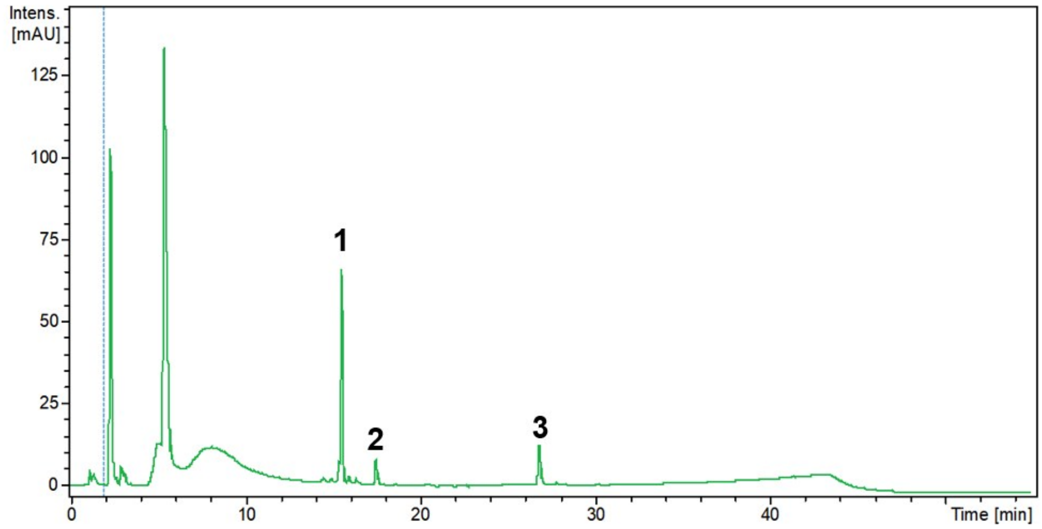

(b)

Peak assignment	Detected m/z	Calcd m/z
$[(Cp^*Ir)_2(\mu\text{-}SG)_3\text{+}3H]^{2+}$ (peak 1)	787.02	786.69
$[(H_2azpy)\text{+}H]^{2+}$ (peak 2)	185.72	186.10
$[(azpy)\text{+}H]^{2+}$ (peak 3)	183.65	184.08

Figure S33. (a) HPLC chromatogram (254 nm detection) for the reaction of **7** (100 μM) with GSH (10 mM) in oxygen-depleted water under an N_2 atmosphere after 24 h at 310 K. Eluents: A-water (without v/v TFA), B-acetonitrile (without TFA). 0-10 min, 2%-15% B; 10-40 min, 15%-80% B; 40-45 min, 80%-2% B; 40-55 min, 2% B. (b) Peak assignments based on observed and calculated ESI-MS values.

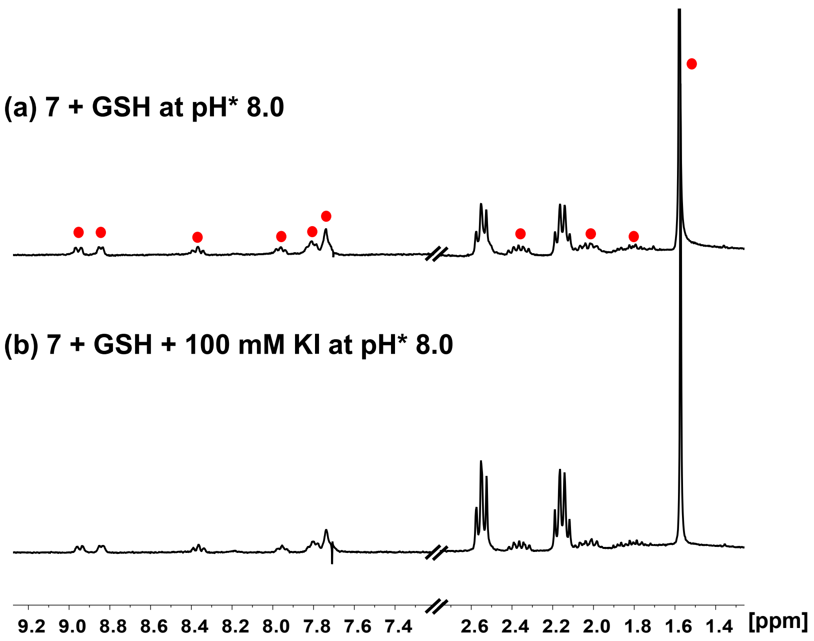

Figure S34. ^{1}H NMR (400 MHz, 298 K) spectra for reactions of complex **7** (1 mM) with 5 mol equiv. GSH in d_4-MeOD/D_2O (3/7, v/v, pH* 8.0), (a) without KI, (b) with 100 mM KI at 310 K over 10 min. Red circles: **7-SG**. The reaction of complex **7** with GSH to form **7-SG** was complete within 10 min, even in the presence of 100 mM KI, suggesting that the deprotonated Cys thiol under basic conditions can attack the azo bond and promote the release of iodide to form **7-SG**. In this case, addition of KI (100 mM) does not inhibit the formation of **7-SG**.

References

1 O. V. Dolomanov, L. J. Bourhis, R. J. Gildea, J. A. Howard and H. Puschmann, *J. Appl. Cryst.*, 2009, **42**, 339-341.

2 G. M. Sheldrick, *Acta Cryst.*, 2015, **A71**, 3-8.

3 G. M. Sheldrick, *Acta Cryst.*, 2015, **C71**, 3-8.

4 Y. Fu, A. Habtemariam, A. M. B. H. Basri, D. Braddick, G. J. Clarkson and P. J. Sadler, *Dalton Trans.*, 2011, **40**, 10553-10562.

5 A. Leo, C. Hansch and D. Elkins, *Chem. Rev.*, 1971, **71**, 525-616.

6 A. D. Becke, *J. Chem. Phys.*, 1993, **98**, 5648-5652.

7 C. Lee, W. Yang and R. Parr, *Phys. Rev, vol. B*, 1988, **37**, 785-789.

8 Gaussian 09, Revision D.01, G. W. T. M. J. Frisch, H. B. Schlegel, G. E. Scuseria, M. A. Robb, J. R. Cheeseman, G. Scalmani, V. Barone, B. Mennucci, G. A. Petersson, H. Nakatsuji, M. Caricato, X. Li, H. P. Hratchian, A. F. Izmaylov, J. Bloino, G. Zheng, J. L. Sonnenberg, M. Hada, M. Ehara, K. Toyota, R. Fukuda, J. Hasegawa, M. Ishida, T. Nakajima, Y. Honda, O. Kitao, H. Nakai, T. Vreven, J. A. Montgomery, Jr. , J. E. Peralta, F. Ogliaro, M. Bearpark, J. J. Heyd, E. Brothers, K. N. Kudin, V. N. Staroverov, T. Keith, R. Kobayashi, J. Normand, K. Raghavachari, A. Rendell, J. C. Burant, S. S. Iyengar, J. Tomasi, M. Cossi, N. Rega, J. M. Millam, M. Klene, J. E. Knox, J. B. Cross, V. Bakken, C. Adamo, J. Jaramillo, R. Gomperts, R. E. Stratmann, O. Yazyev, A. J. Austin, R. Cammi, C. Pomelli, J. W. Ochterski, R. L. Martin, K. Morokuma, V. G. Zakrzewski, G. A. Voth, P. Salvador, J. J. Dannenberg, S. Dapprich, A. D. Daniels, O. Farkas, J. B. Foresman, J. V. Ortiz, J. Cioslowski, and D. J. Fox, Gaussian, Inc., Wallingford CT, 2010.

9 S. Grimme, J. Antony, S. Ehrlich and H. Krieg, *J. Chem. Phys.*, 2010, **132**, 154104-154122.

10 P. J. Hay and W. R. Wadt, *J. Chem. Phys.*, 1985, **82**, 299-310.

11 K. Fukui, *J. Phys. Chem*, 1970, **74**, 4161-4163.

12 C. Gonzalez and H. B. Schlegel, *J. Chem. Phys.*, 1989, **90**, 2154-2161.

13 S. Miertuš, E. Scrocco and J. Tomasi, *Chem. Phys.*, 1981, **55**, 117-129.

14 S. Miertus and J. Tomasi, *Chem. Phys.*, 1982, **65**, 239-245.

15 J.-L. Pascual-ahuir, E. Silla and I. Tunon, *J. Comput. Chem.*, 1994, **15**, 1127-1138.

16 J. D. S. D. A. McQuarrie, Molecular Thermodynamics, University Science Books, Sausalito, 1999.

17 R. W. Ashcraft, S. Raman and W. H. Green, *J. Phys. Chem. B*, 2007, **111**, 11968-11983.

18 S. J. Dougan, M. Melchart, A. Habtemariam, S. Parsons and P. J. Sadler, *Inorg. Chem.*, 2006, **45**, 10882-10894.

19 Z. Liu and P. J. Sadler, *Acc. Chem. Res.*, 2014, **47**, 1174-1185.

20 J. M. Fontmorin, R. C. Burgos Castillo, W. Z. Tang and M. Sillanpää, *Water Res.*, 2016, **99**, 24-32.
